# Tetrafluorenofulvalene as a sterically frustrated open-shell alkene

**DOI:** 10.1038/s41557-023-01341-8

**Published:** 2023-10-02

**Authors:** Bibek Prajapati, Madan D. Ambhore, Duy-Khoi Dang, Piotr J. Chmielewski, Tadeusz Lis, Carlos J. Gómez-García, Paul M. Zimmerman, Marcin Stępień

**Affiliations:** 1 Wydział Chemii, Uniwersytet Wrocławski, Wrocław, Poland; 2 Department of Chemistry, University of Michigan, Ann Arbor, MI USA; 3 Departamento de Química Inorgánica and Instituto de Ciencia Molecular, Universidad de Valencia, Paterna, Spain

**Keywords:** Structure elucidation, Computational chemistry, Spectrophotometry

## Abstract

Electronic and steric effects are known to greatly influence the structure, characteristics and reactivity of organic compounds. A typical *π* bond is weakened by oxidation (corresponding to the removal of electrons from bonding orbitals), by reduction (through addition of electrons to antibonding orbitals) and by unpairing of the bonding electrons, such as in the triplet state. Here we describe tetrafluorenofulvalene (TFF), a twisted, open-shell alkene for which these general rules do not hold. Through the synthesis, experimental characterization and computational analysis of its charged species spanning seven redox states, the central alkene bond in TFF is shown to become substantially stronger in the tri- and tetraanion, generated by chemical reduction. Furthermore, although its triplet state contains a weaker alkene bond than the singlet, in the quintet state its bond order increases substantially, yielding a flatter structure. This behaviour originates from the doubly bifurcated topology of the underlying spin system and can be rationalized by the balancing effects of benzenoid aromaticity and spin pairing.

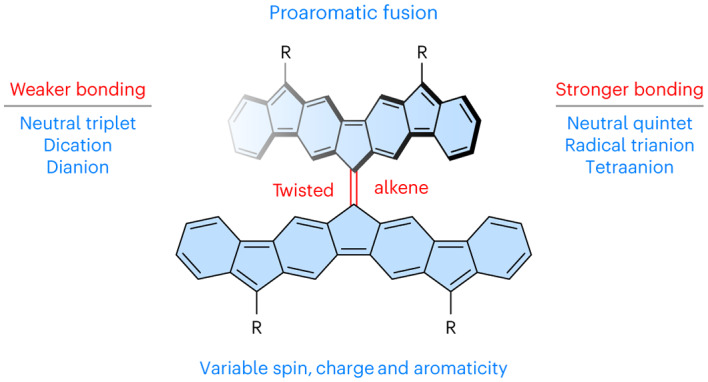

## Abstract (web summary)

Tetrafluorenofulvalene (TFF) defies conventional rules of bond strength in organic chemistry. In particular, the central alkene bond of TFF becomes stronger in the quintet state and in the tetraanion. These changes arise from the unusual interplay between the twist, aromaticity and spin pairing in the *π*-electron system of TFF.

## Main

The interplay between electronic conjugation and the geometrical distortion of *π* bonds is of fundamental importance in organic chemistry because of its role in defining the properties of chromophores^1^, organic semiconductors^2,3^, chemical reagents^4^ and molecular machinery^5^. In nature, double-bond isomerization forms the functional basis of the retinoid cycle responsible for human vision^6^, with fine-tuning provided by molecular distortions of the chromophore^7^. In the laboratory, twistable double bonds have been incorporated into molecular switches and motors^5^, switchable chromophores^2,8–10^ and organic magnetic materials^11–17^. The performance of such molecular devices critically depends on the electronic and steric features of the *π* system containing the switchable bond. All of these applications have created considerable interest in the properties of distorted *π* systems, ranging from simple alkenes^18,19^ to complex polycyclic aromatics^20–23^.

**Fig. 1:**
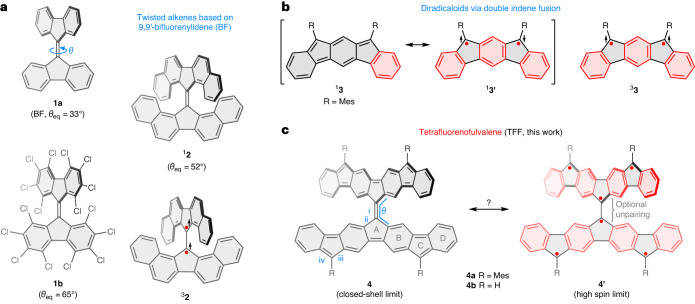
The design of tetrafluorenofulvalene. **a**–**c**, Combining the *π*-extended twisted alkenes motif found in bifluorenylidene and its derivatives (**a**) with the concept of indene fusion (**b**) produces tetrafluorenofulvalene (TFF) (**c**), a sterically frustrated alkene with an increased multiconfigurational character. Clar sextets and unpaired electrons are denoted respectively by red bonds and red dots, and the relative orientations of spins are indicated with black arrows. Key structural parameters, torsions (*θ*_eq_) and bonds (i–iv) are labelled in blue. For all systems discussed in the paper, the torsion (twist) angle *θ* was calculated as the mean of the two torsions defined by atoms 8a–9–9'–8'a and 9a–9–9'–9'a of the BF substructure.

The equilibrium twist angle *θ*_eq_ and the isomerization barrier of an alkene are interrelated and can be controlled by changes of the bond order and by steric effects. The double bond can be weakened by both oxidation^24,25^ and reduction^2,26–28^, as well as by introducing permanent twist to its ground-state structure^29,30^. Highly distorted alkenes have been developed from 9,9′-bifluorenylidene (**1a**; Fig. 1a) as well as some closely related fused frameworks^31–34^. The twist of **1a**^35^ is increased by bulky substitution^8,36,37^ or ring fusion^38–41^, as illustrated by **1b**^36^ and **2**^38–40^, respectively. The large twist in **1b** and **2** results in elongation of the respective double bonds, reduction of electronic energy gaps, and decreased isomerization barriers.

Simple alkenes become non-planar in the triplet state^42,43^, and some of the intrinsically twisted systems have thermally accessible triplets (for example, ^3^**2**). These observations suggest a possible strategy for controlling the alkene twist by increasing open-shell contributions to the ground state. We reasoned that such an influence can be studied by hybridizing a twisted alkene system with appropriately chosen stable oligoradicaloid motifs, such as those obtainable by indene fusion^44–46^. This approach leads to proaromatic systems such as **3** (Fig. 1b)^47^, which derive their stability in the singlet and triplet states from open-shell contributions containing an increased number of Clar sextets.

By applying quadruple indene fusion to 9,9′-bifluorenylidene, we have now obtained the twisted hydrocarbon tetrafluorenofulvalene (TFF, **4**), in which the properties of the centre alkene bond show a uniquely complex dependence on the electronic state of the *π* system. A fully closed-shell (Kekulé-like) valence structure can be drawn for **4** (Fig. 1c); however, this is devoid of any Clar sextets. In fact, only one double-bond localization pattern exists for **4**, implying a purely olefinic (non-aromatic) character of the closed-shell contribution. Given the presence of six five-membered rings in **4**, it is possible to draw a range of open-shell configurations, containing up to six radical centres and up to eight Clar sextets (**4**′; Fig. 1c). Their relative contributions define the degree of spin pairing in the system, which in turn affects the central alkene bond. The twist of the alkene is further modified by changes of the oxidation level, which span a range of seven redox states. We show that the behaviour of the system can be rationalized by analysing changes in its aromaticity and electron pairing using a simple, yet general, valence bond model.

## Results and discussion

### Synthesis and properties

**Fig. 2:**
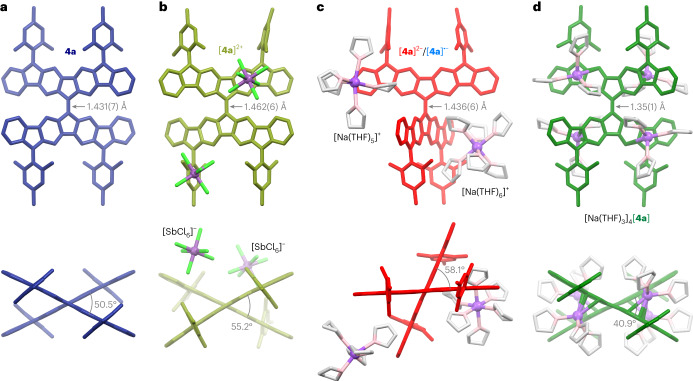
Structures of TFF at different oxidation levels, determined in X-ray diffraction analyses. **a**, Structure of **4a**. **b**, Structure of [**4a**]^2+^ (one of two non-equivalent dications, shown with two proximal [SbCl_6_]^−^ anions). **c**, Structure of [**4a**]^2−^/[**4a**]^**•**−^ (shown with two distinct [Na(THF)_*n*_]^+^ countercations; for details, see main text). **d**, Structure of [**4a**]^4−^ (shown with the coordinated [Na(THF)_3_]^+^ subunits). Hydrogen atoms, minor disordered positions and solvent molecules have been omitted for clarity. The effect of the oxidation state of TFF on the strength of the double bond is illustrated by changes of alkene bond length and *θ*_eq_ torsions (grey labels, averaged values are given for *θ*_eq_).

The mesityl-substituted TFF derivative **4a** was obtained in a four-step procedure (described in the Supplementary Information) and completely characterized. In the solid state (Fig. 2a), the two diindenofluorenylidene (DIF) subunits of **4a** form a twist angle *θ*_eq_ of 50.5°, which is larger than the corresponding torsion in **1a** (33°), and comparable to that in the sterically congested **2**. However, with a length of 1.431(7) Å, the central alkene bond i is appreciably elongated in comparison with unhindered 9,9′-bifluorenylidene (BF) derivatives (1.36–1.38 Å)^35,37,48^, and is even longer than the corresponding bond in **2** (1.40–1.41 Å)^40^. Because the ii bonds in **4a** (1.422(3) Å) are also shorter than those in the reported unhindered BF systems (1.46–1.48 Å), the central double bond in **4a** is weakened by conjugation within the DIF subunits. Furthermore, distance iii, which corresponds to a formal single bond in the closed-shell configuration of **4a**, is shorter than the formally double bond iv (1.398(4) Å versus 1.456(4) Å). Thus, the solid-state bond length pattern suggests considerable open-shell contributions to the electronic structure of TFF.

Compound **4a**, which is NMR-silent in solution, revealed a persistent electron spin resonance (ESR) signal in solid samples of **4a**, the intensity of which decreased with decreasing temperature. The temperature dependence of the ESR signal was modelled using the Bleaney–Bowers equation, yielding an estimate of the singlet–triplet gap Δ*E*_S–T_ of −1.5(1) kcal mol^−1^. Superconducting quantum interference device analysis confirmed that **4a** is a ground-state singlet, with Δ*E*_S–T_ = −3.1(1) kcal mol^−1^ calculated using the Bleaney–Bowers model. Absorption spectra of the deep-blue **4a** contained several strong maxima in the range of 400–800 nm and a weak band tailing beyond 1,000 nm, corresponding to a relatively small energy gap of ~1.2 eV. In line with the latter observation, **4a** displayed pronounced redox amphoterism in electrochemical experiments (Supplementary Fig. 1). In differential pulse voltammetry, three oxidation events were found at 0.01, 0.16 and 1.03 V (versus Fc^+^/Fc in dichloromethane). Reduction of **4a** occurred at −1.07 (2e), −1.90 (1e) and −2.01 V (1e), respectively.

**Fig. 3:**
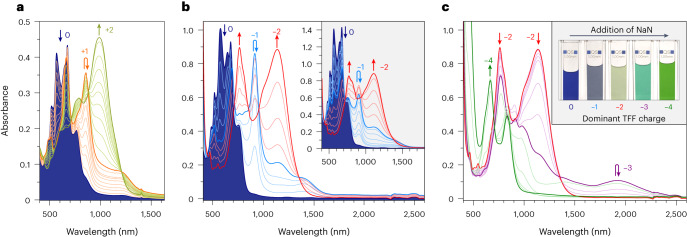
Absorption spectra of 4a and its oxidized and reduced forms. These experiments reveal the redox amphoterism of TFF and the strong near-infrared absorption of its multiple oxidation levels. **a**, Oxidation of **4a** to [**4a**]^**•**+^ and [**4a**]^2+^ (0–2 equiv. BAHA, DCM). **b**, Reduction of **4a** to [**4a**]^**•**−^ and [**4a**]^2−^ (0–4 equiv. NaN, 50 equiv. 15-crown-5, THF). Inset: reduction of **4a** to [**4a**]^**•**−^ and [**4a**]^2−^ (0–4 equiv. CoCp_2_, THF). **c**, Reduction of [**4a**]^2−^ to [**4a**]^**•**3−^ and [**4a**]^4−^ (4–24 equiv. NaN, 50 equiv. 15-crown-5, THF). Inset: colour changes observed during reduction with sodium naphthalenide (values correspond to the charge of the major TFF form present in solution). In all panels, the spectrum of **4a** is shown as a filled blue contour. Spectra corresponding to maximum concentrations of specific charged states are indicated with bold curves. Coloured arrows indicate the direction of change caused by addition of the titrant. For theoretical simulations of these spectra, see Supplementary Figs. 24–30.

Titration of **4a** with a one-electron oxidant, tris(4-bromophenyl)ammoniumyl hexachloroantimonate (BAHA, *E*_ox_ = 0.7 V in dichloromethane (DCM)) revealed consecutive formation of two NIR-absorbing species, with each step yielding near-perfect isosbestic points (Fig. 3a). The neutral **4a** could be quantitatively recovered by reduction with KO_2_. The two oxidation products were also generated electrochemically (Supplementary Figs. 15 and 16), and identified, respectively, as the radical cation [**4a**]^**•**+^ (*λ*_max_ = 860 nm) and dication [**4a**]^2+^ (*λ*_max_ = 985 nm). The absorption of the radical cation features a shoulder at ~1,200 nm, implying a smaller energy gap in [**4a**]^**•**+^ than in [**4a**]^2+^. In contrast to the neutral **4a**, the dication [**4a**]^2+^ is diamagnetic. In its ^13^C NMR spectrum, the C11 resonance was identified at a highly downfield position of 184 ppm, indicating partial localization of the positive charge at this site. The structure of the dication was further elucidated in an X-ray diffraction analysis of a single crystal of the [**4a**][SbCl_6_]_2_ salt (Fig. 2b). In comparison with the neutral **4a**, the i bond is somewhat lengthened (to 1.462(6) Å), and the *θ*_eq_ torsion increases to 54.5–55.8°. These changes suggest that the two-electron oxidation results in a moderate decrease of the i bond order.

Even though a two-electron reduction event had been revealed in electrochemical measurements, titration of **4a** with cobaltocene (CoCp_2_, *E*_red_ = −1.3 V) provided evidence for stepwise electron transfer (Fig. 3b, inset). Specifically, upon addition of up to 4 equiv. of CoCp_2_, we observed initial formation of the radical anion [**4a**]^•−^ (*λ*_max_ = 685, 920, 1,155 and ~1,355 nm). Further addition produced the dianion [**4a**]^2−^ (*λ*_max_ = 760 and 1,120 nm), which could be oxidized back to **4a** using diiodine. A broader range of anionic states of **4a** was achievable by reduction with sodium naphthalenide (NaN) in the presence of 15-crown-5 (Fig. 3b,c). Initial spectra, observed in the range of 0–9 formal equivalents of added NaN, corresponded to the sequential formation of [**4a**]^**•**−^ and [**4a**]^2−^. On further addition of NaN (~17 equiv.), we observed the formation of a new species, with near-infrared (NIR) absorptions extending beyond 2,000 nm, which was presumed to be the radical trianion [**4a**]^**•**3−^. When an even larger excess of NaN was added (up to 33 equiv.), these characteristic bands disappeared, and the final spectrum had an absorption onset at ~1,200 nm. Thus, the ultimate reduced product had a larger energy gap than all the preceding forms and was tentatively assumed to be the tetraanion [**4a**]^4−^. Both [**4a**]^4−^ and the dianion [**4a**]^2−^ could be selectively generated on a larger scale and characterized using ^1^H NMR (Supplementary Figs. 2 and 7–11).

Single crystals containing [**4a**]^2−^ and [**4a**]^4−^ anions were grown from tetrahydrofuran (THF) solutions of **4a** reduced with sodium metal in the absence of the crown ether additive. The tetraanion structure [Na(THF)_3_]_4_[**4a**] revealed a highly regular pattern of Na cations coordinated directly to the *π* system (Fig. 2d). The cations are bound near the fused edges of the BF core, with the shortest Na···C distances of 2.636(6) Å. Apparently, binding to the five-membered rings, which are presumed to carry a substantial portion of the negative charge, is not feasible because of steric protection by the Mes substituents. The i bond in the tetraanion is stronger than in other oxidation levels of **4a**, as evidenced by its short length of 1.35(1) Å and the smaller *θ*_eq_ torsion of 40.9°. Partial reduction of **4a** yielded crystals with a stoichiometry of [Na(THF)_6_][Na(THF)_5_]_0.74_[**4a**]·8.3THF, indicating a mixed-valence character of **4a**. Sodium occupancies indicate that the crystal contains mostly the dianion [**4a**]^2−^ with an ~26% admixture of the radical anion [**4a**]^**•**−^. The structure is notable for the lack of Na···*π* coordination, reflecting the smaller negative charge residing in the *π* system. Specifically, two non-equivalent sodium sites were found: octahedral [Na(THF)_6_]^+^ and disordered square-pyramidal [Na(THF)_5_]^+^. The apparent geometry of **4a** is averaged over the two contributing redox states (−1 and −2) and features a relatively long bond i (1.436(6) Å) and a large *θ*_eq_ torsion (58.1°). These parameters may indicate weaker conjugation between the DIF subunits in [**4a**]^2−^ and [**4a**]^**•**−^ than in [**4a**]^4−^.

### Computational analysis

**Fig. 4:**
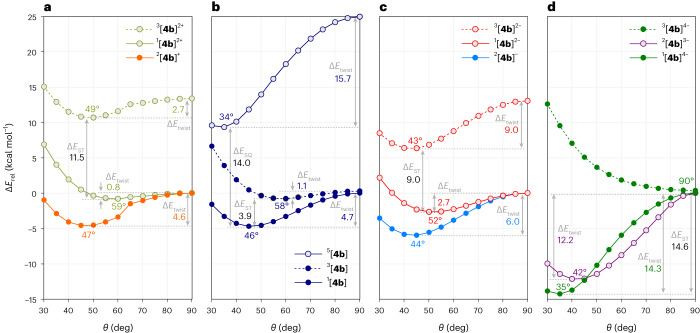
Relaxed PES scans along the torsional coordinate *θ* for relevant electronic states of TFF. **a**–**d**, Relaxed PES scans for TFF mono- and dications (**a**), neutral TFF (**b**), TFF mono- and dianions (**c**) and TFF tri- and tetraanions (**d**). These calculations, performed for the substituent-free structures ^*m*^[**4b**]^*n*^ (*n*, charge; *m*, multiplicity), show how the alkene bond strength is affected by changes of charge and spin state of TFF. Energies Δ*E*_rel_ (CAM-B3LYP/6-31G(d,p)) are given relative to the energy at *θ* = 90° obtained for the lowest multiplicity of a given charge (singlet or doublet). Coloured labels correspond to positions of energy minima (*θ*_eq_, degrees) and twist barriers (Δ*E*_twist_, kcal mol^−1^). Adiabatic singlet–triplet (ST) and singlet–quintet (SQ) gaps (Δ*E*_ST_ and Δ*E*_SQ_, respectively) are labelled in black.

Density functional theory (DFT) calculations performed for the substituent-free TFF molecule **4b** (R = H; Fig. 1c) revealed substantial variations of the key geometrical parameters as a function of the charge and multiplicity of the system (Supplementary Table 1). At the UCAM-B3LYP/6-31G(d,p) level of theory (hereafter denoted CAM), the equilibrium torsion *θ*_eq_ and central bond distance i ranged from 33.7° and 1.372 Å in ^5^[**4b**] to 90.0° 1.477 Å in ^3^[**4b**]^4−^, respectively. The i distance shows good correlation with *θ*_eq_, indicating that both parameters can be used to quantify the strength of the inter-subunit interaction in TFF. Relaxed potential energy surface (PES) scans along the *θ* coordinate revealed a complex dependence of the energy profile on the charge and multiplicity of **4b** (Fig. 4 and Supplementary Table 1). Specifically, all singlets and doublets have a twisted equilibrium geometry, characterized by *θ*_eq_ < 60° and a transition state at *θ* = 90°. The singlet dication ^1^[**4b**]^2+^ features the lowest twist barrier (Δ*E*_twist_ = Δ*E*_rel_(90°) − Δ*E*_rel_(*θ*_eq_) = 0.8 kcal mol^−1^) and the least-acute *θ*_eq_ angle (59.2°). The Δ*E*_twist_ barriers increase in the order ^1^[**4b**]^2+^ < ^1^[**4b**]^2−^ < ^2^[**4b**]^+^ < ^1^[**4b**] < ^2^[**4b**]^−^ < ^2^[**4b**]^3−^ < ^1^[**4b**]^4−^ and correlate with a decrease of the respective *θ*_eq_ angles. The above sequence can thus be assumed to reflect an increase of the bond order i. PES scans of the triplets lie above the corresponding singlet scans at all *θ* angles: a single-well potential with *θ*_eq_ = 90° is predicted for ^3^[**4b**]^4−^, while double-well potentials are found for ^3^[**4b**]^2+^ and ^3^[**4b**]^2−^. The latter two species are destabilized relative to the respective singlets, but their twist barriers Δ*E*_twist_ are actually higher, implying that the i bond becomes stronger in these two triplet states. Conversely, the neutral triplet, ^3^[**4b**], has a shallow minimum at *θ*_eq_ = 58.2° with a low Δ*E*_twist_ barrier of 1.1 kcal mol^−1^, implying a particularly weak i bond. This behaviour could be considered typical of an alkene; however, in quintet state ^5^[**4b**], the bond is predicted to be very strong, with a much higher twist barrier of 15.7 kcal mol^−1^. This unusual response of the inner alkene in the neutral **4b** to changes of spin multiplicity is further confirmed by the torsional dependence of the i and ii bond lengths (Supplementary Fig. 16). The triplet ^3^[**4b**] and quintet ^5^[**4b**] approach the limits of a pure single and double i bond, respectively, whereas the singlet ^1^[**4b**] contains a strongly conjugated alkene with an intermediate bond order.

Adiabatic singlet–triplet gaps predicted for even-electron ions of **4b** at the CAM level are relatively large (approximately −9 to −15 kcal mol^−1^; Fig. 4 and Supplementary Table 1), in line with the observed diamagnetism of [**4a**]^2+^, [**4a**]^2−^ and [**4a**]^4−^. For the neutral **4b**, a markedly smaller gap was obtained (Δ*E*_ST_ = −3.91 kcal mol^−1^), relatively close to the experimental superconducting quantum interference device value, whereas the quintet state ^5^[**4b**] was predicted to have a much higher energy (Δ*E*_SQ_ = −14 kcal mol^−1^). Because CAM, as a single-reference method, is not fully suitable for quantitative assessment of spin-state energetics, we evaluated energies of the neutral ^*m*^[**4b**] (*m* = 1, 3, 5) using two active-space methods, that is, CAS-SCF(6,6)/6-31G(d,p) (denoted CAS), and the spin-flip approach^49,50^ at the RAS(4,4)-SF-*sr*B3LYP/cc-pVDZ level of theory (denoted RAS). RAS calculations, performed for the CAM-optimized minima and PES scans, indicate that the ST and SQ gaps may be smaller than predicted by the CAM level (approximately −3 and −7 kcal mol^−1^, respectively; Supplementary Fig. 18).

An analysis of natural orbital occupation numbers (NOONs) showed that the neutral singlet ^1^[**4b**] had a pronounced tetraradicaloid character, as revealed by the values of di- and tetraradicaloid indexes, *y*_0_ ≥ 0.98 and *y*_1_ ≥ 0.29, respectively, with a possible smaller hexaradicaloid contribution (*y*_2_^CAM^ = 0.12). The number of unpaired electrons obtained from the CAM-derived NOONs (*n*_U_^CAM^; Supplementary Table 1) is non-zero for all states except for ^1^[**4b**]^4−^, which is the only one with a purely closed-shell configuration. The *n*_U_^CAM^ value of 3.15 obtained for the neutral singlet ^1^[**4b**] is in fact higher than in the corresponding triplet state. Extensive mixing of open-shell configurations is indicated by the high values of *n*_U_^CAM^ (that is, exceeding *m* − 1) obtained for ^2^[**4b**]^+^ and ^2^[**4b**]^−^. Because twisting of an alkene normally leads to *π*-bond breaking, one could intuitively expect the *n*_U_ values to increase with increasing *θ*. However, no such general relationship is found for **4b** (Supplementary Fig. 17). Paradoxically, *n*_U_ decreases with *θ* for the neutral singlet ^1^[**4b**], as well as for ^2^[**4b**]^−^, ^2^[**4b**]^+^, ^1^[**4b**]^2−^ and ^1^[**4b**]^2+^, suggesting that in these species, electron pairing is actually enhanced by decoupling of the DIF subunits.

**Fig. 5:**
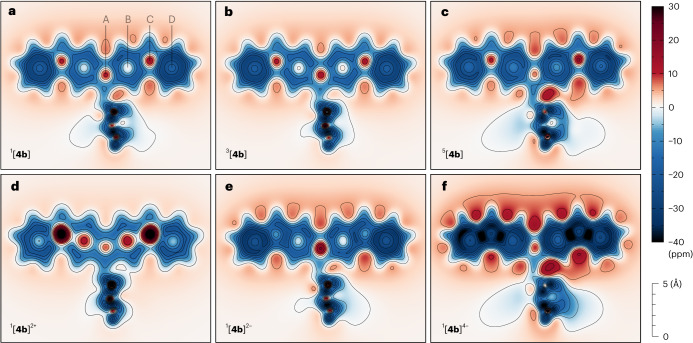
2D NICS scans performed for selected oxidation levels and multiplicities of 4b. **a**–**c**, Spin states of the neutral TFF (^1^[**4b**] (**a**), ^3^[**4b**] (**b**) and ^5^[**4b**] (**c**)) show an unusual sequence of aromaticity changes (quintet > singlet > triplet), resulting from differential mixing of di- and tetraradicaloid configurations. **d**–**f**, Even more pronounced changes of the aromatic character are observed in TFF ions (^1^[**4b**]^2+^ (**d**), ^1^[**4b**]^2−^ (**e**) and ^1^[**4b**]^4−^ (**f**)). The cross-sectional plane (CSP) was located 1 Å above the plane of one of the DIF subunits (the other DIF subunit, located in the bottom half of each figure, is tilted relative to the CSP). NMR shieldings were evaluated along the normal of the CSP using the CAM level of theory. Centres of rings A–D (cf. Fig. 1c) are labelled in **a**. Maps of reference systems are provided in Supplementary Fig. 23.

Nucleus-independent chemical shifts (NICS) revealed striking variations of magnetism in **4b** caused by changes of its oxidation and spin state (Fig. 5 and Supplementary Fig. 23). Although the triplet state ^3^[**4b**] is less aromatic than the singlet ^1^[**4b**], the quintet ^5^[**4b**] state shows enhanced aromaticity. The NICS map obtained for the triplet is essentially identical to the map obtained for the diindenofluorenyl radical ^2^[**DIF-H**], consistent with the weak interaction between DIF subunits in ^3^[**4b**] found in the PES scan. Thus, the enhancement of aromaticity in the singlet and quintet originates from the stronger inter-subunit coupling in each of these two spin states. This conclusion is supported by the harmonic oscillator model of aromaticity (HOMA; Supplementary Table 2), which produced higher indexes of rings B in ^5^[**4b**] (0.89) and ^1^[**4b**] (0.71) than in ^3^[**4b**] (0.63). The doubly charged ^1^[**4b**]^2+^ and ^1^[**4b**]^2−^ show opposite changes of their magnetism, being respectively para- and diatropic. The tetraanion ^1^[**4b**]^4−^ experiences a dramatic increase in the aromaticity of rings C–D, confirming that it can be treated as a union of four fluorenyl anions. In line with this interpretation, the HOMA indices for rings B, C and D, are 0.76, 0.42 and 0.76.

### Valence structure of TFF

**Fig. 6:**
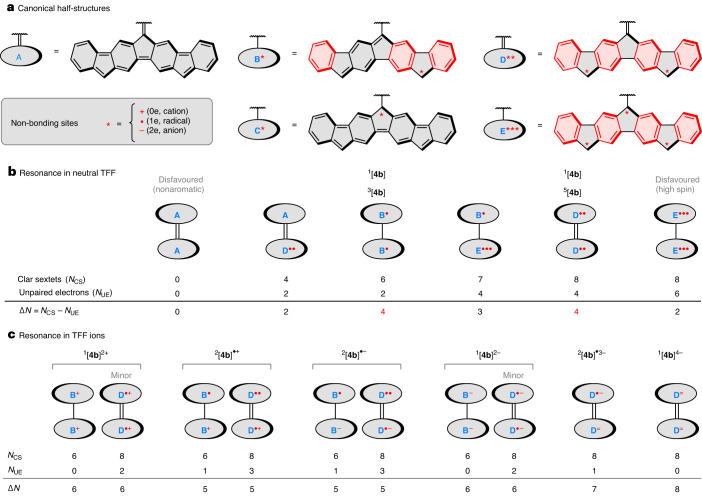
Valence structure of TFF and its ions. **a**, Half-structures **A**–**E** used to assemble resonance contributors of neutral and charged states. Each non-bonding site (denoted *) can contain 0, 1 or 2 electrons. **b**, Resonance in the neutral TTF: the preference for the diradicaloid and tetraradicaloid configurations is controlled by the spin state, explaining the changes in alkene bond order. **c**, Preferred resonance contributors in charged states of TTF (D^=^ denotes a dianionic half-structure). For full representations of the resonance contributors, see Supplementary Fig. 37.

A unified description of the valence structure of TFF, which is valid for all oxidation levels, can be developed using the five canonical half-structures **A** through E*** shown in Fig. 6a. These structures differ in (1) the number of Clar sextets, (2) the number of formally non-bonding sites (denoted with an asterisk) and (3) the order of the linking bond (single or double). For example, there are no Clar sextets and no non-bonding sites in structure **A**, whereas structure B* features three sextets and one non-bonding site, that is, either a cation, a radical or an anion. Resonance contributors of TFF can be constructed from half-structure pairs with matching linking bond orders, for example **A** + **A** or B* + B* (Fig. 6b,c). Each such contributor can be characterized by the total number of sextets (*N*_CS_) and the number of unpaired electrons (*N*_UE_). Given that the stability of the structures is expected to increase for high *N*_CS_ values and low *N*_UE_ values, one can consider a simple stability metric Δ*N* = *N*_CS_ − *N*_UE_.

Data obtained for the neutral TFF indicate that the triplet state ^3^[**4b**] is well approximated by the diradical structure **B**^**•**^–**B**^**•**^, containing a single bond between the DIF units. Similarly, the tetraradicaloid form **D**^**••**^=**D**^**••**^ provides an accurate representation of the quintet ^5^[**4b**]. Mixing of these two contributions in ^1^[**4b**] can be proposed to explain the intermediate aromaticity, inter-subunit bond order and high tetraradicaloid character of the singlet state. In particular, **B**^**•**^–**B**^**•**^ and **D**^**••**^=**D**^**••**^ have the highest Δ*N* = 4 among all canonical forms, which explains their relative importance. Doubly charged TFF ions ^1^[**4b**]^2+^ and ^1^[**4b**]^2−^ can be similarly characterized with singly bonded structures **B**^+^–**B**^+^ and **B**^−^–**B**^−^, respectively; however, small contributions of the doubly bonded forms **D**^**•**−^=**D**^**•**−^ and **D**^**•**+^=**D**^**•**+^ need to be invoked to explain the non-zero *n*_U_ values and non-vanishing inversion barriers of these two species. The latter two forms should become dominant in the structures of respective triplets ^3^[**4b**]^2+^ and ^3^[**4b**]^2−^, explaining the high Δ*E*_twist_ values predicted for these species. Analogous contributions become even more relevant in the singly charged ^2^[**4b**]^+^ and ^2^[**4b**]^−^ (**D**^**••**^=**D**^**•**−^ and **D**^**••**^=**D**^**•**+^, respectively), both of which are notable for their triradicaloid character (*n*_U_ > 2.5). Finally, the doubly bonded contributors fully dominate in the higher anions, ^2^[**4b**]^3−^ and ^1^[**4b**]^4−^, which feature the highest Δ*E*_twist_ barriers, and very low *n*_U_ values.

## Conclusion

In this Article, we have shown how the sterically frustrated alkene bond in TFF is affected by the open-shell nature of the *π*-conjugated system. The underlying network of radicaloid sites in TFF is neither linear nor cyclic and features a unique doubly bifurcated topology with a centrally positioned formal double bond. The strength of the alkene bond is controlled by mixing of oligoradicaloid configurations in the neutral singlet state, by electron unpairing in high-spin states, and by electron transfer in the oxidized and reduced forms of TFF. Changes of the oxidation level, spanning seven consecutive states, result in profound alteration of the spectroscopic signatures of this unusual *π* system. The pivotal role of the central alkene bond in controlling the *π* conjugation in TFF suggests that, by using similar design principles, it may be possible to create molecular organic materials that will change their spin state, redox potentials and optical characteristics in response to mechanical stimuli.

## Online content

Any methods, additional references, Nature Portfolio reporting summaries, source data, extended data, supplementary information, acknowledgements, peer review information; details of author contributions and competing interests; and statements of data and code availability are available at 10.1038/s41557-023-01341-8.

### Supplementary information

Supplementary Information Methods, synthetic procedures, Supplementary figures and tables.

Supplementary Data 1 Crystallographic data for compound (**4a**·C_6_H_14_); CCDC reference 2250914

Supplementary Data 2 Crystallographic data for compound ([**4a**]^2+^[SbCl_6_^–^]_2_); CCDC reference 2209267

Supplementary Data 3 Crystallographic data for compound ([Na(THF)_6_][Na(THF)_5_]_0.74_[**4a**]); CCDC reference 2209269

Supplementary Data 4 Crystallographic data for compound ([Na(THF)_3_]_4_[**4a**]); CCDC reference 2209268

## Data Availability

All relevant data are available within the paper and its Supplementary Information files. Cartesian coordinates of calculated structures as well as source data for Fig. 4 and Supplementary Figs. 12, 16–18 and 21 have been deposited at Zenodo (10.5281/zenodo.8075979). Crystallographic data for the structures reported in this Article have been deposited at the Cambridge Crystallographic Data Centre, under deposition nos. 2250914 (**4a**·C_6_H_14_), 2209267 ([**4a**]^2+^[SbCl_6_^−^]_2_), 2209269 ([Na(THF)_6_][Na(THF)_5_]_0.74_[**4a**]) and 2209268 ([Na(THF)_3_]_4_[**4a**]). Copies of the data can be obtained free of charge via https://www.ccdc.cam.ac.uk/structures/.
