# Supplementary material for: Tetrafluorenofulvalene as a sterically frustrated open-shell alkene
Source: Nat Chem. 2023 Oct 2;15(11):1541–8. doi: 10.1038/s41557-023-01341-8 (PMC10624625; doi:10.1038/s41557-023-01341-8)
Supplement: Supplementary file 1 — Methods, synthetic procedures, Supplementary figures and tables. [file 41557_2023_1341_MOESM1_ESM.pdf]

nature chemistry

Article

https://doi.org/10.1038/s41557-023-01341-8

Tetrafluorenofulvalene as a sterically frustrated open-shell alkene

In the format provided by the authors and unedited

Table of Contents

Experimental 2

Synthesis 6

Supplementary Figures 11

Supplementary Tables 45

NMR Spectra 52

Mass Spectra 56

References 60

Experimental

General. Tetrahydrofuran and 1,4-dioxane were dried using a commercial solvent purification system. Dichloromethane was distilled from calcium hydride when used as a reaction solvent. All other solvents and reagents were used as received. Compound **5**[1] was prepared according to the modified literature procedure. ^{1}H NMR spectra were recorded on high-field spectrometers (^{1}H frequency 500.13 or 600.13 MHz), equipped with broadband inverse or conventional gradient probe heads. Spectra were referenced to the residual solvent signals (chloroform-*d*, 7.24 ppm, dichloromethane-d_2, 5.32 ppm, tetrahydrofuran-d_8, 3.58 ppm, acetonitrile-d_3, 1.94 ppm). ^{13}C NMR spectra were recorded with ^{1}H broadband decoupling and referenced to solvent signals ($^{13}CDCl_3$, 77.0 ppm, $^{13}CD_2Cl_2$, 55.0 ppm, $^{13}C_4D_8O$, 67.21 ppm, $^{13}CD_3CN$, 118.26 ppm). Partial assignment of ^{1}H and ^{13}C NMR spectra of selected diamagnetic forms of TFF was achieved using correlation spectroscopy. Two-dimensional NMR spectra were recorded with 2048 data points in the t_2 domain and upto 2048 in the t_1 domain with a 1.5 s recovery delay. All 2D spectra were recorded with gradient selection except for ROESY. ROESY spinlock time were 200 -100 ms, respectively. High resolution mass spectra were recorded using MALDI and ESI ionization in the positive mode on Bruker Apex ultra-FT-ICR. UV-Vis-NIR Absorption spectrometry was performed using Perkin Elmer LAMBDA 1050 UV-NIR spectrometer. Electrochemical measurements (DCM, 0.1 M $[NBu_4][PF_6]$, 293 K) were performed on an Metrohm Autolab potentiostat/galvanostat using a glassy carbon working electrode, platinum wire as the auxiliary electrode, and silver wire as a reference electrode. The voltammograms were referenced against the half-wave potential of Fc^{+} /Fc.

Magnetic measurements. Variable temperature susceptibility measurements were carried out with a Quantum Design MPMS-XL-7 SQUID magnetometer, in the temperature range 2–370 K, with an applied magnetic field of 0.5 T, on polycrystalline samples of compounds **4a**, sealed in glass capillaries under inert atmosphere. The samples were measured in heating and cooling scans at a scan rate of 2 K/min. The data (and the fits) were very similar in the cooling and heating scans. The susceptibility data were corrected for the empty glass capillary previously measured using the same conditions and for the diamagnetic contribution of the samples as deduced by using Pascal´s constant tables.[2]

Computational methods. Density functional theory (DFT) calculations were performed using Gaussian 16.[3] DFT geometry optimizations were carried out in unconstrained C_1 symmetry, using molecular mechanics or semiempirical models as starting geometries. The calculations were performed using the hybrid functional B3LYP,[4–6] including the CAM[7] and GD3BJ[8] corrections, and the 6-31G(d,p) basis set. Unrestricted wavefunctions were used for all open-shell systems and broken-symmetry solutions were obtained for all open-shell singlets. Each structure was optimized to meet standard convergence criteria, and the existence of a local minimum was verified by a normal mode frequency calculation. The twisted conformers were confirmed to correspond to global minima by comparison with hypothetical folded (C_{2h}-symmetric) conformers (for a representative geometry, see Supplementary Figure 37). For all investigated electronic states of **4b**, the energies of the folded structures were at least 8 kcal/mol higher than those of the corresponding twisted conformations (Supplementary Table 1), indicating that the folded conformers should not be populated under the experimental conditions used in this work. The majority of electronic states of **4b** converged to perfect D_2-symmetric geometries, whereas small distortions from the D_2 point symmetry were observed for the ^{2}[**4b**]$^{\pm}$ and ^{3}[**4b**]$^{2\pm}$ ions. As an alternative, optimizations at the CAS-SCF(6,6)/6-31G(d,p) level (denoted CAS) were also tested, however, they were found to produce spurious bond localization[9] and symmetry breaking, and were unsuitable for further work. As a multiconfigurational method, the CAS approach was nevertheless useful for evaluation of open-shell characters (cf. Supplementary Table 1).

RAS-SF/cc-pVDZ[10–12] calculations utilized a (4,4) with quintet reference orbitals from RO-B3LYP. Additional electron correlation was recovered from the RAS-SF wave functions using short-range (*sr*) B3LYP corrections.[13] Nucleus-Independent Chemical Shift (NICS)[14] maps (Figure 6 and S23) were obtained at the CAM level of theory, by evaluating GIAO shieldings over a square grid of 201 × 201 points and located 1 Å above the plane of the molecule. The plots correspond to the anisotropic shielding value in the direction perpendicular to the cross-section plane. HOMA values were calculated as previously reported, using experiment-based parameters.[15,16] Oligoradicaloid indices were calculated as $y_i = 1 - (n_{HONO-i} - n_{LUNO-i})/2$.[17] Numbers of unpaired electrons n_U were calculated using the method proposed by Head-Gordon.[18]

Electronic transitions were simulated for the low-spin configurations of the chemically accessible oxidation levels of [**4b**]^n (n = +2 through –4, R = H) using time-dependent (TD) calculations at the CAM level. When corrected for the intrinsic blue-shift of the CAM method,[19] the resulting spectra were in semiquantitative agreement with the experimental absorptions observed in the course of oxidation and reduction experiments (Figures S24–S30). In particular, the neutral **4b** and [**4b**]$^{4-}$ were predicted to have the largest energy gaps, in line with the experiment. Similarly, the spectra for n = +2, and –1 through –3 were correctly predicted to contain absorption maxima in the NIR range (> 1000 nm), although the intensity of the lowest-energy transitions was apparently overestimated by the TD calculations.

X-ray crystallography. X-Ray quality crystals were grown by slow diffusion of *n*-hexane into a chloroform solution of compound **4a**. For [**4a**]$^{2+}$[$SbCl_6^-$]$_2$, crystals were grown by slow diffusion of *n*-hexane into a dichloromethane solution of dication product.

Crystallization of [**4a**]$^{2-}$[Na^+]$_2$: Inside the glove box, 1 mg of compound **4a** was dissolved in 1 mL of THF and excess of Na metal (around 50 equiv) was added to the solution. The mixture was stirred at room temperature until the color of starting material changed from deep blue to grey to green and then filtered. The filtrate was layered with 1 mL of *n*-hexane and placed at -20° C for 5 days to obtain the crystals.

Crystallization of [**4a**]$^{4-}$[Na^+]$_4$: Inside the glove box, 1 mg of compound **4a** was dissolved in 1 mL of THF and excess of Na metal (around 100 equiv) was added to the solution. The mixture was stirred for 24 hours at room temperature and then filtered. The filtrate was layered with 1 mL. of *n*-hexane and placed at -20° C for 8 days to obtain the crystals.

Diffraction data were collected on a Rigaku Oxford Diffraction XtaLAB Synergy-R DW diffractometer equipped with a HyPix ARC 150° Hybrid Photon Counting (HPC) detector using CuKα (λ = 1.5418 Å) for compound **4a** at 100 K. Data collection, cell refinement, data reduction and analysis were carried out with the Xcalibur PX software, CRYSALIS CCD and CRYSALIS RED, respectively (Oxford Diffraction Ltd., Abignon, England, 2009). An analytical absorption correction was applied with the use of CRYSALIS RED. All structures were solved by direct methods with the SHELXS-97 program and refined using SHELXL-97 with anisotropic thermal parameters for non-H atoms. In the final refinement cycles, all H atoms were treated as riding atoms in geometrically optimized positions.

Explanation of CheckCIF alerts

CCDC 2209267 ([**4a**]$^{2+}$[$SbCl_6^{-}$]$_2$)

Alert level B

PLAT220_ALERT_2_B NonSolvent Resd 1 C Ueq(max)/Ueq(min) Range 9.7 Ratio

PLAT220_ALERT_2_B NonSolvent Resd 2 C Ueq(max)/Ueq(min) Range 7.3 Ratio

Explanation: Several large displacement parameters produced by libration of mesityl substituents.

2209269 ([Na(THF)$_6$][Na(THF)$_5$]$_{0.74}$[**4a**])

Alert level B

PLAT026_ALERT_3_B Ratio Observed / Unique Reflections (too) Low .. 35% Check

Explanation: very weakly diffracting specimen (the crystals were air- and moisture-sensitive)

2209268 ([Na(THF)$_3$]$_4$[**4a**])

Alert level B

PLAT026_ALERT_3_B Ratio Observed / Unique Reflections (too) Low .. 39% Check

PLAT340_ALERT_3_B Low Bond Precision on C-C Bonds .... 0.01064 Ang.

PLAT934_ALERT_3_B Number of (Iobs-Icalc)/Sigma(W) > 10 Outliers .. 3 Check

Explanation: very weakly diffracting specimen (the crystals were air- and moisture-sensitive)

Synthesis

The mesityl-substituted TFF **4a** was obtained in a four-step three-pot procedure outlined in Supplementary Figure 35. First, 2,2',7,7'-tetrabromo-9,9'-bifluorenylidene[1] **5** was subjected to a fourfold Suzuki coupling with 2-formylphenylboronic acid, and the resulting tetraaldehyde **6** was converted into the tetrahydro precursor **7a**, which formed as a mixture of stereoisomers. Ultimately, compound **7a** was dehydrogenated using diiodine in the presence of potassium *tert*-butoxide, to produce the desired oligoradicaloid **4a** in an 80% yield. The completeness of dehydrogenation was confirmed by a mass spectrometric analysis, which revealed an M^+ molecular ion with an *m/z* ratio of 1148.5338. In the solid state, **4a** is stable for months if stored in a refrigerator. It is also sufficiently stable in solution to be purified by column chromatography under ambient conditions, but it will gradually decompose if its solutions are kept in air for several hours. For this reason, many of the subsequent experiments on **4a** were performed in an inert-atmosphere glove box.

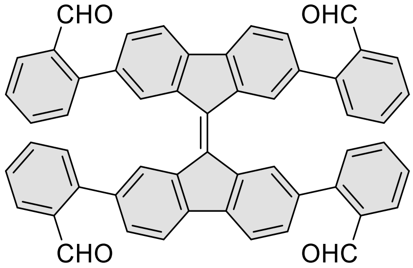

2,2',2'',2'''-([9,9'-Bifluorenylidene]-2,2',7,7'-tetrayl)tetrabenzaldehyde (6). Compound **5** (500 mg, 1.03 mmol) and 2-formyl phenyl boronic acid (811.54 mg, 5.14 mmol) was dissolved in 100 mL of dioxane and the solution was purged with nitrogen for 15 minutes. The degassed solution of sodium carbonate (1.74 g, 16.5 mmol) in 10 mL of water was added to the mixture followed by the addition of tetrakis(triphenylphosphine)palladium(0) (250.18 mg, 0.21 mmol) The mixture was purged with nitrogen for few minutes and heated at 100 °C for 24 hours. After cooling to room temperature, water was added and extracted with dichloromethane. The organic layer was washed with brine, dried over anhydrous sodium sulfate(VI) and the solvent was removed on rotary evaporator. The crude mixture was purified via silica column chromatography using 70% dichloromethane in *n*-hexane as an eluent to give compound **6** (713 mg, 93%) as a red solid. **^{1}H NMR** (600 MHz, chloroform-*d*, 300 K): δ 9.96 (4H, s), 8.53 (4H, s), 7.99 (4H, dd, ^3J = 7.6 Hz, ^4J = 1.4 Hz), 7.79 (4H, d, ^3J = 7.7 Hz), 7.49 (4H, t, ^3J = 7.5 Hz), 7.45 (4H, t, ^3J = 7.6 Hz), 7.30 (4H, dd, ^3J = 7.8 Hz, ^4J = 1.4 Hz), 7.15 (4H, d, ^3J = 7.4 Hz). **^{13}C NMR** (151 MHz, chloroform-*d*, 300 K): δ 191.75, 145.40, 141.33, 140.61, 138.40, 137.11, 133.79, 133.60, 131.94, 130.42, 128.07, 127.92, 120.10. **HRMS** (MALDI–TOF): *m/z*: $[M + Na]^+$ Calcd for $C_{54}H_{32}O_4$: 767.2193; Found 767.2273.

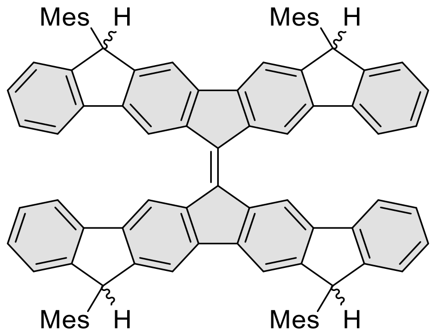

12,12',15,15'-Tetramesityl-12,12',15,15'-tetrahydro-6,6'-bi(cyclopenta[2,1-b:3,4-b'] difluorenylidene) (7a). Compound **6** (100 mg, 0.13 mmol) was dissolved in dry tetrahydrofuran (20 mL) in a flame-dried Schlenk flask and purged with nitrogen for 15 minutes. 2-mesitylmagnesium bromide (1.0 mL, 1 M in THF, 1.07 mmol) was added dropwise at room temperature and stirred overnight under nitrogen. The mixture was quenched with water, extracted with dichloromethane, dried over anhydrous sodium sulfate(VI) and evaporated to dryness. The resulting solid was dissolved in freshly distilled dichloromethane (30 mL) and purged with nitrogen for 15 minutes. 0.4 mL of borontrifluoride diethyl etherate were added slowly and stirred for 15 minutes at room temperature. The solvent was removed on a rotary evaporator and the crude mixture was purified via silica column chromatography using 25% dichloromethane in *n*-hexane as an eluent to give compound **7a** as a red solid (141 mg, 91%, mixture of stereoisomers).. **^{1}H NMR** (600 MHz, chloroform-*d*, 300 K): δ 9.05 (4H, m), 7.57 (4H, m), 7.45 (4H, m), 7.20 (8H, m), 7.06 (4H, s), 6.69 (4H, m), 5.59 (4H, m), 2.73 (4H, m), 2.73 (12H, s), 2.30 (12H, m), 1.25 (12H, m). **^{13}C NMR** (151 MHz, chloroform-*d*, 300 K): δ 149.26, 147.34, 141.23, 140.92, 140.73 139.95, 138.27, 138.08, 137.72, 136.46, 136.45, 136.38, 133.92, 133.83, 130.70, 130.58, 128.95, 127.15, 127.03, 124.15, 119.52, 119.39, 118.18, 115.79, 49.89, 21.87, 20.90, 18.83, 18.77. **HRMS** (MALDI–TOF): *m/z*: $[M]^+$ Calcd for $C_{90}H_{72}$: 1152.5629; Found 1152.5503.

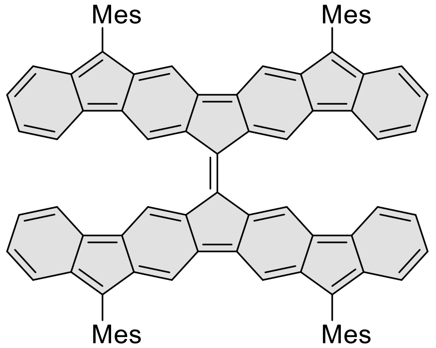

12,12',15,15'-Tetramesityl-6,6'-bi(cyclopenta[2,1-b:3,4-b']difluorenylidene) (4a). Compound **7a** (25 mg, 0.02 mmol) was dissolved in dry tetrahydrofuran (15 mL) in a flame-dried Schlenk flask and purged with nitrogen for 15 minutes. Potassium *tert*-butoxide solution (2 mol/L in 2-MeTHF, 90 µL, 0.17 mmol) was added slowly to the vigorously stirred solution at room temperature. The mixture was stirred for 5 minutes and a diiodine solution (0.1 M in THF, 480 µL, 0.04 mmol) was added dropwise with stirring. The dark-red mixture was stirred at room temperature for 15 minutes. The mixture was quenched with water, treated with 0.1 N HCl and extracted with dichloromethane. The organic layer was washed with brine, dried over anhydrous sodium sulfate(VI) and the solvent was removed on a rotary evaporator. The crude mixture was purified via quick silica column chromatography using 1:4 DCM/*n*-hexane as an

eluent to afford compound **4a** as a dark blue solid (20 mg, 80%). **HRMS** (MALDI–TOF): *m/z*: $[M]^+$ Calcd for $C_{90}H_{68}$: 1148.5316; Found 1148.5338.

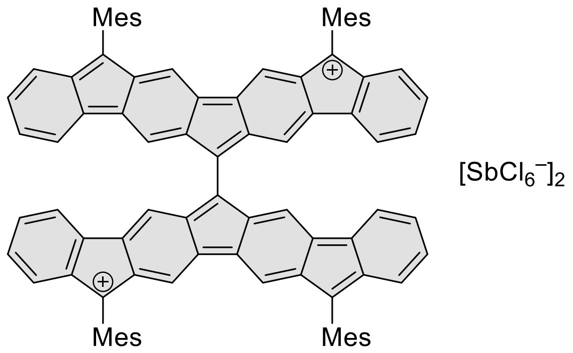

12,12',15,15'-Tetramesityl-6,6'-bi(cyclopenta[2,1-b:3,4-b']difluorenylidene) dication, hexachloroantimonate salt ($[\mathbf{4a}]^{2+}[SbCl_6^-]_2$**).** The 1 mL of dry DCM solution of tris(4-bromophenyl)ammoniumyl hexachloroantimonate, BAHA (3.13 mg, 3.83 µmol) was added dropwise into the 1 mL of dry DCM solution of compound **4a** (2.0 mg, 1.74 µmol). The mixture was stirred for 5 minutes, and the solution was passed through filtration pad. The solvent was dried under vacuum to give the dication compound $[\mathbf{4a}]^{2+}[SbCl_6^-]_2$ as a blue solid. **^{1}H NMR** (600 MHz, dichloromethane-d_2, 300 K): δ 7.23 (4H, d, ^4J = 7.3 Hz), 7.11 (4H, t, ^3J = 7.5 Hz), 7.03 (8H, s), 6.93 (4H, s), 6.89 (4H, t, ^3J = 7.5 Hz), 6.74 (4H, s), 6.70 (4H, d, ^4J = 7.3 Hz), 2.35 (12H, s), 2.29 (24H, s). **^{13}C NMR** (151 MHz, dichloromethane-d_2, 300 K): δ 184.13, 158.95, 152.11, 145.18, 142.59, 142.57, 141.69, 140.66, 139.53, 138.35, 137.35, 133.13, 131.52, 131.09, 130.61, 129.22, 125.80, 121.14, 54.99, 22.57, 22.03.

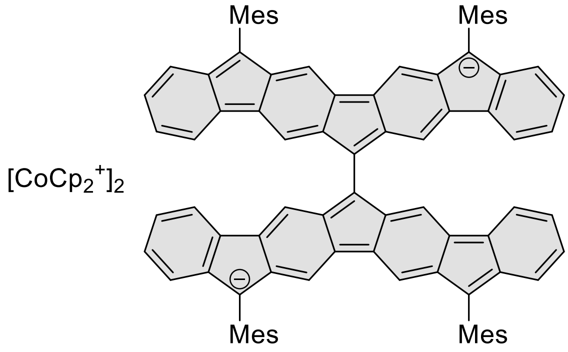

12,12',15,15'-Tetramesityl-6,6'-bi(cyclopenta[2,1-b:3,4-b']difluorenylidene) dianion, cobaltocenium salt ($[CoCp_2^+]_2[\mathbf{4a}]^{2-}$**).** Inside glove box, compound **4a** (2.0 mg, 1.74 µmol) was dissolved in 1 mL of dichloromethane and cobaltocene (1.32 mg, 6.96 µmol) was added to the solution. The mixture was stirred for 30 minutes at room temperature and then filtered. The filtrate was dried under vacuum to give dianion compound $[\mathbf{4a}]^{2-}[CoCp_2^+]_2$ as a brown solid. **^{1}H NMR** (600 MHz, acetonitrile-d_3, 300 K): δ 7.67 (4H, s), 7.32 (4H, d, ^4J = 7.4 Hz), 7.02 (8H, s), 6.80 (4 H, t, ^3J = 7.2 Hz), 6.71 (4H, s), 6.63 (8H, m), 2.35 (12H, s), 2.09 (24H, s).

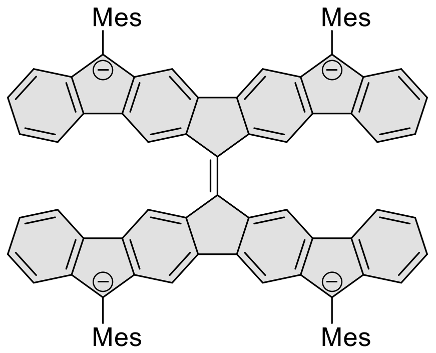

12,12',15,15'-Tetramesityl-6,6'-bi(cyclopenta[2,1-b:3,4-b']difluorenylidene) tetraanion, sodium salt ($[Na^+]_4[$**4a**$]^{4-}$**).** Inside a glove box, compound **4a** (2.0 mg, 1.74 µmol) was dissolved in 1 mL of THF and freshly prepared sodium naphthalenide solution (0.025 M in THF, 700 µL, 17.4 µmol) along with 15-crown-5-ether (7.66 mg, 34.8 µmol) was added to the mixture. The mixture was stirred for 15 minutes. The solvent was removed under vacuum inside the glove box to give tetra anion compound [**4a**]$^{4-}$$[Na^+]_4$ as a green solid. **^{1}H NMR** (600 MHz, THF-d_8, 300 K): δ 9.80 (4H, p

Supplementary Figures

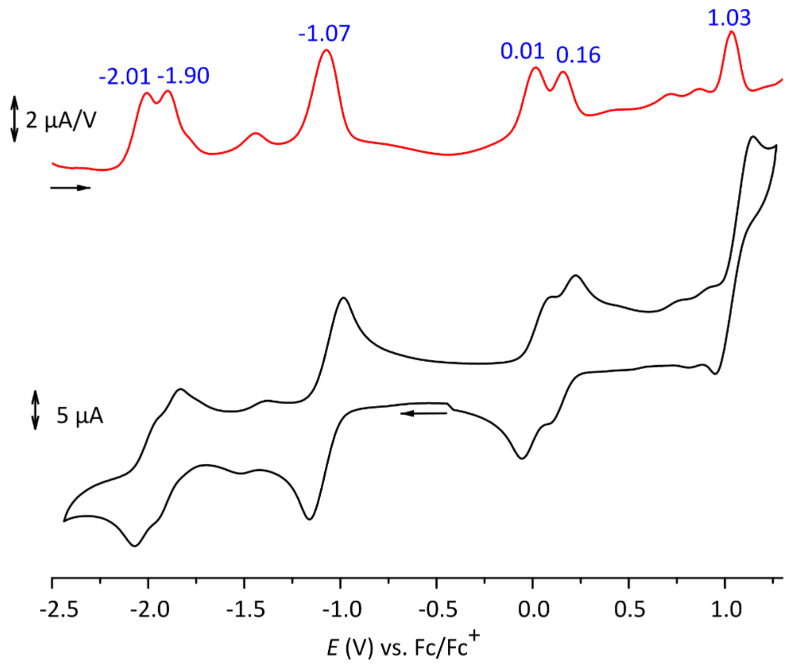

Supplementary Figure 1. Differential pulse voltammogram (red) and cyclic voltammogram (black) for compound **4a** in different potential setup (dichloromethane solvent, $[Bu_4N]PF_6$ as supporting electrolyte; glassy carbon, Pt rod, and ferrocene/ferrocenium couple as a working, counter, and reference electrode, respectively, 50 mV/s.

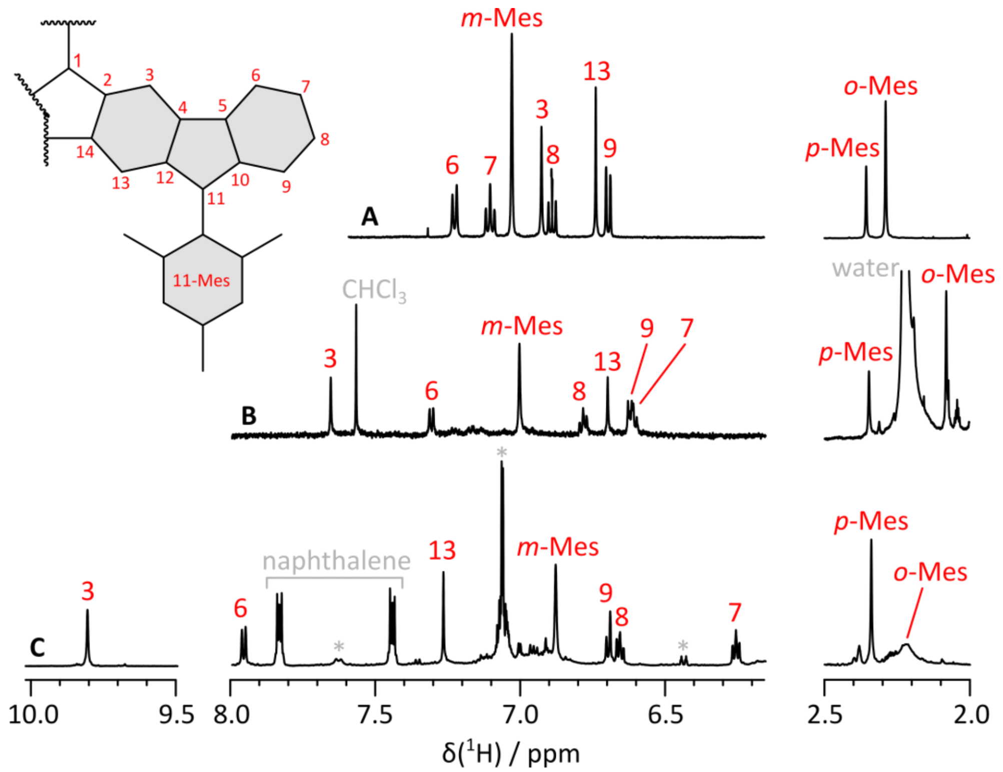

Supplementary Figure 2. ^{1}H NMR spectra of [**4a**][$SbCl_6$]$_2$ (**A**, 500 MHz, CD_2Cl_2, 300 K), in-situ generated [**4a**]$^{2–}$ (**B**, excess $CoCp_2$ used, 600 MHz, MeCN-d_3, 270 K, trace $CHCl_3$ introduced with the neutral **4a**), and in-situ generated [**4a**]$^{4–}$ (**C**, excess Na naphthalenide and 15-crown-5 used, 600 MHz, THF-d_8, 300 K; signals indicated with * are tentatively assigned to partly hydrogenated naphthalene derivatives present in the naphthalenide solution). Spectral assignments are based on 2D NMR data shown in Supplementary Fig. 4–11.

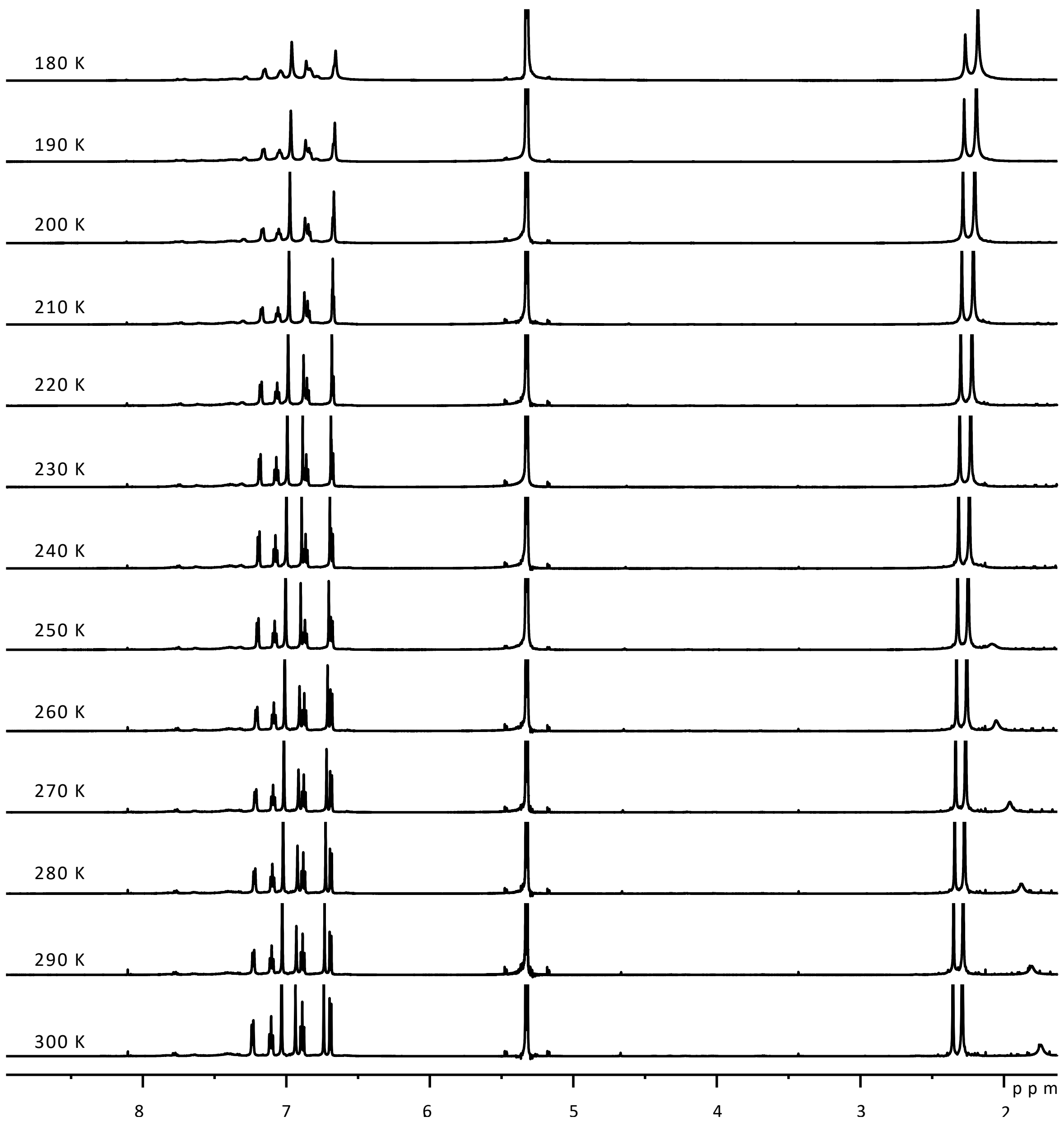
180 K
190 K
200 K
210 K
220 K
230 K
240 K
250 K
260 K
270 K
280 K
290 K
300 K
ppm
8
7
6
5
4
3
2

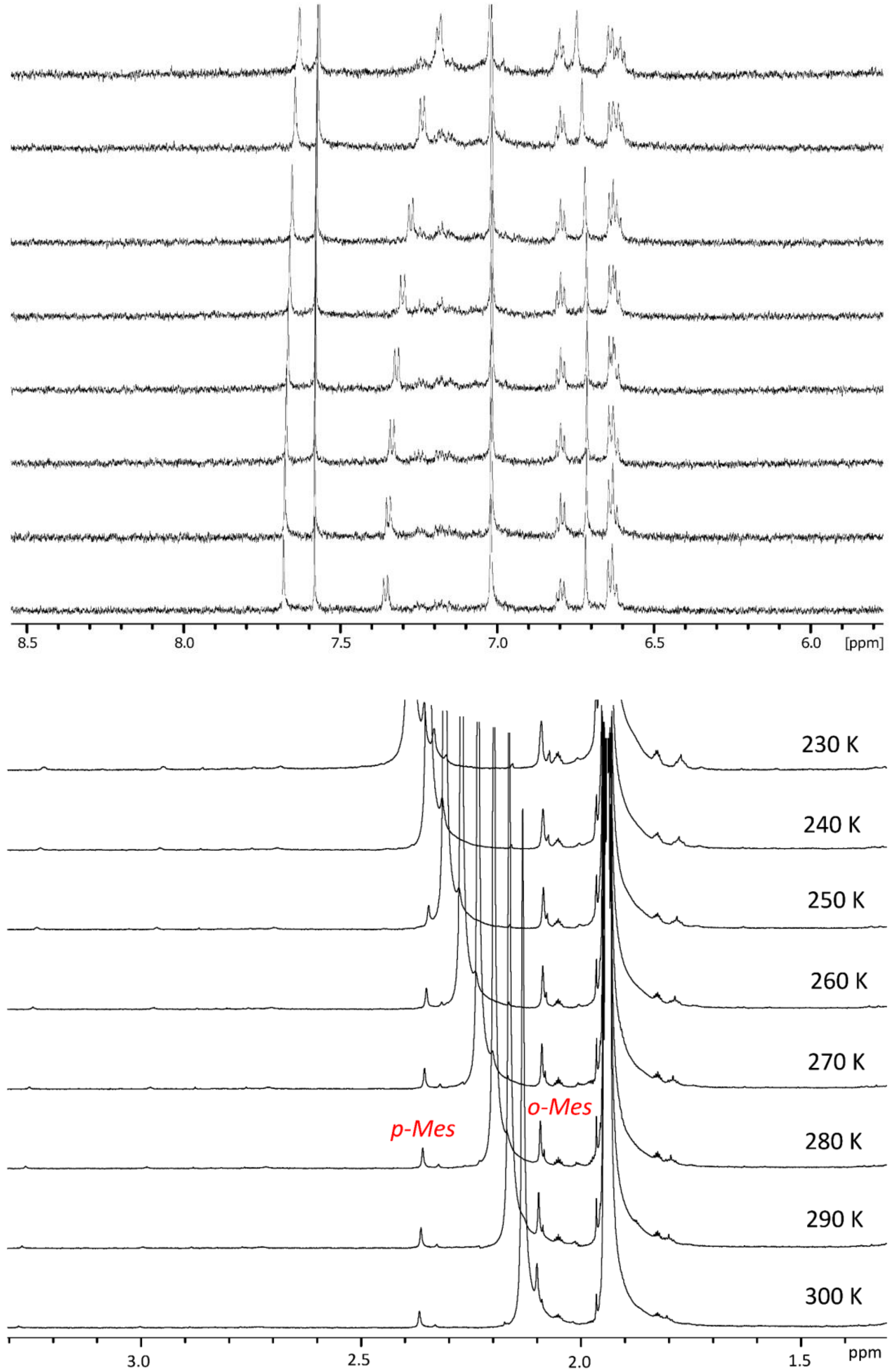

Supplementary Figure 3. Variable temperature ^{1}H NMR spectra of compounds [**4a**]$^{2+}$[$SbCl_6^-$]$_2$ (600 MHz, dichloromethane-d_2, 300-180 K, previous page) and [**4a**]$^{2-}$[$CoCp_2^+$]$_2$ (600 MHz, acetonitrile-d_3, 300-230 K, this page).

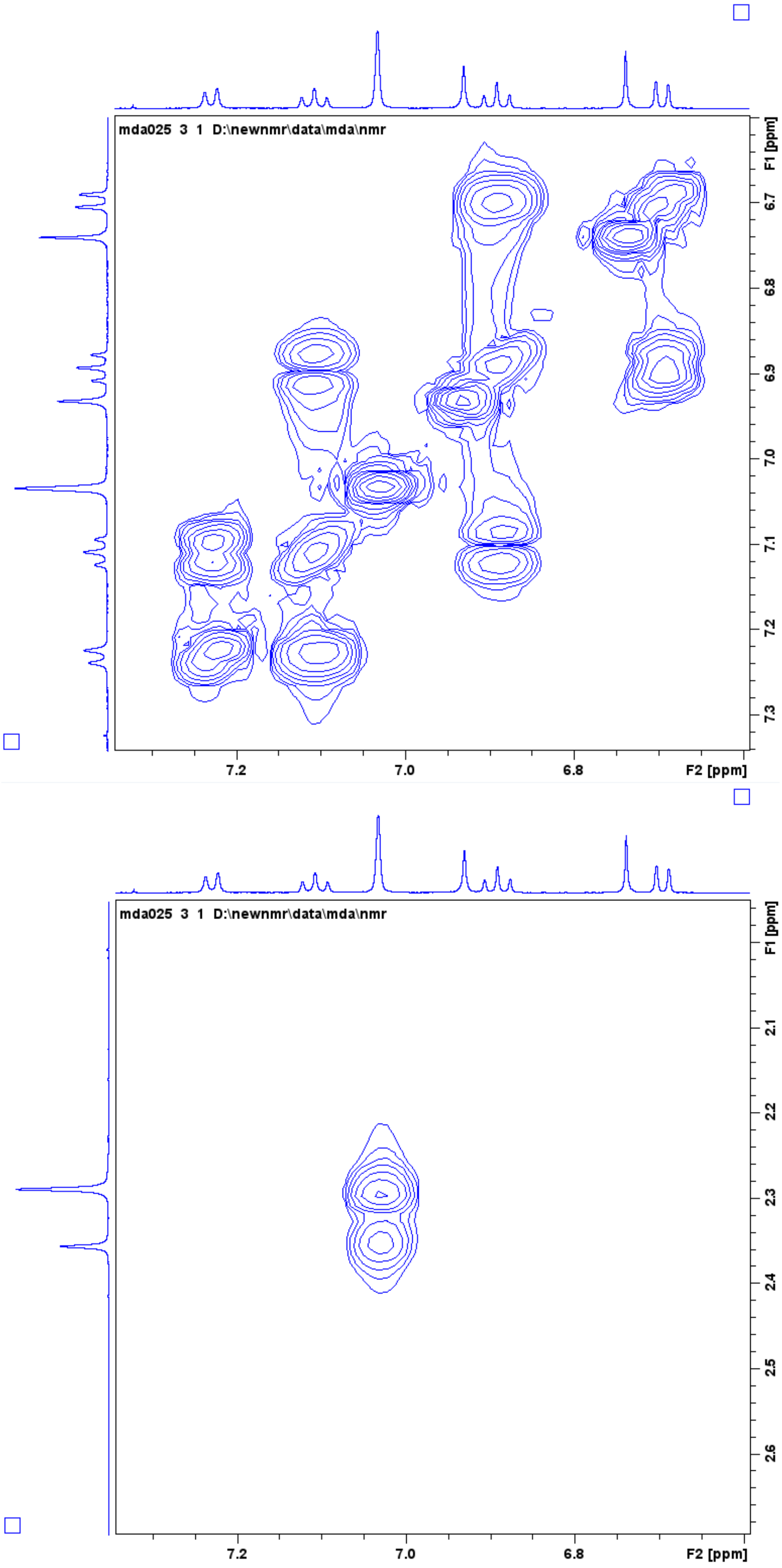

Supplementary Figure 4. Partial COSY spectrum of compound [**4a**]$^{2+}$[$SbCl_6^-$]$_2$ (600 MHz, dichloromethane-d_2, 300 K).

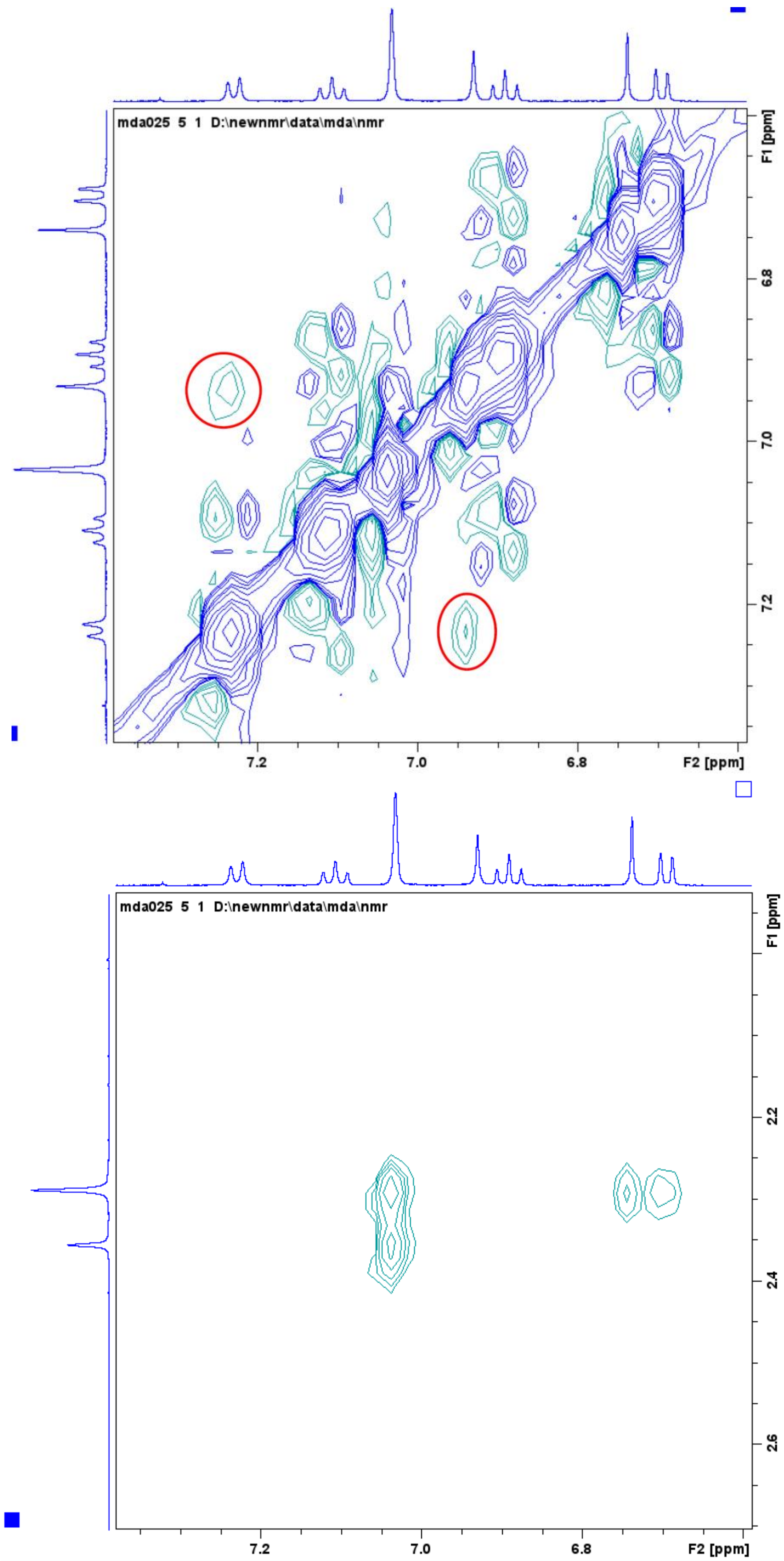

Supplementary Figure 5. Partial ROESY spectrum of compound [**4a**]$^{2+}$[$SbCl_6^-$]$_2$ (600 MHz, dichloromethane-d_2, 300 K).

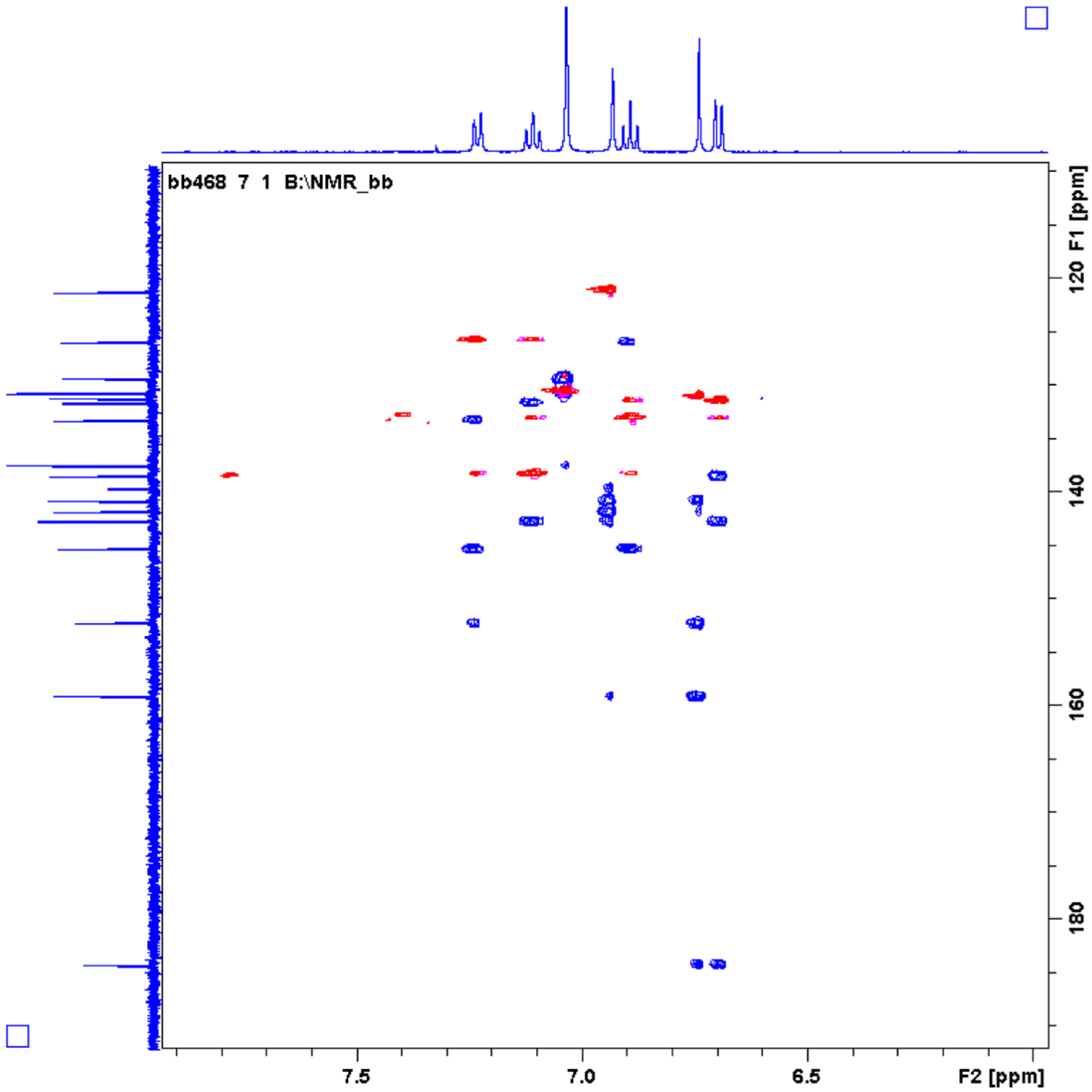

Supplementary Figure 6. Overlaid selected region of HSQC (red) and HMBC (blue) correlation spectra of Compound [**4a**]$^{2+}$[$SbCl_6^-$]$_2$ (600 MHz, dichloromethane-d_2, 300 K).

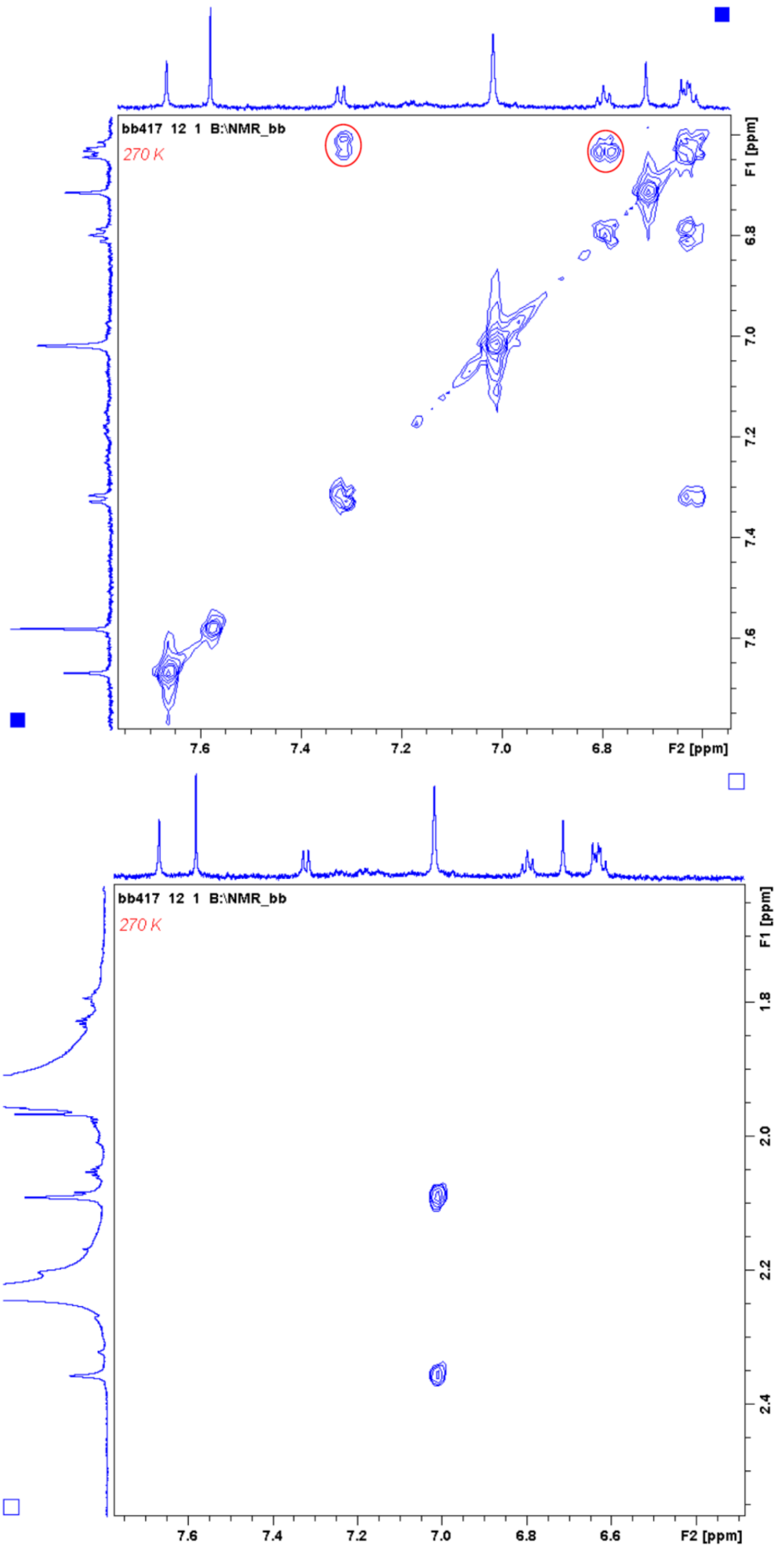

Supplementary Figure 7. Partial COSY spectrum of [**4a**]$^{2-}$[$CoCp_2^+$]$_2$(600 MHz, acetonitrile-*d*$_3$, 270 K).

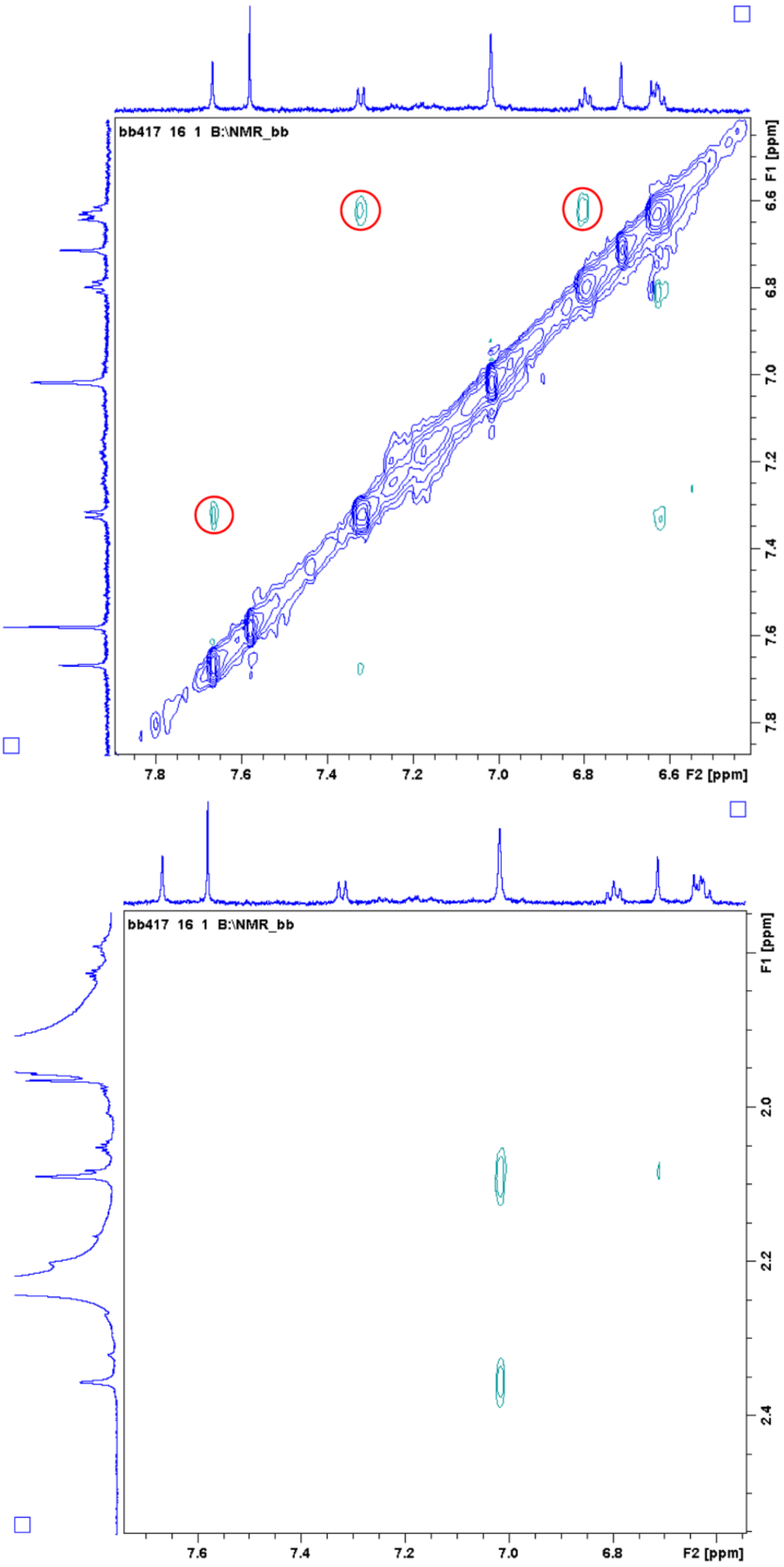

Supplementary Figure 8. Partial ROESY spectrum of [**4a**]$^{2-}$[$CoCp_2^+$]$_2$(600 MHz, acetonitrile-d_3, 270 K).

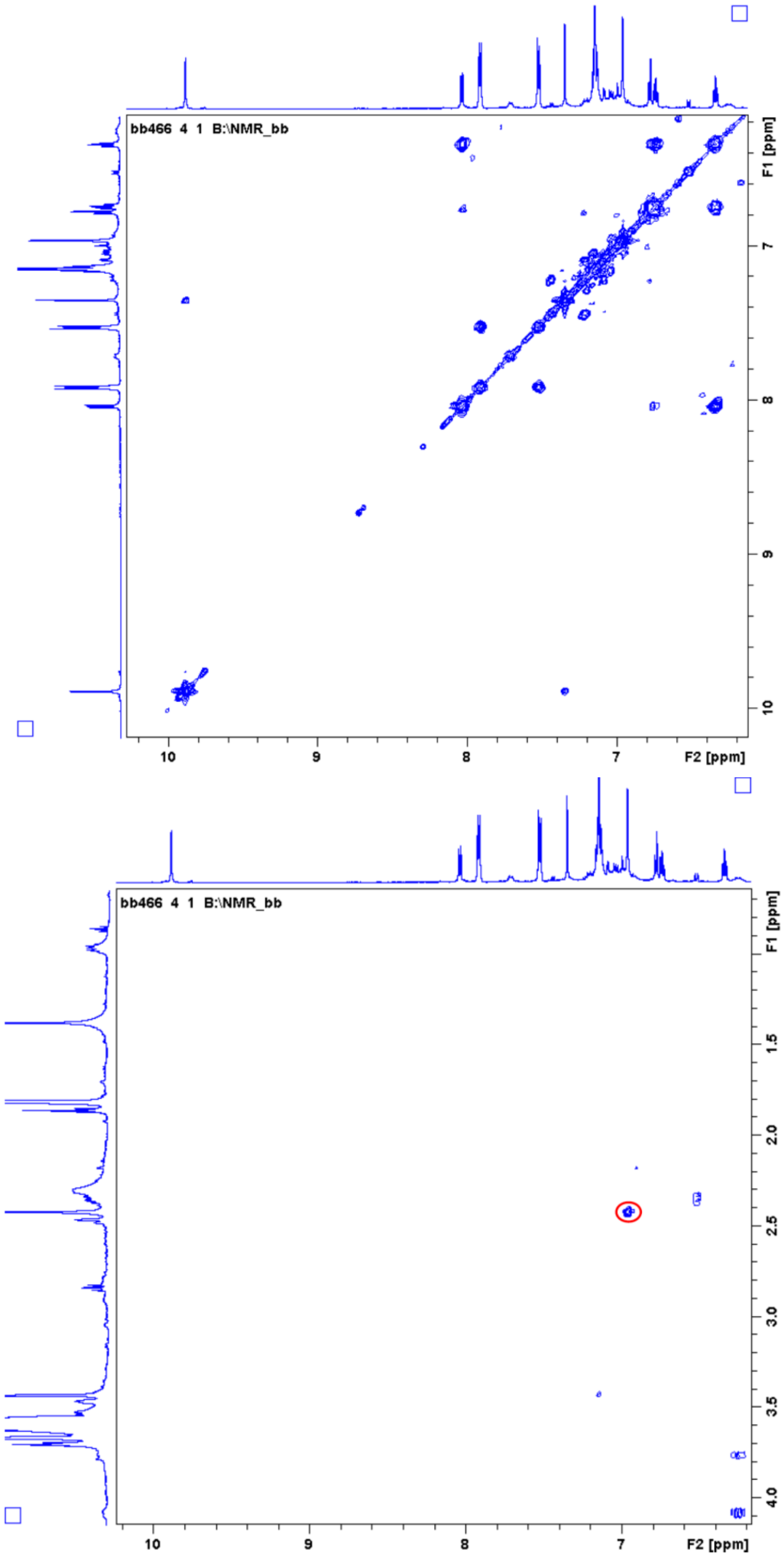

Supplementary Figure 9. Partial COSY spectrum of [**4a**]$^{4-}$ (600 MHz, Tetrahyrofuran-d_8, 300 K).

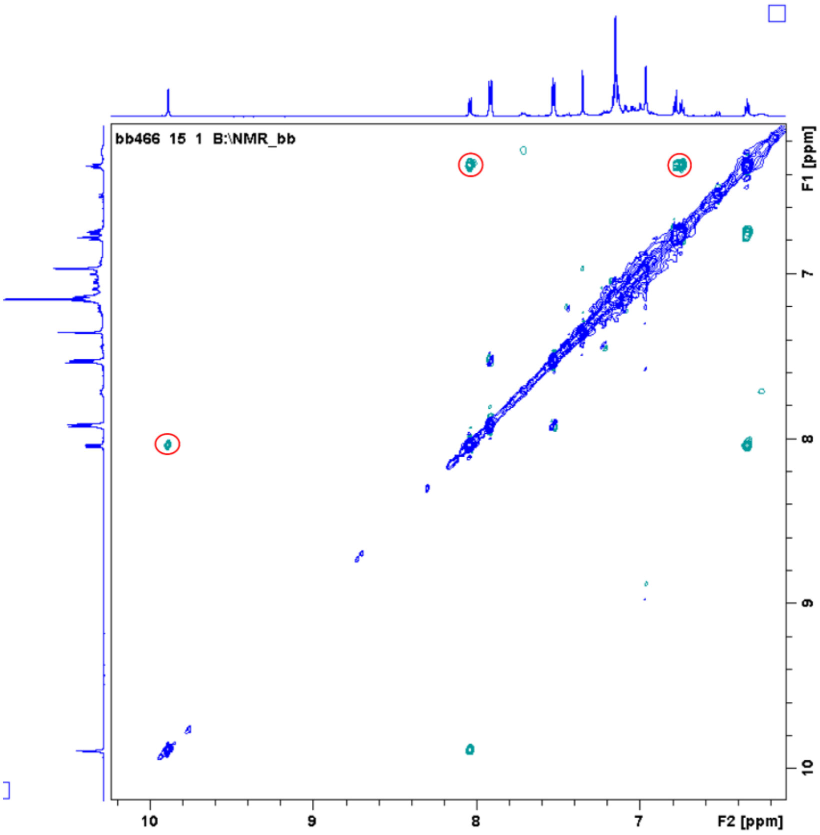

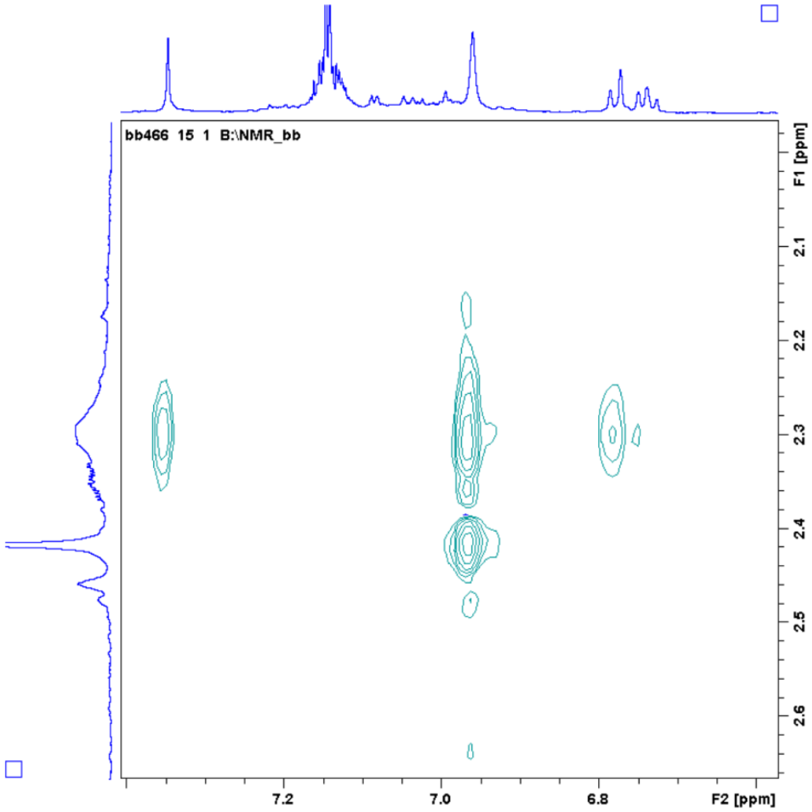

Supplementary Figure 10. Partial ROESY spectrum of [**4a**]$^{4-}$ (600 MHz, Tetrahyrofuran-d_8, 300 K).

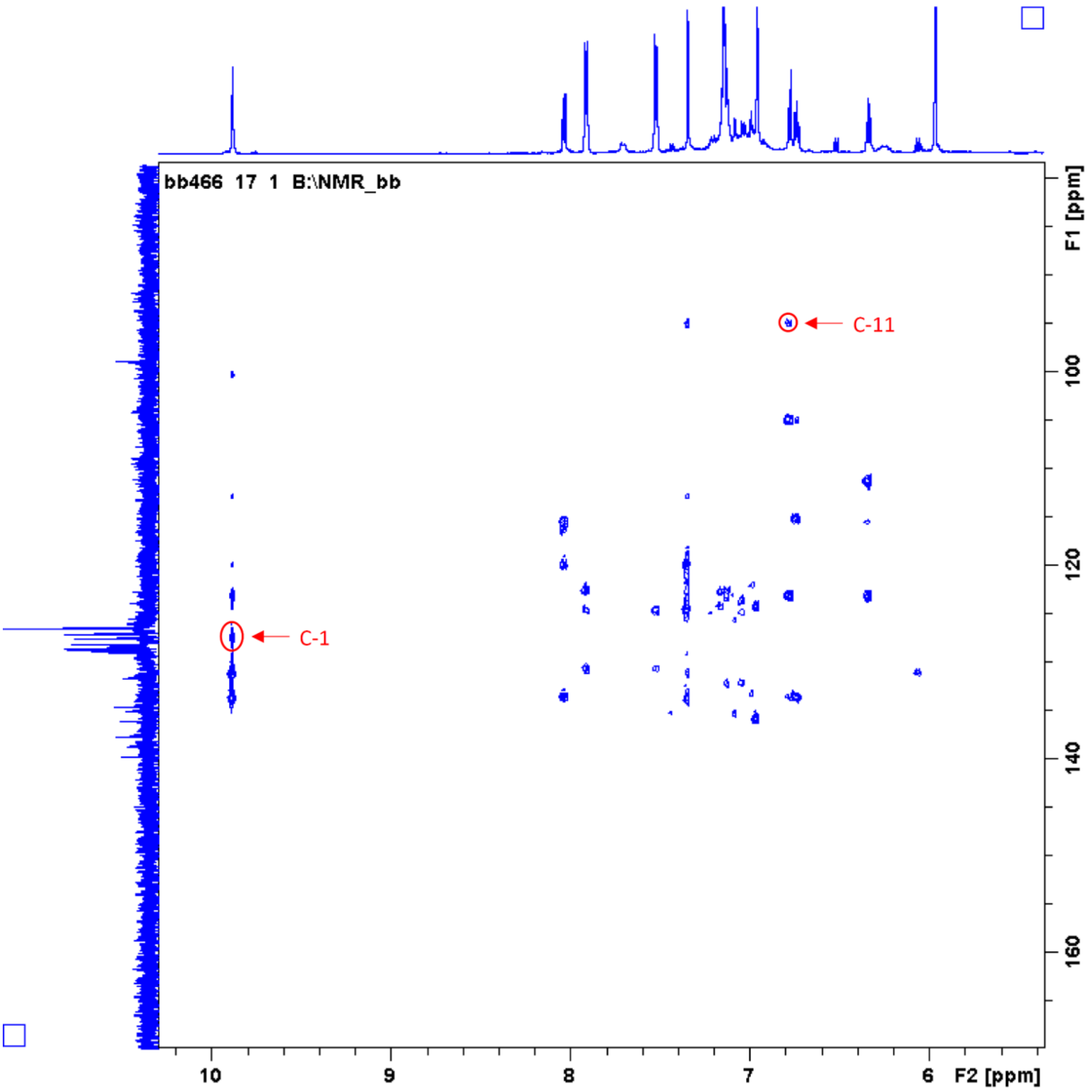

Supplementary Figure 11. Partial ^{1}H–^{13}C HMBC spectrum of [**4a**]$^{4–}$ (600 MHz, Tetrahyrofuran-d_8, 300 K). The positions of carbons C-11 (94.9 ppm) and C-1 (127.0) are indicated in the Figure.

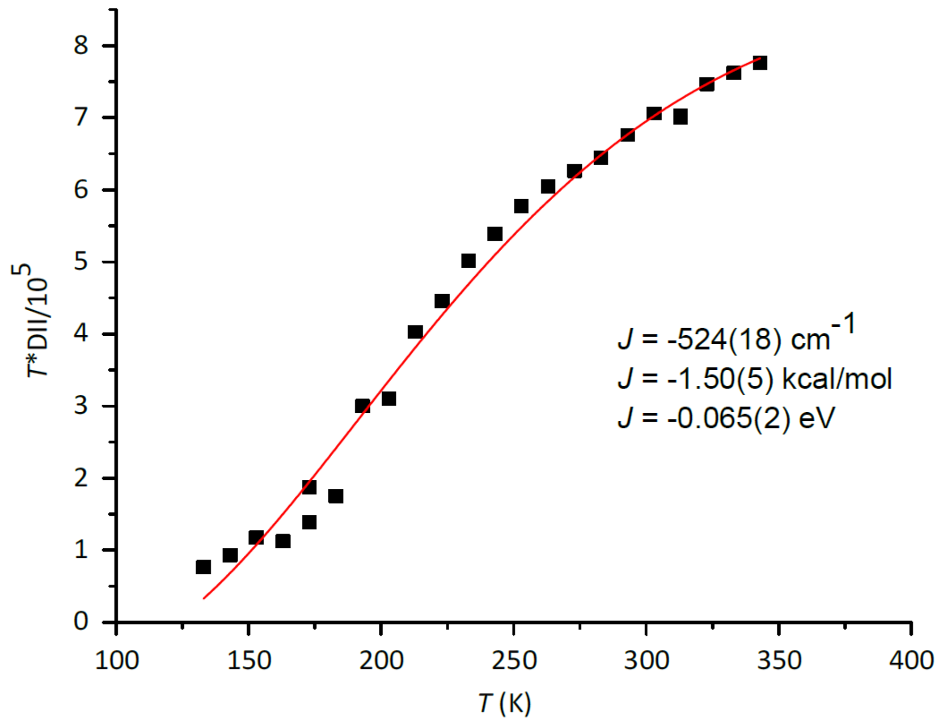

Supplementary Figure 12. Temperature dependence of the product of temperature and doubly integrated ESR signal intensity obtained from temperature-dependent ESR spectra of **4a**. The red line represents the best fit of data points to the Bleaney-Bowers equation with exchange integral –1.50 kcal/mol.

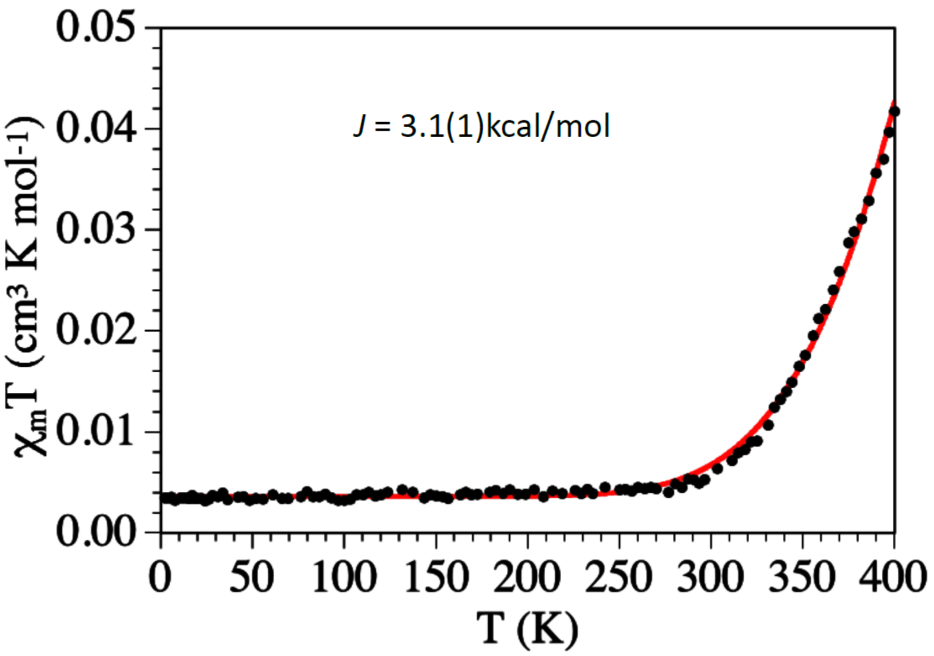

Supplementary Figure 13. Thermal variation of the $\chi_m T$ product obtained for solid-state sample of compound **4a**, using SQUID measurements (5 to 400 K temperature range). The data were fitted with the Bleaney–Bowers model (fixed g, S= 1/2 impurity content of 1.0 %).

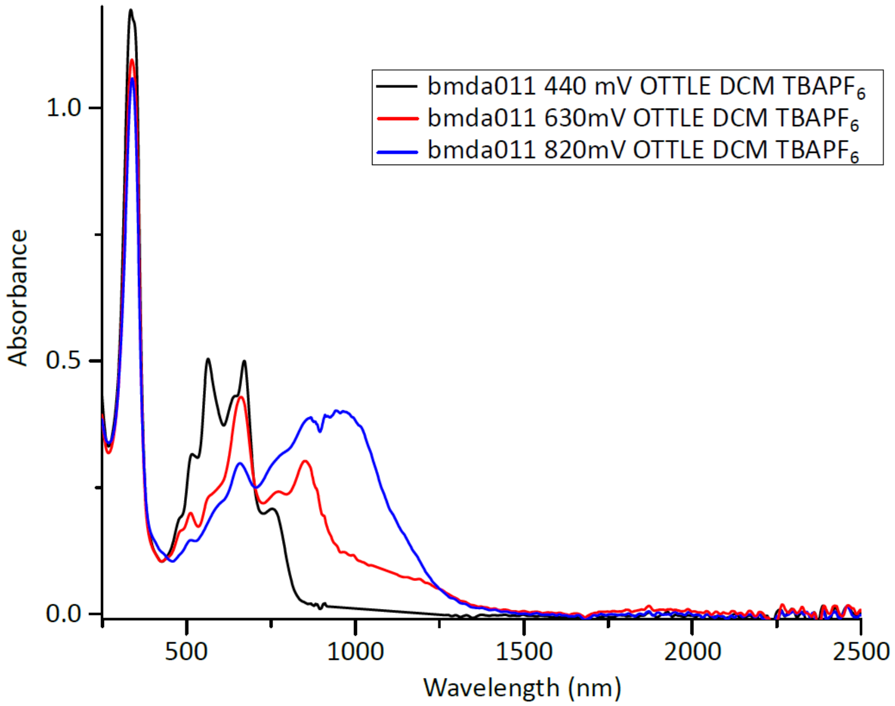

Supplementary Figure 14. OTTLE measurefor compound **4a** in different potential setup (dichloromethane solvent, $[Bu_4N]PF_6$ as supporting electrolyte; glassy carbon, Pt rod, and Ag/AgCl couple as a working, counter, and reference electrode, respectively, 40 mV/s.

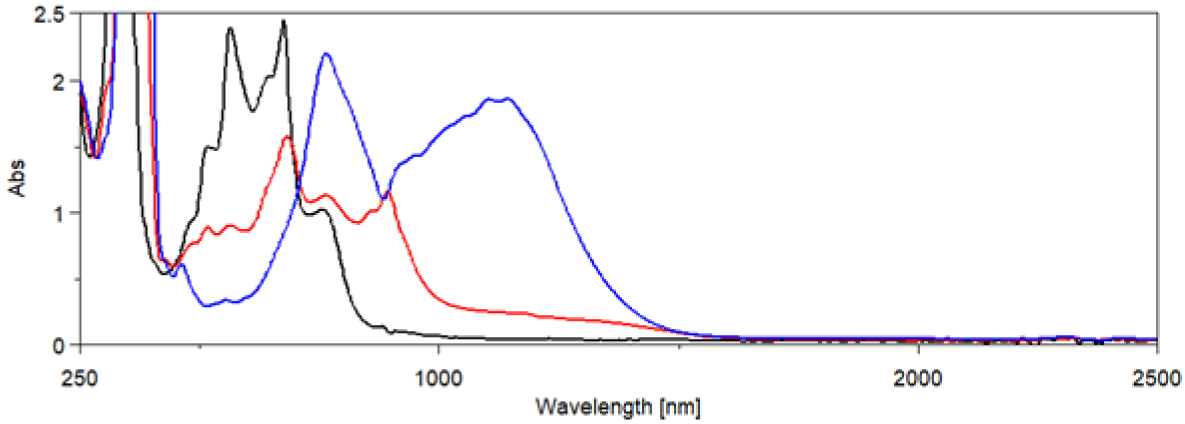

Supplementary Figure 15. OTTLE measurement for compound **4a** in different potential setup (dichloromethane solvent, $[Bu_4N]PF_6$ as supporting electrolyte; glassy carbon, Pt rod, and Ag/AgCl couple as a working, counter, and reference electrode, respectively, 40 mV/s.

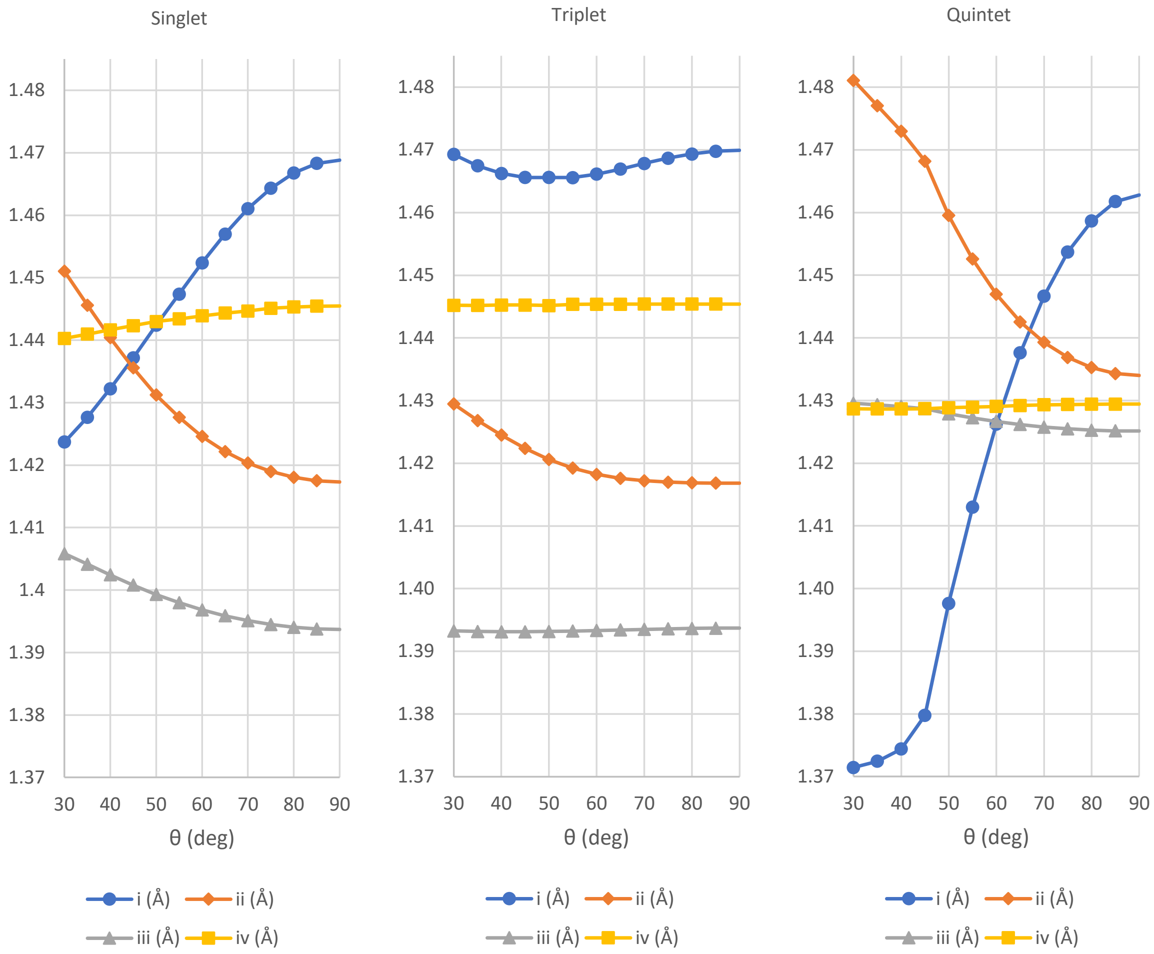

Supplementary Figure 16. Variation of bond lengths in the CAM geometries of ^{m}[**4b**] (*m* = 1, 3, 5) as a function of torsion angle θ (in degrees).

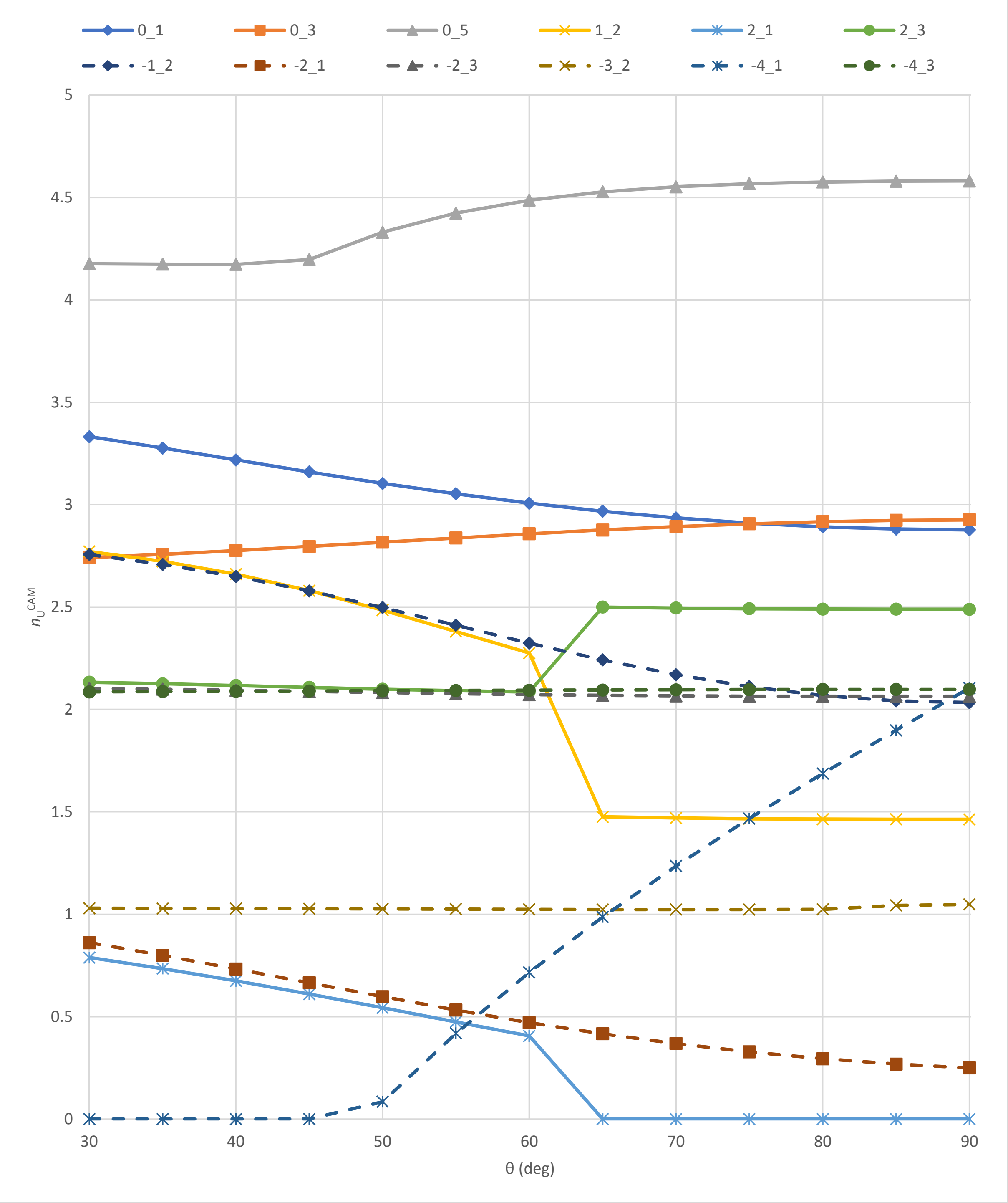

Supplementary Figure 17. Variation of the number of unpaired electrons n_U^{CAM} as a function of the torsion angle θ twist angle in the relaxed PES scans performed for ^{m}[**4b**]^n (labeled *n_m* in the legend).

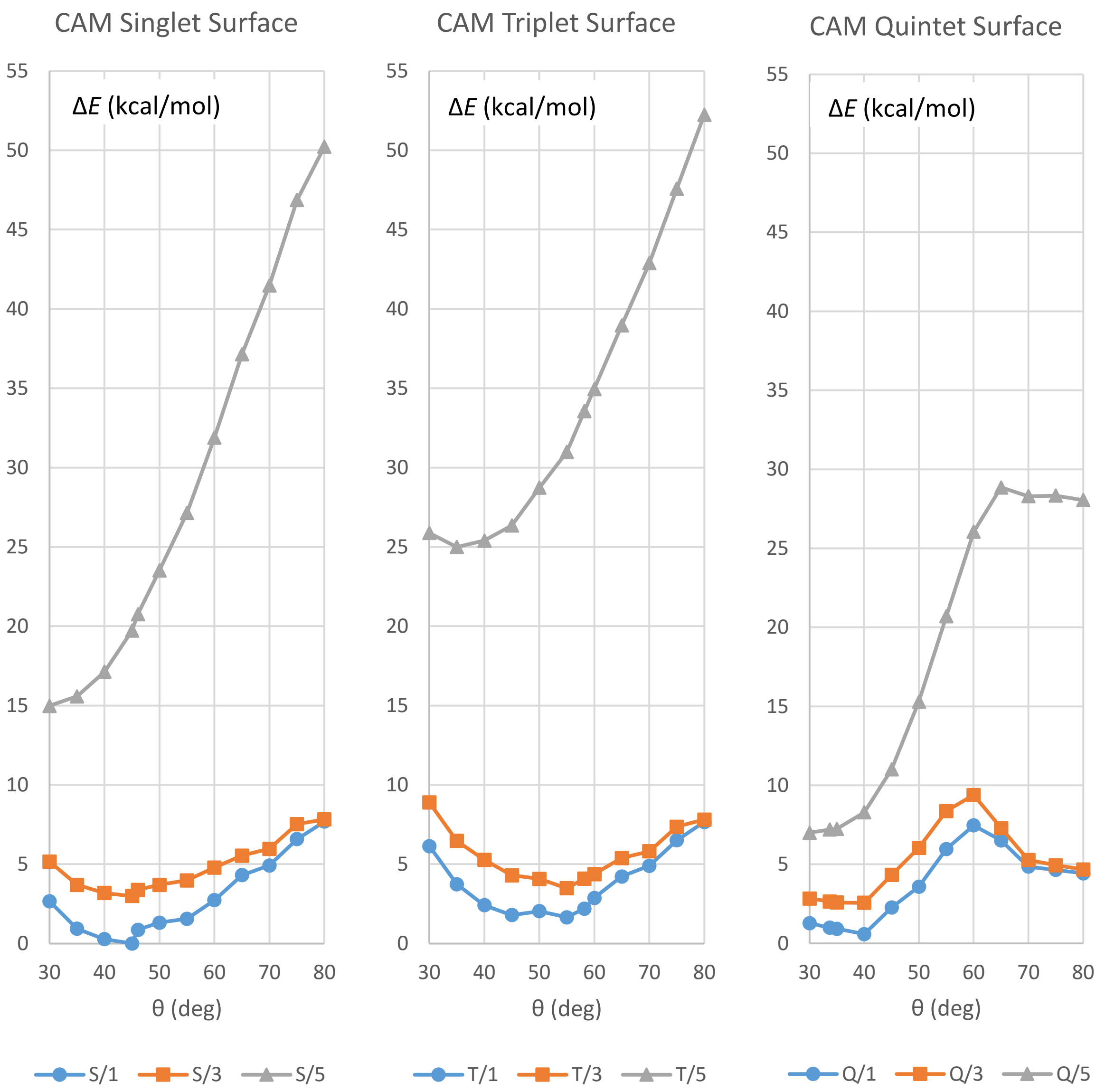

Supplementary Figure 18. Energies of the lowest singlet (X/1), triplet (X/3) and quintet (X/5) states calculated using RAS(4,4)-SF-*sr*B3LYP/cc-pVDZ for CAM relaxed potential energy scans performed for the singlet, triplet, and quintet configurations (X = S, T, Q, respectively, θ coordinate in degrees). Energies are given in kcal/mol relative to the lowest singlet energy obtained in these calculations.

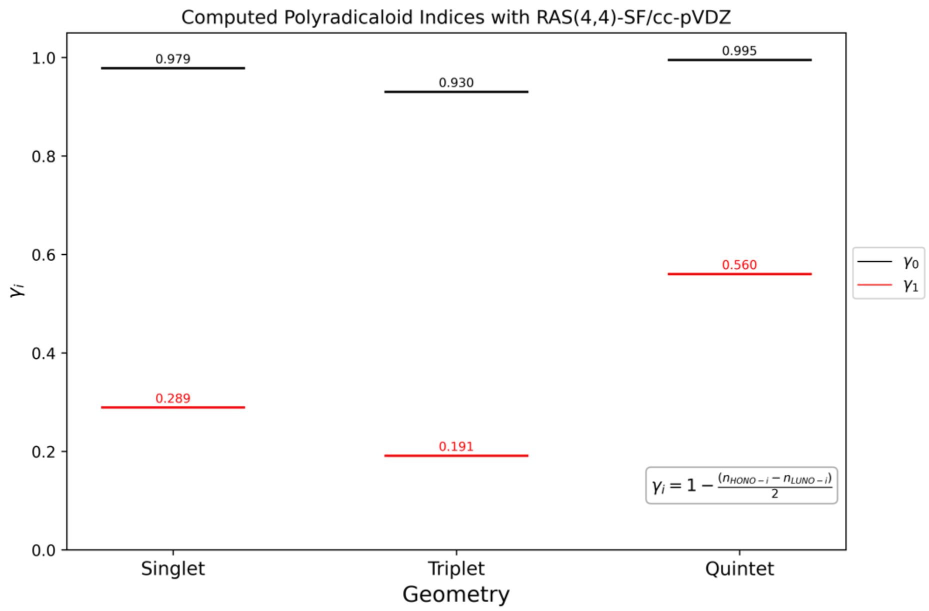

Supplementary Figure 19. RAS(4,4)-SF-*sr*B3LYP/cc-pVDZ polyradicaloid indices of the lowest singlet state calculated for CAM energy minima obtained for [1][**4b**] (singlet), [3][**4b**] (triplet), [5][**4b**] (quintet).

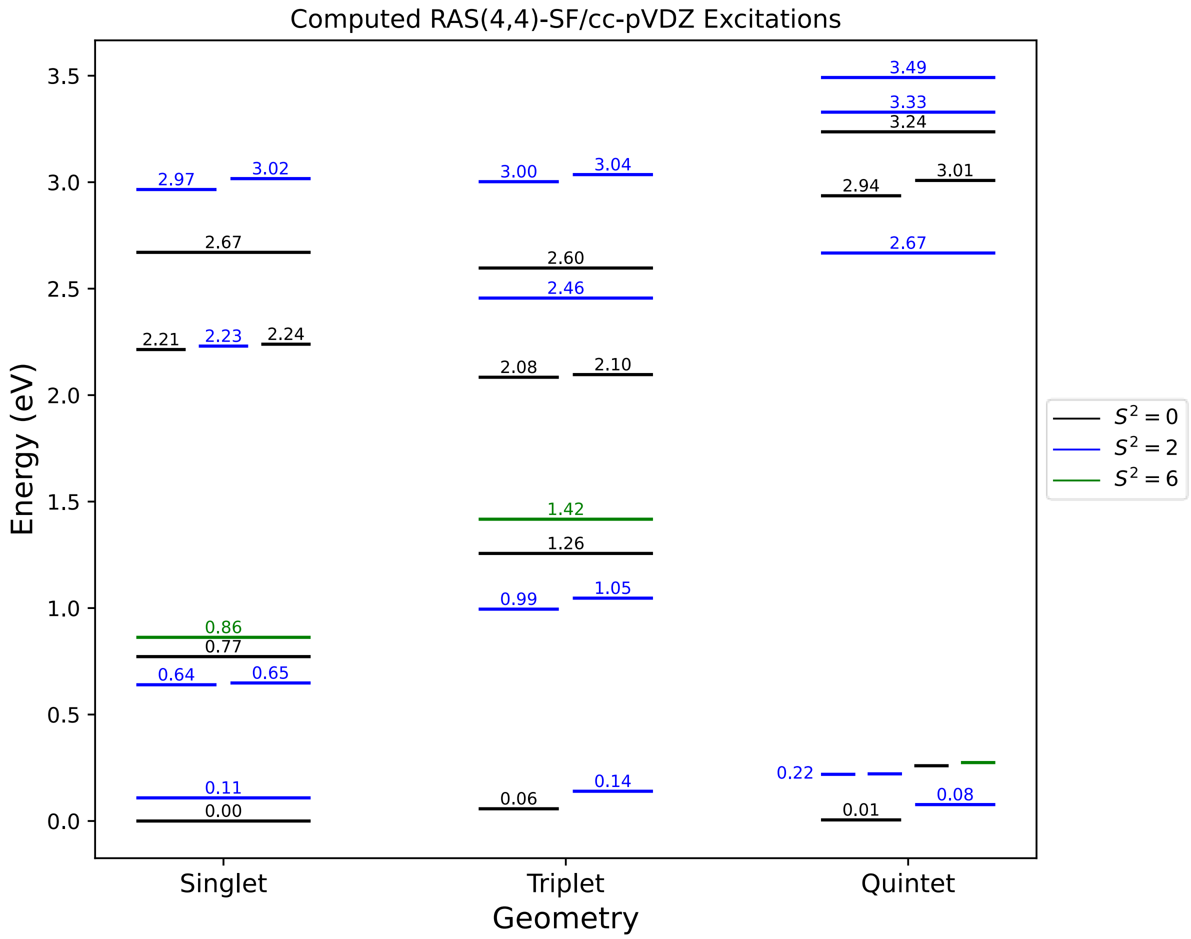

Supplementary Figure 20. RAS(4,4)-SF-*sr*B3LYP/cc-pVDZ energies (relative to the lowest singlet state) calculated for CAM energy minima obtained for [1][**4b**] (singlet), [3][**4b**] (triplet), [5][**4b**] (quintet).

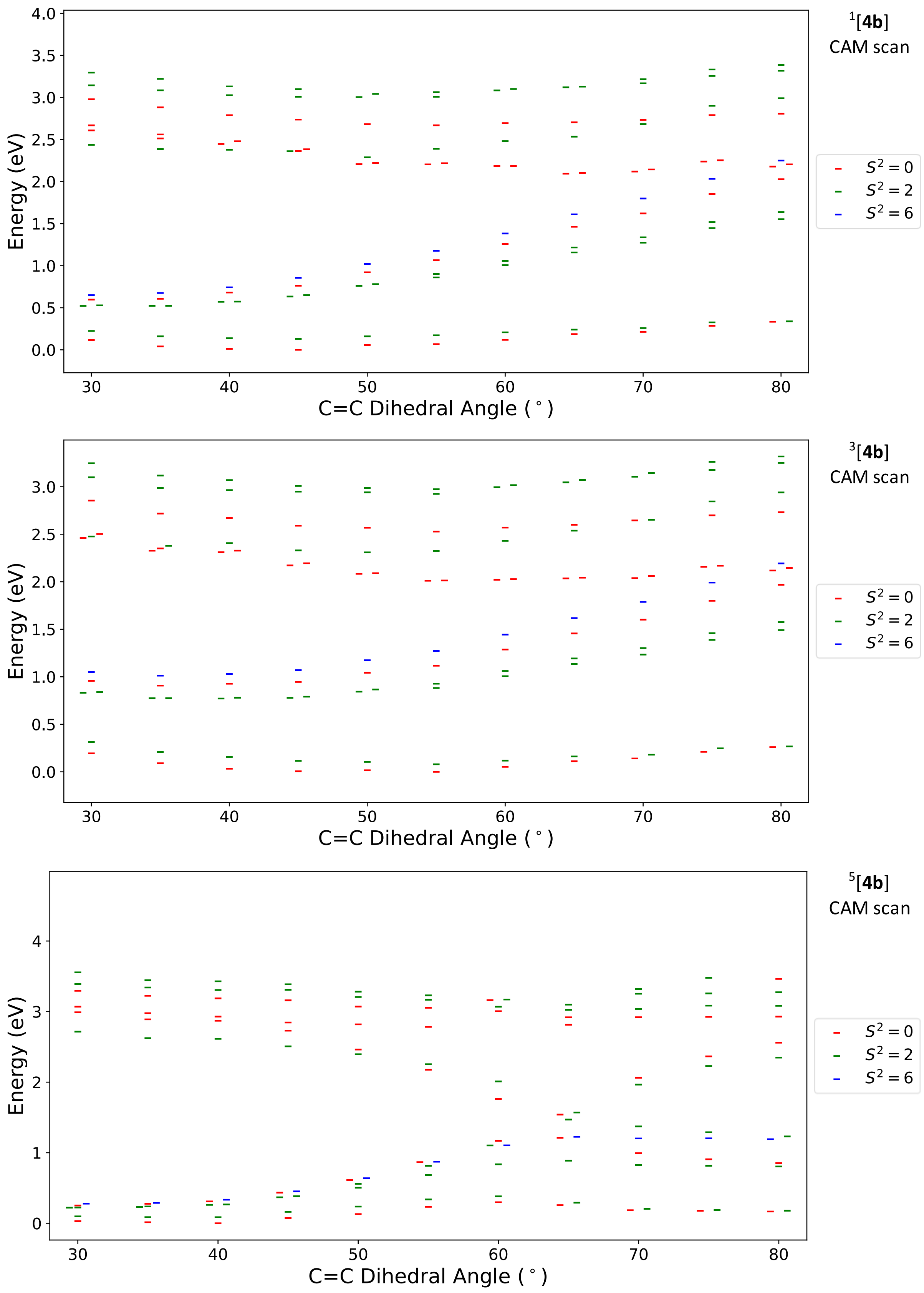

Supplementary Figure 21. RAS(4,4)-SF-*sr*B3LYP/cc-pVDZ energies (relative to the lowest singlet state) calculated for CAM geometries obtained for the relaxed PES scan performed for [1][**4b**] (top), [3][**4b**] (center), [5][**4b**] (bottom). The quintet RO-B3LYP reference breaks down for θ > 80° and the corresponding values were not included in the plots.

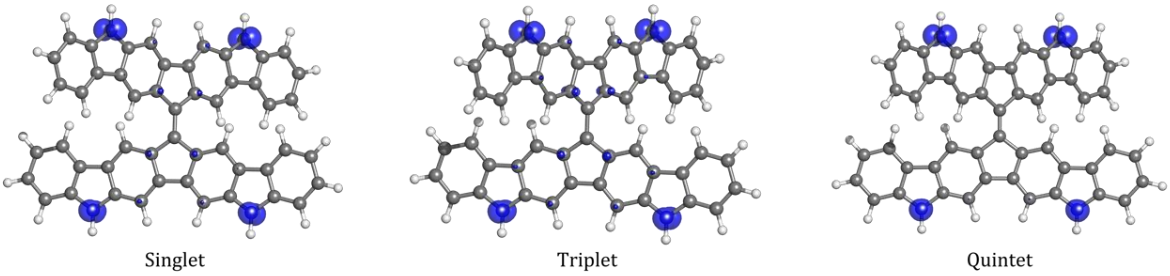

Supplementary Figure 22. RAS(4,4)-SF-*sr*B3LYP/cc-pVDZ odd electron densities for the lowest energy singlet of the neutral **4b** calculated for CAM energy minima obtained for the singlet, triplet, and quintet hypersurface. Isosurface values are set to the odd electron count / 350.

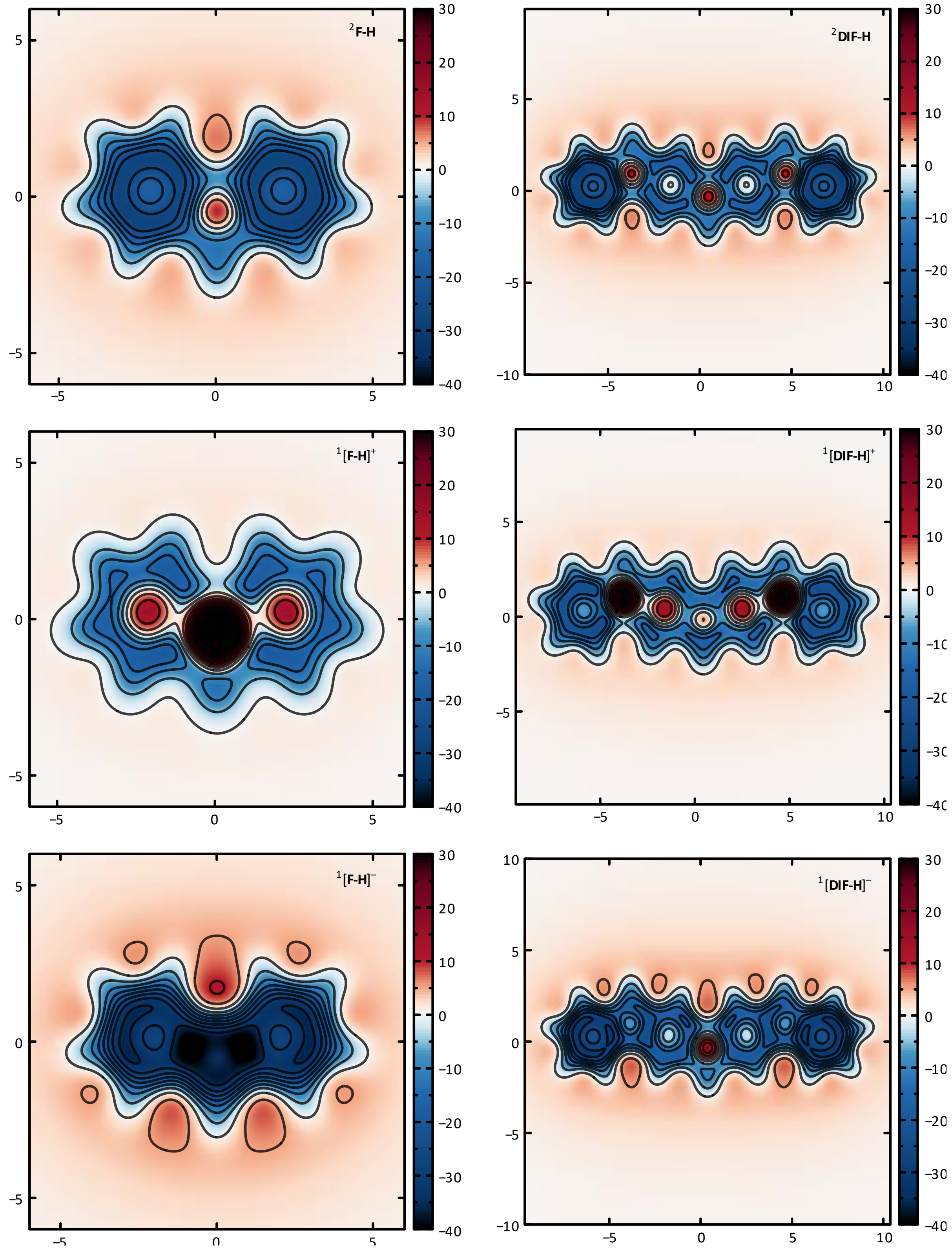

Supplementary Figure 23. 2D NICS scans performed for selected oxidation levels and multiplicities of fluorenyl (**F-H**, left) and diindenofluorenyl (**DIF-H**, right). Chemical shifts and spatial coordinates are given in ppm and Ångstroms, respectively. The cross-sectional plane (CSP) was located 1 Å above the molecular plane. NMR shieldings were evaluated along the normal of the CSP using the CAM level of theory.

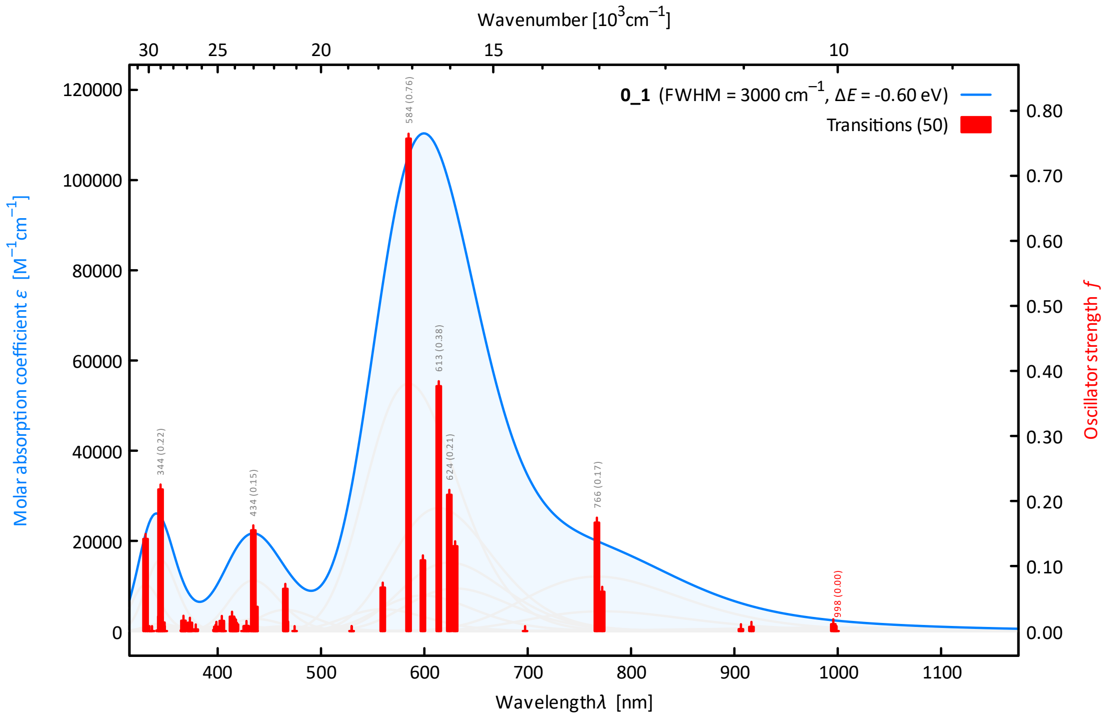

Supplementary Figure 24. [1]**[4b]**, the level of theory is UCAM-B3LYP/6-31G(d,p). All transitions have been shifted by 0.6 eV toward lower energies.

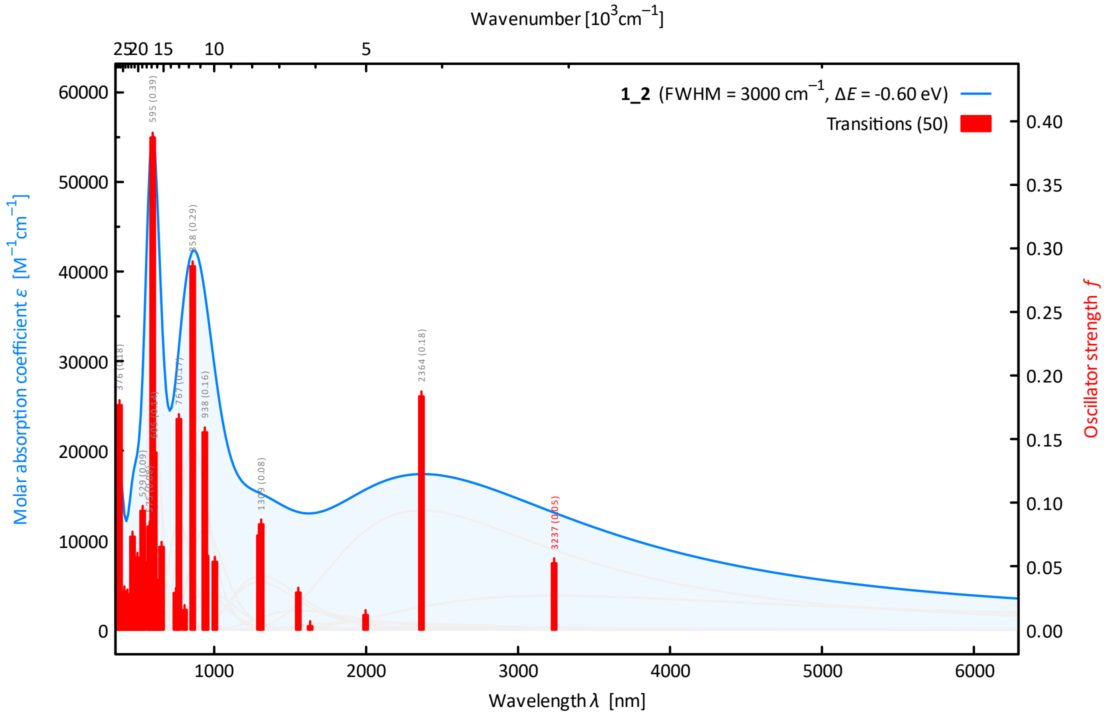

Supplementary Figure 25. [2]**[4b]**$^{1+}$, the level of theory is UCAM-B3LYP/6-31G(d,p). All transitions have been shifted by 0.6 eV toward lower energies.

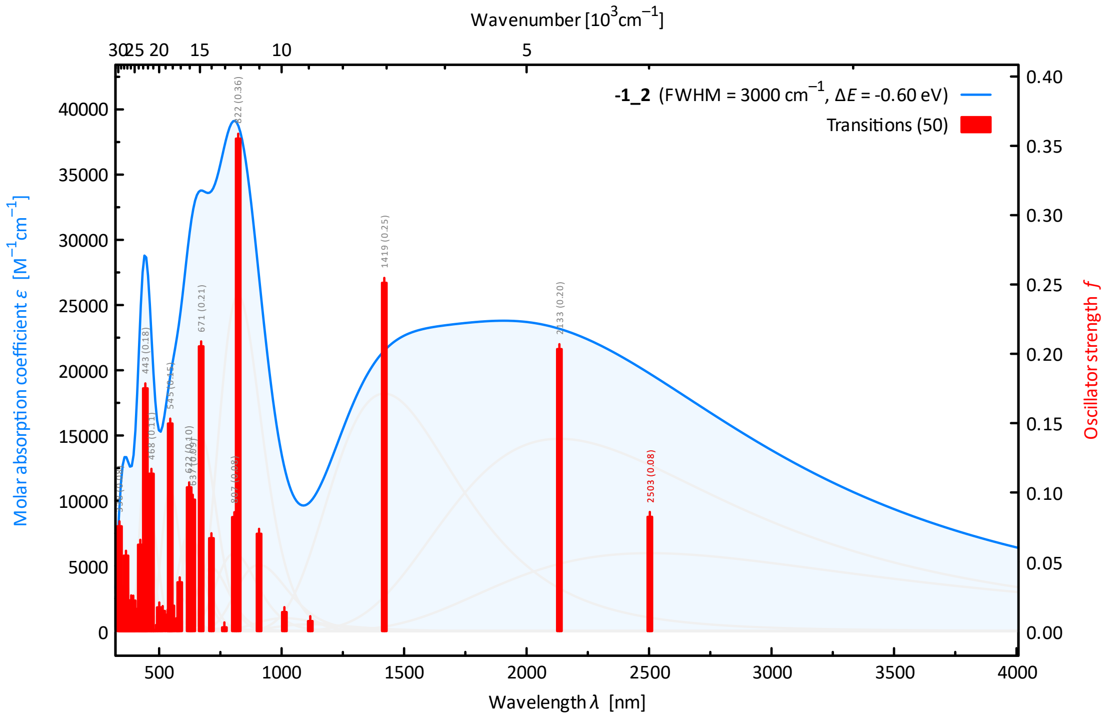

Supplementary Figure 26. [2]**[4b]**$^{1-}$, the level of theory is UCAM-B3LYP/6-31G(d,p). All transitions have been shifted by 0.6 eV toward lower energies.

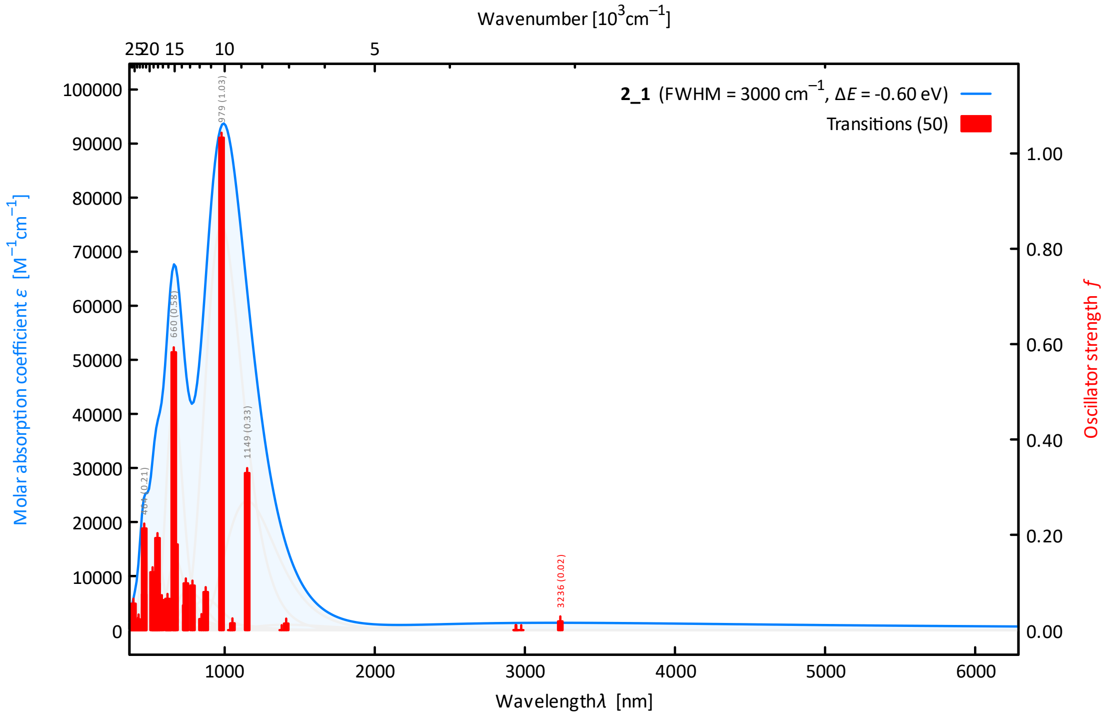

Supplementary Figure 27. [1]**[4b]**$^{2+}$, the level of theory is UCAM-B3LYP/6-31G(d,p). All transitions have been shifted by 0.6 eV toward lower energies.

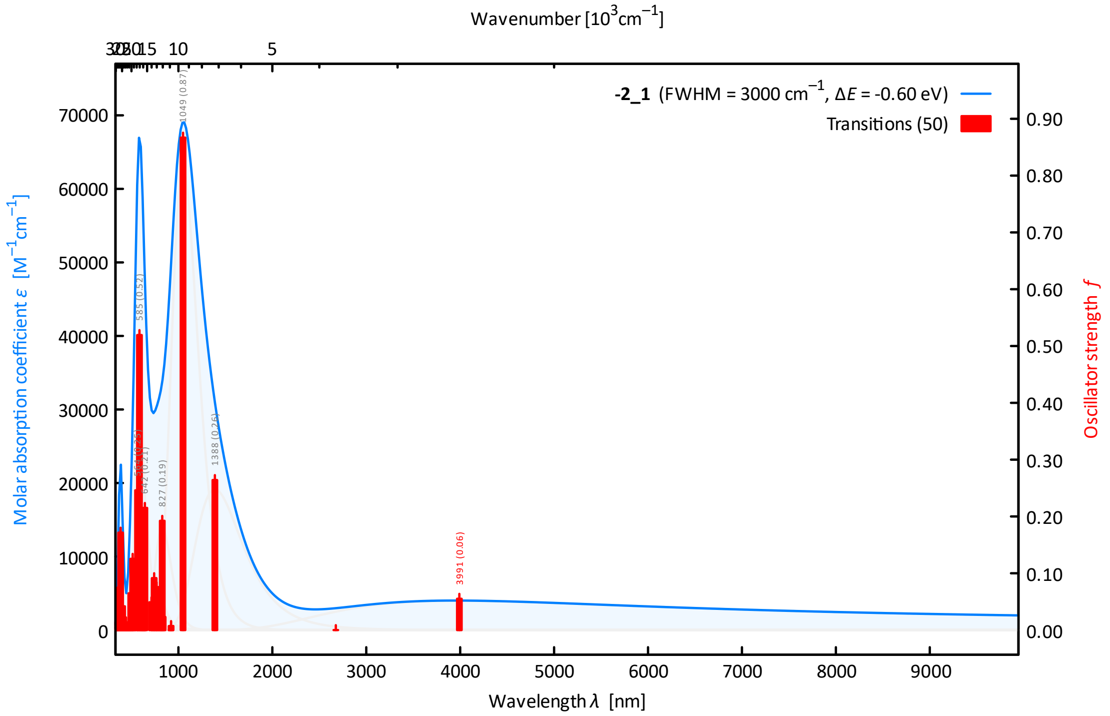

Supplementary Figure 28. [1]**[4b]**$^{2-}$, the level of theory is UCAM-B3LYP/6-31G(d,p). All transitions have been shifted by 0.6 eV toward lower energies.

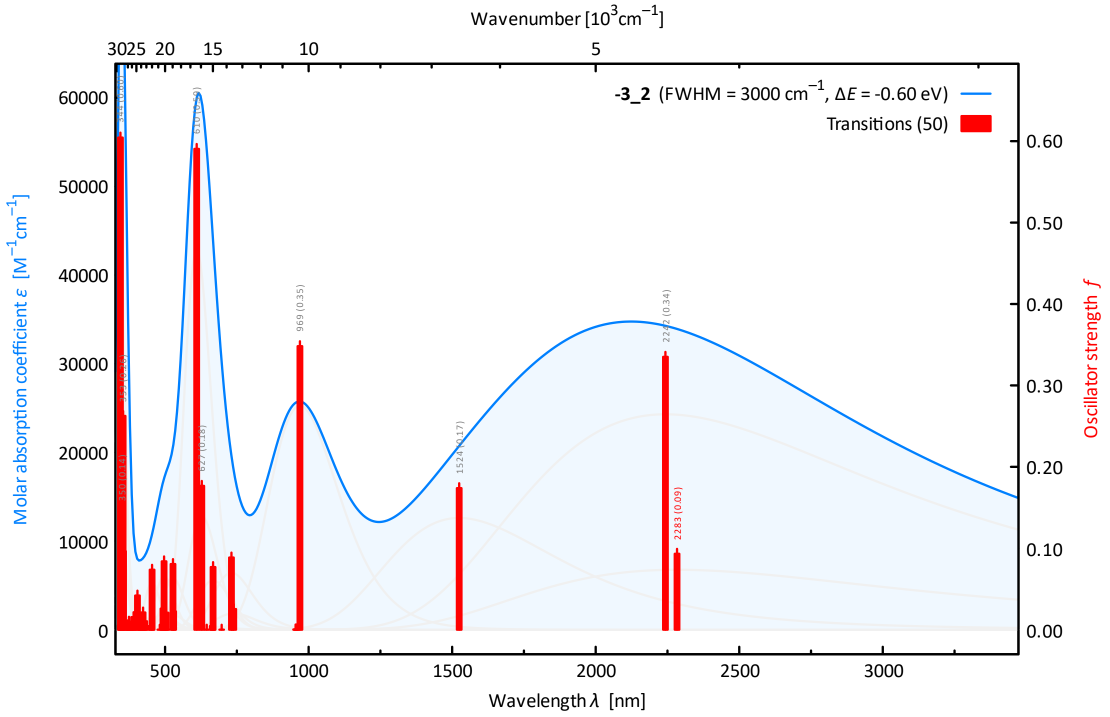

Supplementary Figure 29. [2]**[4b]**$^{3-}$, the level of theory is UCAM-B3LYP/6-31G(d,p). All transitions have been shifted by 0.6 eV toward lower energies.

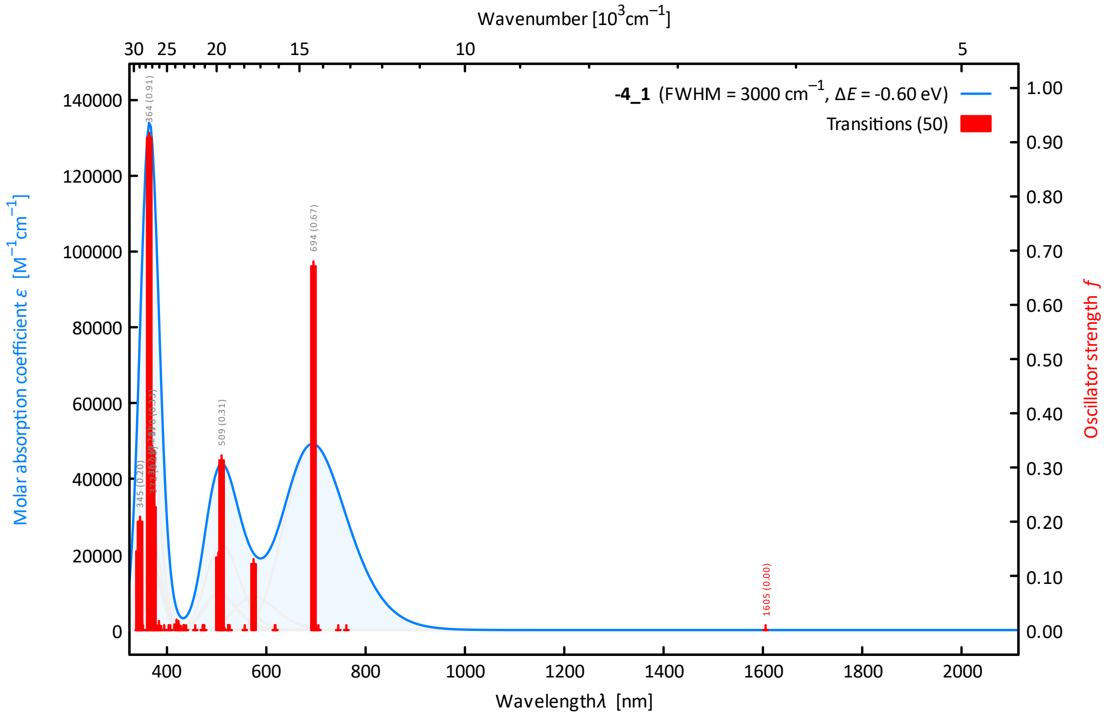

Supplementary Figure 30. ^{1}**[4b]**$^{4–}$, the level of theory is UCAM-B3LYP/6-31G(d,p). All transitions have been shifted by 0.6 eV toward lower energies.

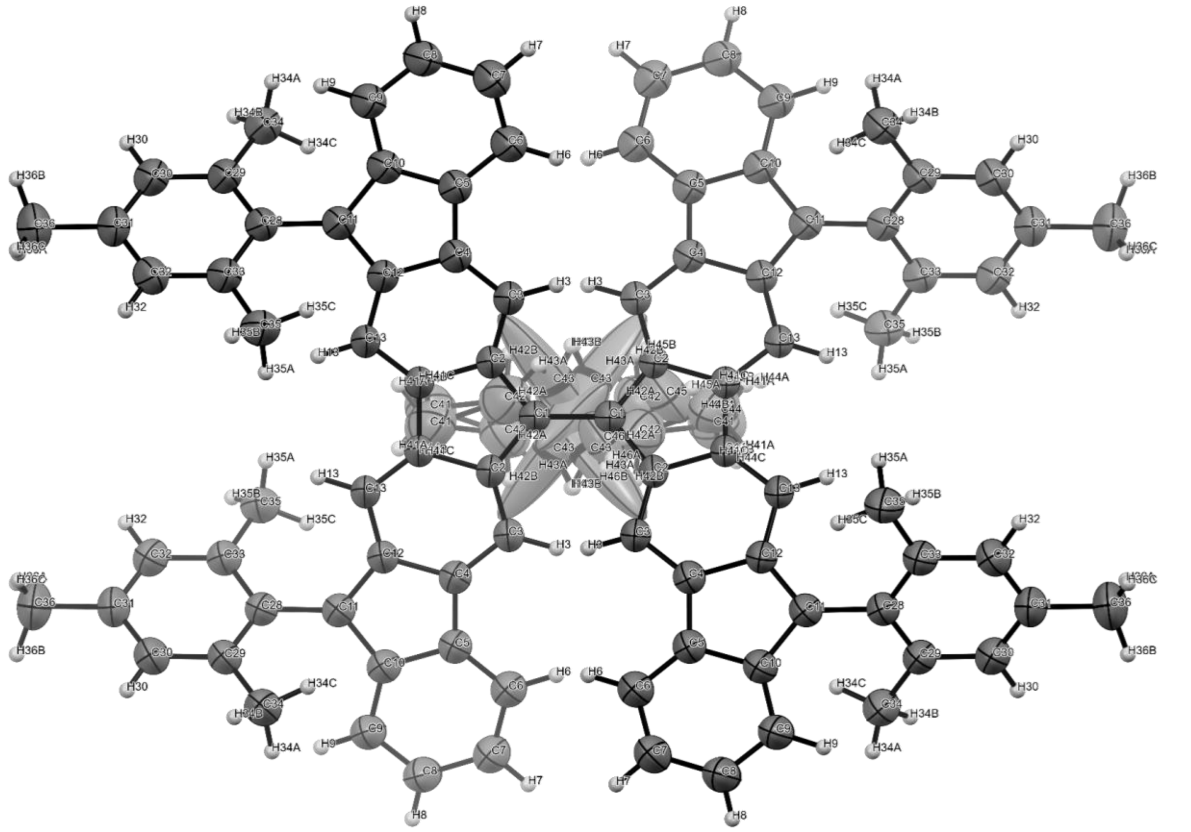

Supplementary Figure 31. ORTEP plot for **4a**·C_6H_{14} (CCDC 2250914). Thermal ellipsoids are at the 50% probability level.

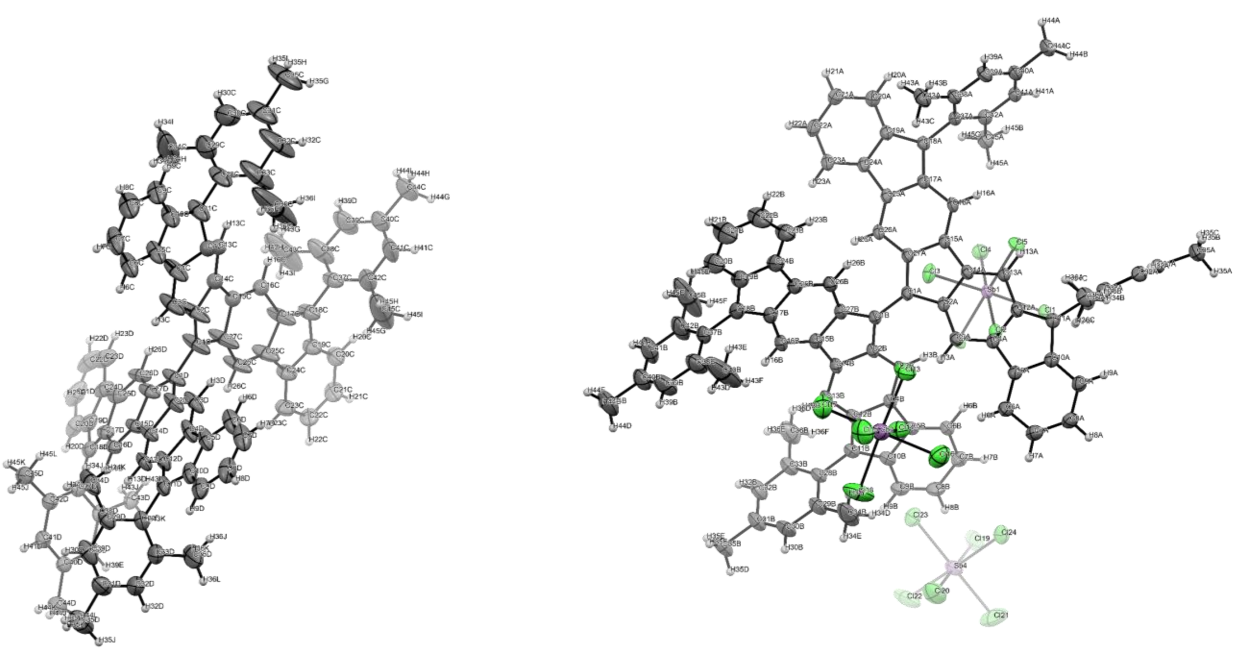

Supplementary Figure 32. ORTEP plot for [**4a**]$^{2+}$[$SbCl_6^-$]$_2$ (CCDC 2209267). Thermal ellipsoids are at the 50% probability level. Solvent molecules and disordered parts of hexachloroantimonate anions are omitted for clarity.

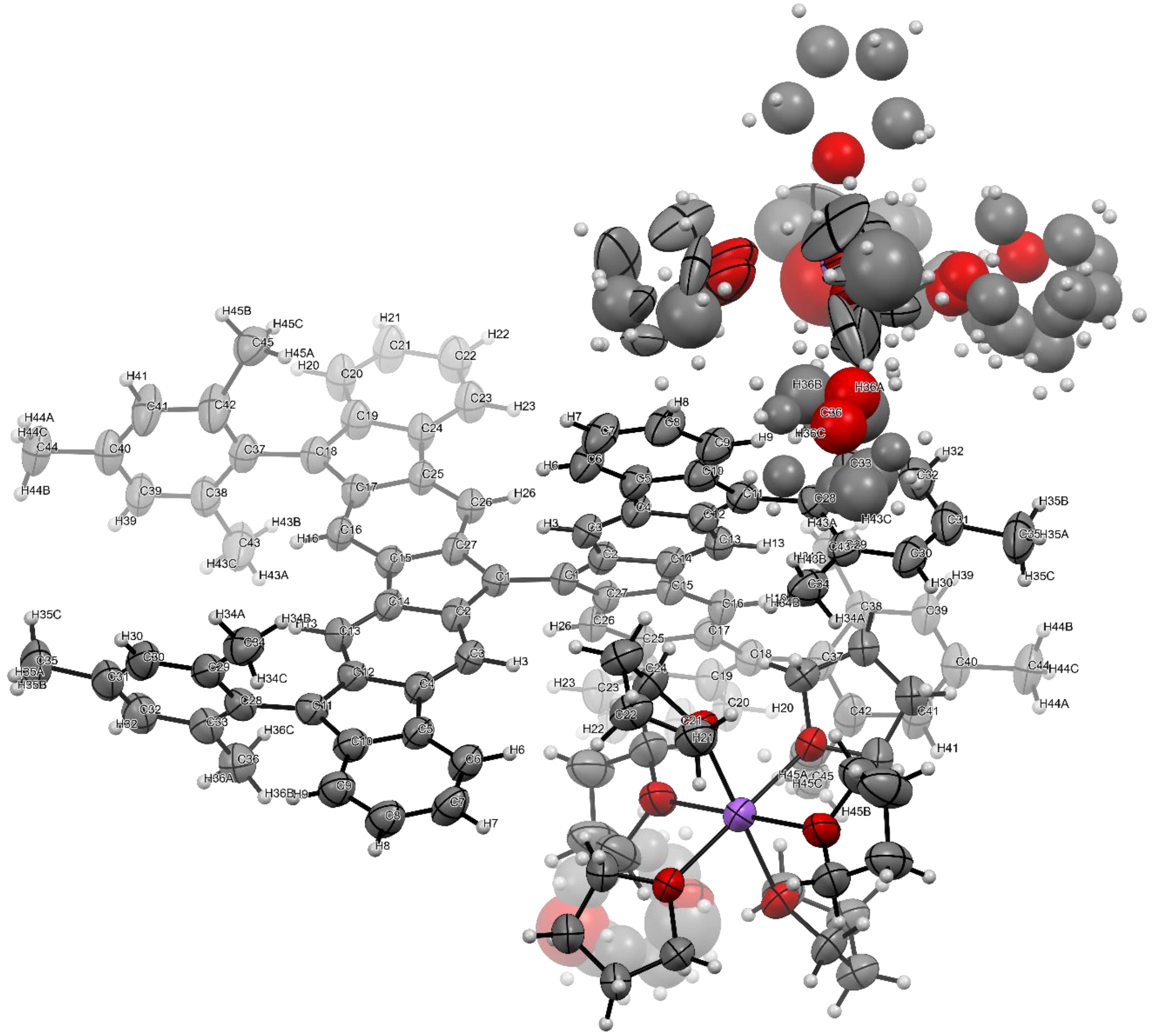

Supplementary Figure 33. ORTEP plot for [Na(THF)$_6$][Na(THF)$_5$]$_{0.74}$[**4a**]·8.3THF (CCDC 2209269). Thermal ellipsoids are at the 30% probability level.

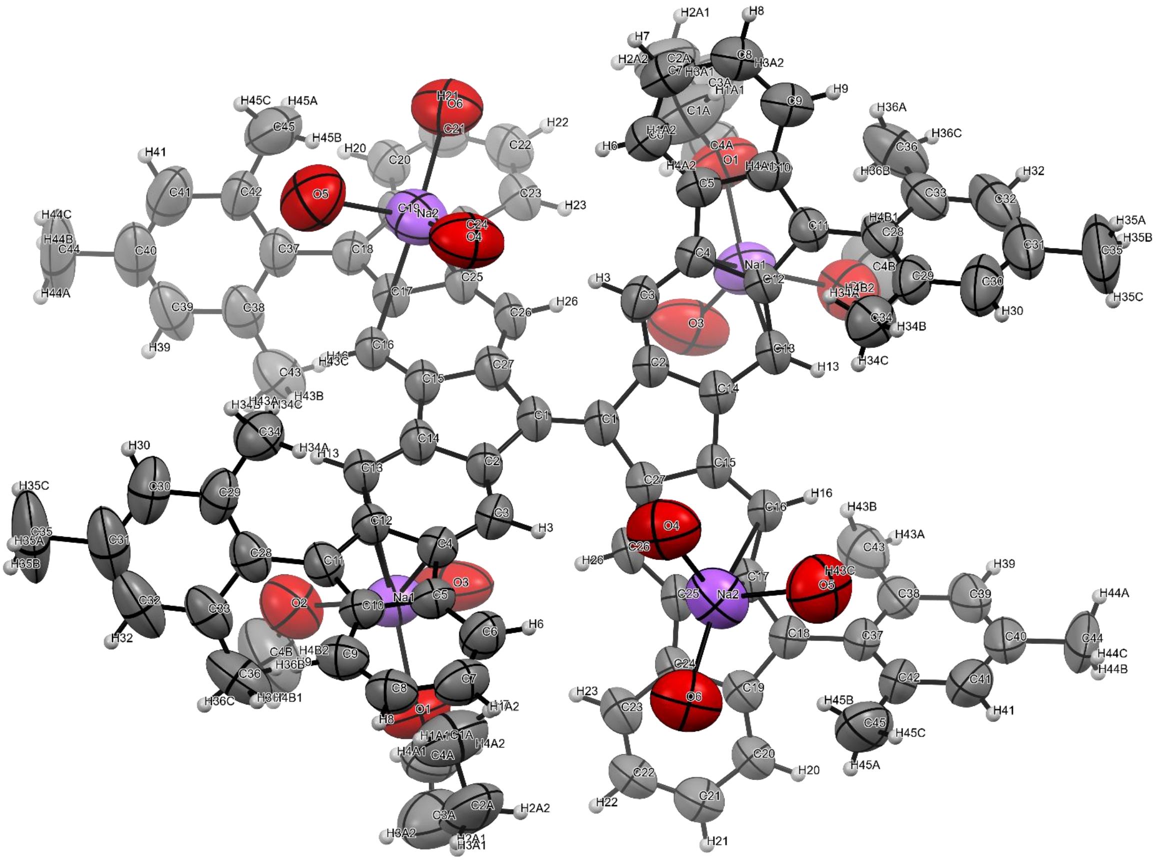

Supplementary Figure 34. ORTEP plot for $[Na(THF)_3]_4$[**4a**] (CCDC 2209268). Thermal ellipsoids are at the 50% probability level. Solvent molecules and disordered parts of coordinated THF ligands are omitted for clarity.

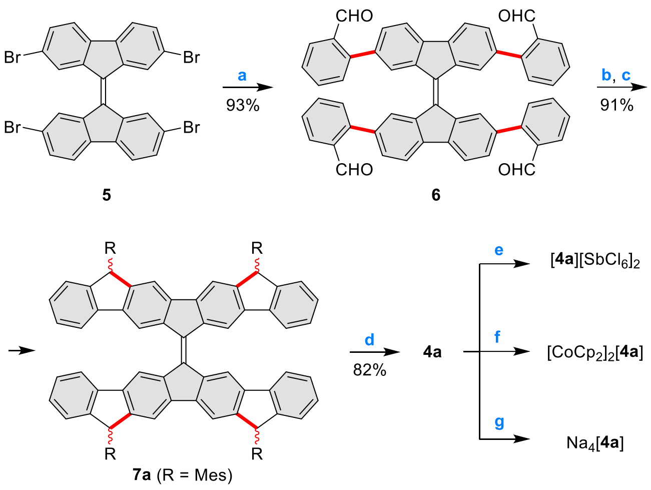

Supplementary Figure 35. Synthesis of tetramesityltetrafluorenofulvalene **4a**. Reagents and conditions: a) 2-formylphenylboronic acid (5.0 equiv), $Pd(PPh_3)_4$ (0.2 equiv), Na_2CO_3 (16.0 equiv), dioxane, H_2O; b) 2-MesMgBr (1 M solution in THF, 8.0 equiv), THF; c) $BF_3{\cdot}Et_2O$, DCM; d) 1. *t*-BuOK (2 M in 2-MeTHF, 8.0 equiv), THF, 2. I_2 (0.1 M in THF, 2.2 equiv).

^{1}H NMR	
3	6.93
6	7.23
7	7.11
8	6.89
9	6.70
13	6.74
17	7.03
19	2.29
20	2.35

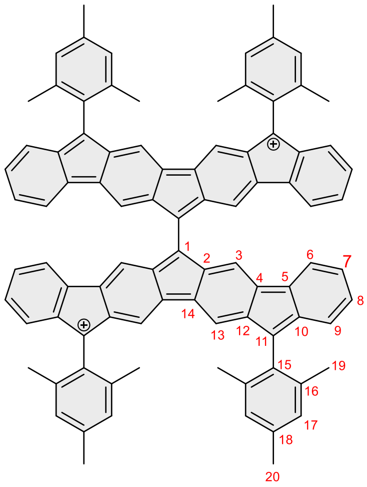

^{13}C NMR	
1	139.53
2	158.96
3	121.15
4	152.11
5	142.59/142.57
6	125.80
7	138.36
8	133.13
9	131.53
10	145.18
11	184.13
12	141.69
13	131.09
14	140.66
15	129.22
16	137.35
17	130.61
18	142.57/142.59
19	22.03
20	22.58

Supplementary Figure 36. ^{1}H and ^{13}C chemical shifts of ($[\mathbf{4a}]^{2+}[SbCl_6^{-}]_2$) (full assignment based on data obtained from COSY, ROESY, HSQC and HMBC experiments).

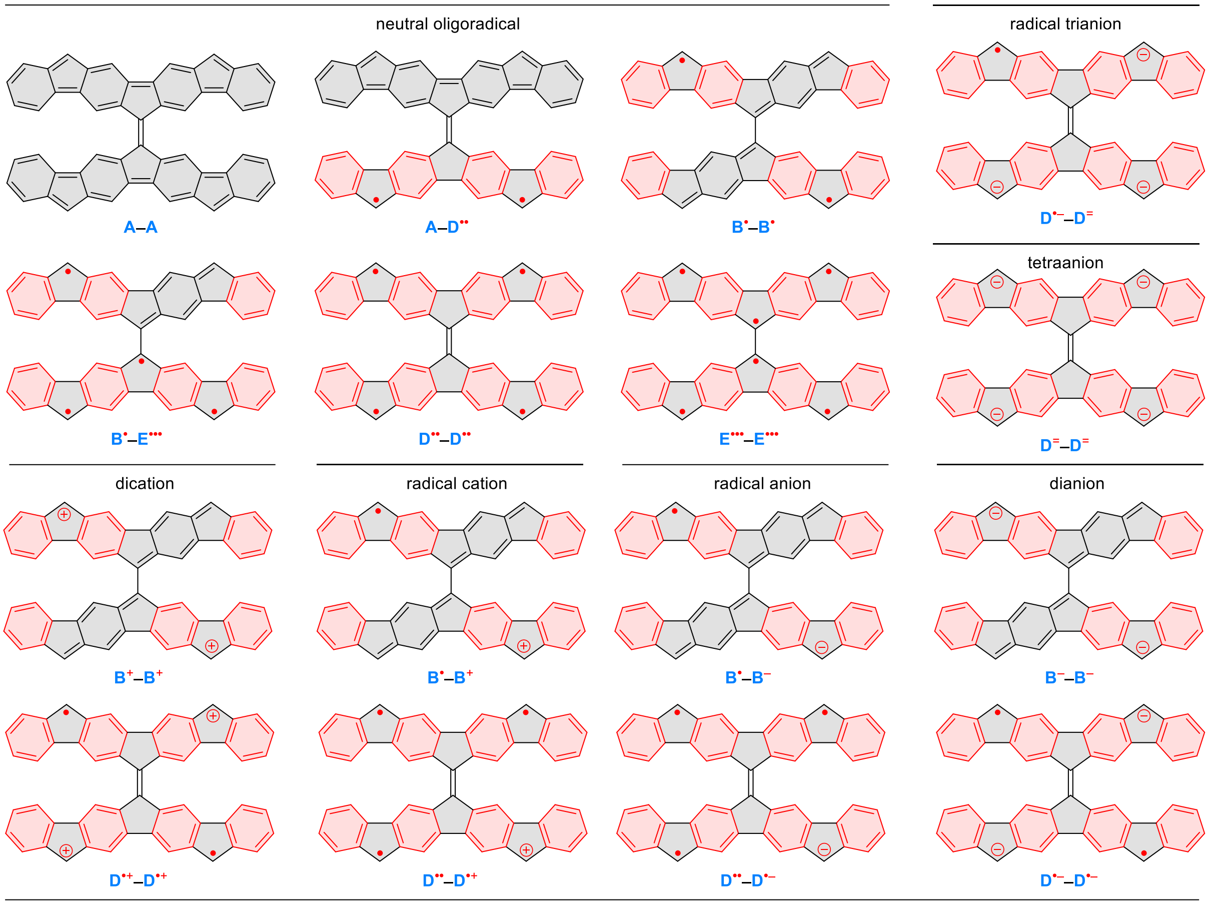

Supplementary Figure 37. Full structures of resonance contributors shown in Figure 6. Flat representations of the TFF core are used for clarity.

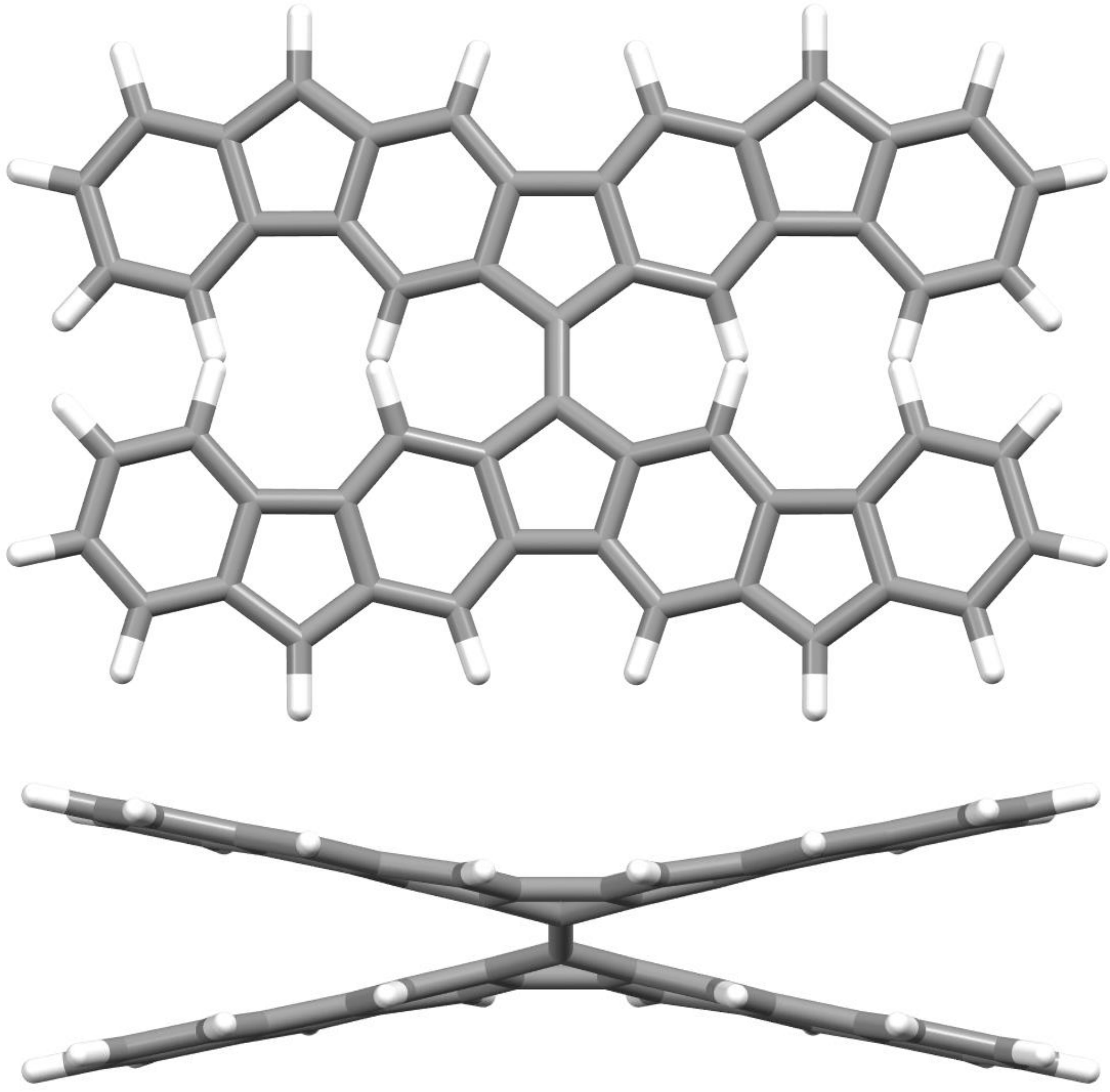

Supplementary Figure 38. Hypothetical folded conformation optimized for ^{1}[**4b**] (CAM-B3LYP/6-31G(d,p)). Similar structures were obtained for other charge and spin states of **1b**, with the exception of ^{3}[**4b**]$^{4-}$, for which no corresponding energy minimum was located. Relative energies of the folded conformers are given in Supplementary Table 1.

Supplementary Tables

Supplementary Table 1. Relative energies, open-shell characteristics, and geometrical parameters for CAM-optimized electronic states of **4b**.

n[a]	m[b]	ΔE[c]	ΔE_{twist}[d]	ΔE_{fold}[e]	n_U[f]		Δn_U[f]		y_0[g]			y_1[g]			y_2[g]		θ_{eq}/°	i/Å[h]	ii/Å[h]	iii/Å[h]	iv/Å[h]
Level:[i]		CAM	CAM	CAM	CAM	CAS	CAM	CAS	CAM	CAS	RAS	CAM	CAS	RAS	CAM	CAS					
0	1		4.7	16.35	3.15	3.01	3.15	3.01	0.987	0.983	0.979	0.371	0.351	0.289	0.118	0.171	46.1	1.439	1.434	1.400	1.443
0	3	3.91	1.1	15.60	2.85		0.85		1.000			0.172			0.170		58.2	1.466	1.419	1.393	1.445
0	5	14.00	15.7	8.23	4.17		0.17		1.000			1.000			0.008		33.7	1.372	1.478	1.429	1.429
1	2		4.6	18.10	2.55		1.55										46.8	1.434	1.433	1.400	1.439
2	1		0.8	25.17	0.42		0.42		0.119			0.058			0.012		59.2	1.463	1.416	1.384	1.442
2	3	11.48	2.7	19.67	2.10		0.10		1.000			0.016			0.007		48.6	1.437	1.432	1.406	1.428
–1	2		6.0	16.28	2.60		1.60										43.6	1.431	1.437	1.400	1.439
–2	1		2.7	22.27	0.58		0.58		0.201			0.069			0.004		51.6	1.457	1.422	1.388	1.434
–2	3	8.97	6.8	16.00	2.09		0.09		1.000			0.017			0.005		42.7	1.431	1.440	1.402	1.430
–3	2		12.2	17.28	1.03		0.03										42.1	1.422	1.451	1.401	1.420
–4	1	0.00	14.3	10.90	0.00		0.00		0.000			0.000			0.000		34.7	1.381	1.488	1.418	1.406
–4	3	14.64	0.0	[j]	2.10		0.10		1.000			0.009			0.008		90.0	1.477	1.443	1.418	1.406

[a] Charge. [b] Multiplicity. [c] SCF energy at the CAM level of theory relative to the singlet state (kcal/mol). [d] Inversion barrier around the center bond, $\Delta E_{twist} = E(\theta = 90°) – E(\theta_{eq})$. [e] SCF energy difference between the folded conformation and the equilibrium twisted conformation of the same charge and multiplicity, $\Delta E_{fold} = E_{fold} – E(\theta_{eq})$. [f] number of unpaired electrons obtained from CAM natural orbital occupation numbers. $\Delta n_U = n_U – (m – 1)$. [g] Oligoradicaloid indices. [h] Averaged over chemically equivalent distances. [i] CAM, UCAM-B3LYP/6-31G(d,p); CAS, CAS-SF(6,6)/6-31G(d,p); RAS, RAS(4,4)-SF-srB3LYP/cc-pVDZ; CAM-optimized geometries used in all cases. [j] No stable energy minimum found corresponding to the folded conformer.

Supplementary Table 2. HOMA Indices calculated for electronic states of **4b**.[a]

Charge	0	0	0	1	-1	2	-2	2	-2	-3	-4	-4
Multiplicity	1	3	5	2	2	1	1	3	3	2	1	3
Ring	Average											
A	0.25	0.30	-0.20	0.27	0.25	0.38	0.22	0.30	0.20	0.01	-0.51	0.07
B	0.71	0.63	0.89	0.70	0.69	0.55	0.55	0.72	0.67	0.67	0.76	0.68
C	0.31	0.28	0.35	0.30	0.34	0.17	0.37	0.28	0.38	0.44	0.42	0.40
D	0.95	0.95	0.93	0.94	0.94	0.93	0.91	0.92	0.90	0.85	0.76	0.76
Ring	esd											
A	0.00	0.00	0.00	0.00	0.01	0.00	0.00	0.08	0.10	0.00	0.00	0.00
B	0.00	0.00	0.00	0.04	0.05	0.00	0.00	0.09	0.07	0.00	0.00	0.00
C	0.00	0.00	0.00	0.03	0.01	0.00	0.00	0.08	0.03	0.00	0.00	0.00
D	0.00	0.00	0.00	0.00	0.01	0.00	0.00	0.00	0.02	0.00	0.00	0.00

[a] Values are averaged for chemically equivalent rings (2 rings for A, 4 rings for B through D). Esds are estimated standard deviations for each group of rings.

Supplementary Table 3. Crystal data and structure refinement for **4a**.

Identification code	mda011	
Empirical formula	$C_{90}H_{68}{\cdot}C_6H_{14}$	
Formula weight	1235.61	
Temperature	100(2) K	
Wavelength	1.54184 Å (CuKα)	
Space group	*Ccca*	
Unit cell dimensions	*a* = 28.078(9) Å	α = 90°.
	b = 27.103(9) Å	β = 90°.
	c = 9.069(3) Å	γ = 90°.
Volume	6901(4) $Å^3$	
Z	4	
Density (calculated)	1.189 Mg/ m^3	
Absorption coefficient	0.503 mm^{-1}	
F(000)	2632.0	
Crystal size/mm3	0.240 × 0.180 × 0.090	
Theta range for data collection	6.296 to 151.788°.	
Index ranges	-34 ≤ h ≤ 35, -33 ≤ k ≤ 33, -9 ≤ l ≤ 11	
Reflections collected	53816	
Independent reflections	3572 [Rint = 0.0562, Rsigma = 0.0315]	
Data/restraints/parameters	3572/6/243	
Goodness-of-fit on F2	1.029	
Final R indexes [I>=2sigma (I)]	R1 = 0.0891, wR2 = 0.2574	
R indexes [all data]	R1 = 0.1373, wR2 = 0.3107	
Extinction coefficient	n/a	
Largest diff. peak and hole	0.31 and -0.21 e Å-3	

Supplementary Table 4. Crystal data and structure refinement for [**4a**]$^{2+}$[$SbCl_6^-$]$_2$.

Identification code	mdadmo	
Empirical formula	$C_{98.57} H_{75} Cl_{28.73} D_{5.57} Sb_2$	
Formula weight	2532.51	
Temperature	100(2) K	
Wavelength	0.71073 Å	
Crystal system	Triclinic	
Space group	P-1	
Unit cell dimensions	a = 12.313(2) Å	α = 94.79(2)°.
	b = 24.286(3) Å	β = 92.54(2)°.
	c = 36.106(4) Å	γ = 91.44(2)°.
Volume	10744(3) Å^3	
Z	4	
Density (calculated)	1.566 mg/m^3	
Absorption coefficient	1.262 mm^{-1}	
F(000)	5049	
Crystal size	0.360 x 0.220 x 0.200 mm^3	
Theta range for data collection	1.656 to 25.500°.	
Index ranges	-14<=h<=14, -28<=k<=29, -43<=l<=42	
Reflections collected	144987	
Independent reflections	39967 [R(int) = 0.0252]	
Completeness to theta = 25.500°	100.0 %	
Absorption correction	Semi-empirical from equivalents	
Max. and min. transmission	1.00000 and 0.84205	
Refinement method	Full-matrix least-squares on F^2	
Data / restraints / parameters	39967 / 474 / 2676	
Goodness-of-fit on F^2	1.031	
Final R indices [I>2sigma(I)]	R1 = 0.0712, wR2 = 0.1992	
R indices (all data)	R1 = 0.0783, wR2 = 0.2077	
Extinction coefficient	n/a	
Largest diff. peak and hole	1.548 and -1.327 e.Å^{-3}	

Supplementary Table 5. Crystal data and structure refinement for [**4a**]$^{2-}$[Na^+]$_2$.

Identification code	mdtan2	
Empirical formula	C_{162} H_{212} $Na_{1.74}$ O_{18}	
Formula weight	2487.31	
Temperature	100(2) K	
Wavelength	1.54184 Å	
Crystal system	Monoclinic	
Space group	C2/c	
Unit cell dimensions	a = 24.365(13) Å	α = 90°.
	b = 22.688(13) Å	β = 92.96(4)°.
	c = 25.027(12) Å	γ = 90°.
Volume	13816(13) Å^3	
Z	4	
Density (calculated)	1.196 mg/m^3	
Absorption coefficient	0.642 mm^{-1}	
F(000)	5389	
Crystal size	0.200 x 0.130 x 0.090 mm^3	
Theta range for data collection	2.663 to 74.083°.	
Index ranges	-29<=h<=28, -27<=k<=26, -30<=l<=29	
Reflections collected	42863	
Independent reflections	13346 [R(int) = 0.0418]	
Completeness to theta = 67.000°	99.6 %	
Absorption correction	Semi-empirical from equivalents	
Max. and min. transmission	1.00000 and 0.43538	
Refinement method	Full-matrix least-squares on F^2	
Data / restraints / parameters	13346 / 743 / 917	
Goodness-of-fit on F^2	1.390	
Final R indices [I>2sigma(I)]	R1 = 0.1113, wR2 = 0.2670	
R indices (all data)	R1 = 0.2348, wR2 = 0.3134	
Extinction coefficient	n/a	
Largest diff. peak and hole	0.311 and -0.292 e.Å^{-3}	

Supplementary Table 6. Crystal data and structure refinement for [**4a**]$^{4-}$[Na^+]$_4$.

Identification code	mdatd	
Empirical formula	$C_{147}H_{185}Na_4O_{12}$	
Formula weight	2235.90	
Temperature	100(2) K	
Wavelength	1.54184 Å	
Crystal system	Monoclinic	
Space group	C2/c	
Unit cell dimensions	a = 49.516(9) Å	α = 90°.
	b = 9.700(2) Å	β = 134°.
	c = 37.374(5) Å	γ = 90°.
Volume	12913(9) Å^3	
Z	4	
Density (calculated)	1.150 mg/m^3	
Absorption coefficient	0.667 mm^{-1}	
F(000)	4828	
Crystal size	0.150 x 0.140 x 0.130 mm^3	
Theta range for data collection	2.373 to 73.486°.	
Index ranges	-59<=h<=60, -11<=k<=11, -44<=l<=45	
Reflections collected	41585	
Independent reflections	12489 [R(int) = 0.0420]	
Completeness to theta = 67.000°	99.8 %	
Absorption correction	Semi-empirical from equivalents	
Max. and min. transmission	1.00000 and 0.58129	
Refinement method	Full-matrix least-squares on F^2	
Data / restraints / parameters	12489 / 60 / 772	
Goodness-of-fit on F^2	1.657	
Final R indices [I>2sigma(I)]	R1 = 0.1376, wR2 = 0.3208	
R indices (all data)	R1 = 0.2414, wR2 = 0.3531	
Extinction coefficient	n/a	
Largest diff. peak and hole	0.539 and -0.363 e. Å^{-3}	

NMR Spectra

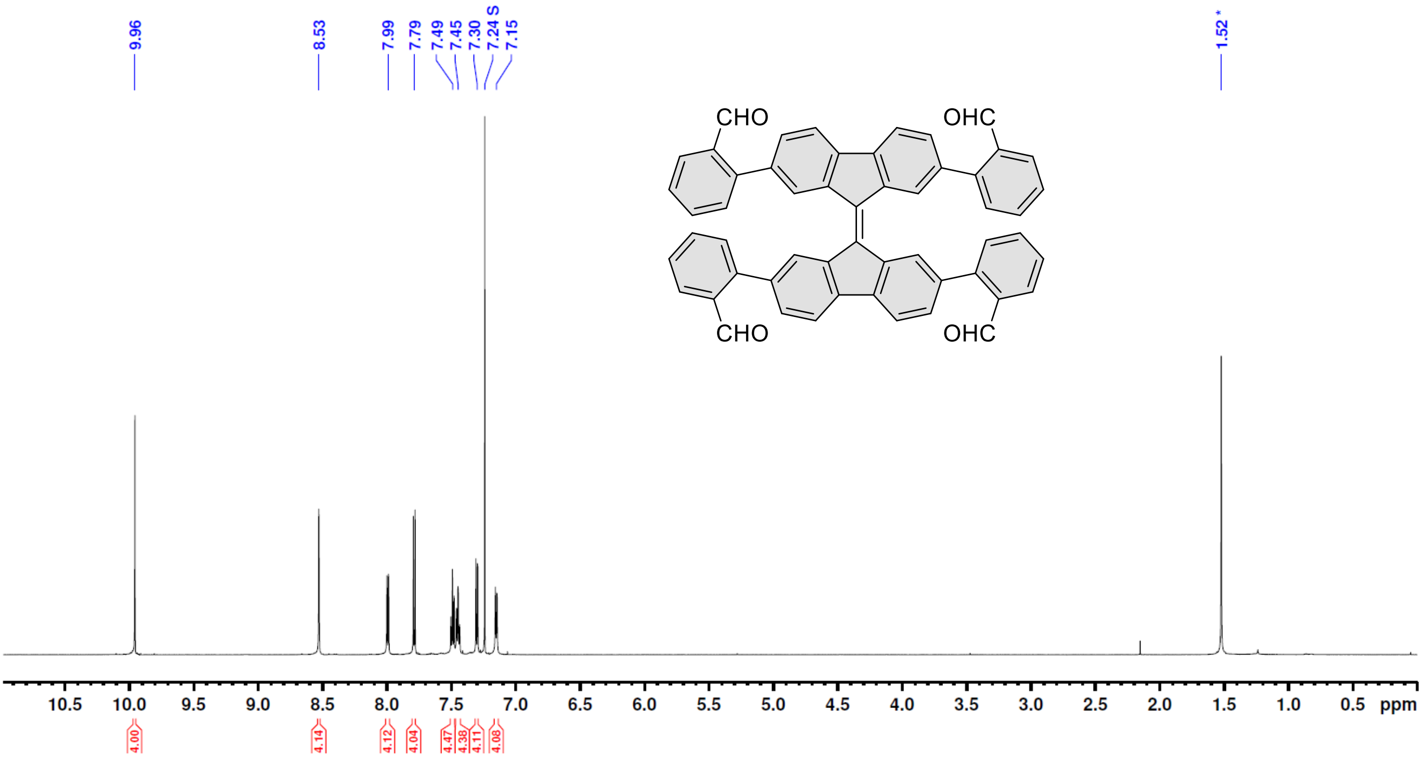

Supplementary Figure 39. ^{1}H NMR spectrum of compound **6** (600 MHz, chloroform-*d*, 300 K).

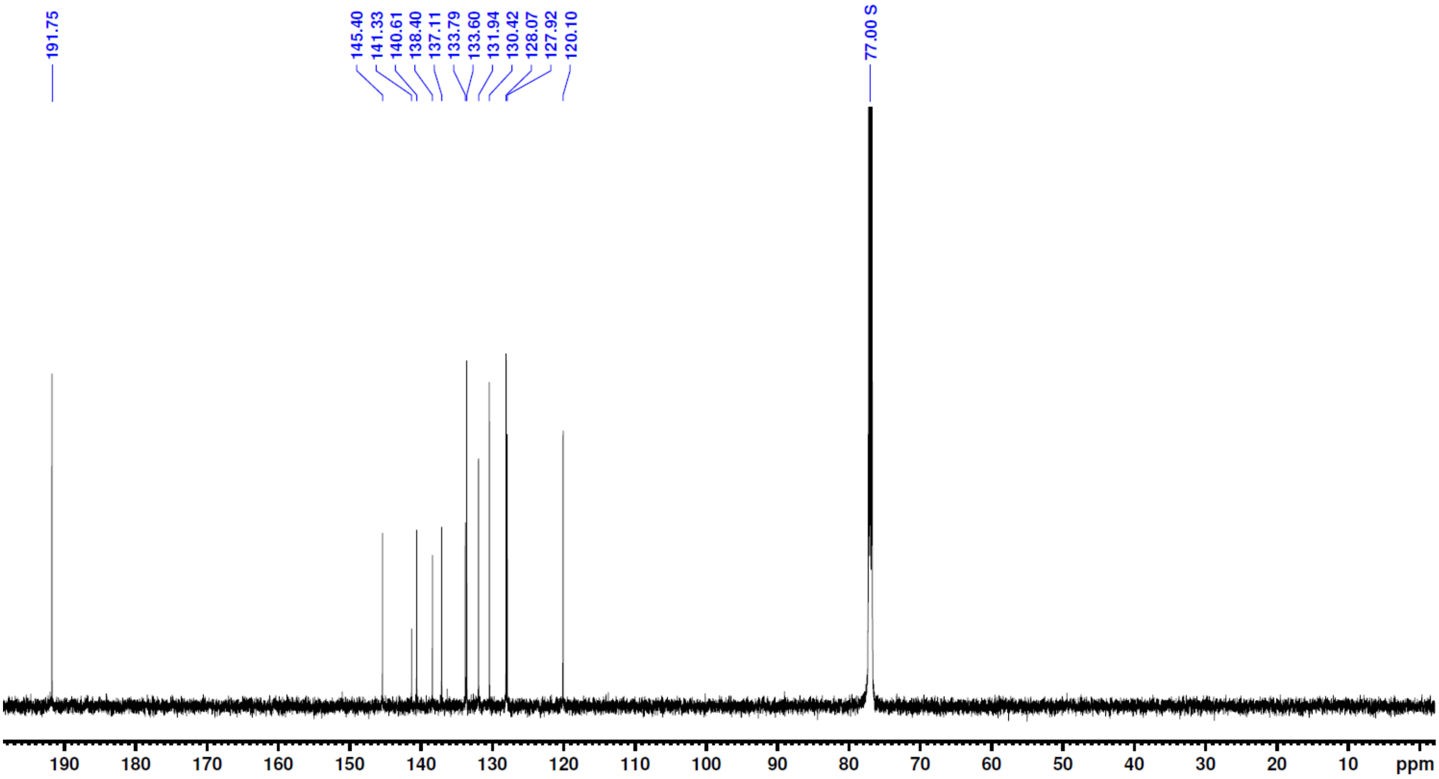

Supplementary Figure 40. ^{13}C NMR spectrum of compound **6** (151 MHz, chloroform-*d*, 300 K).

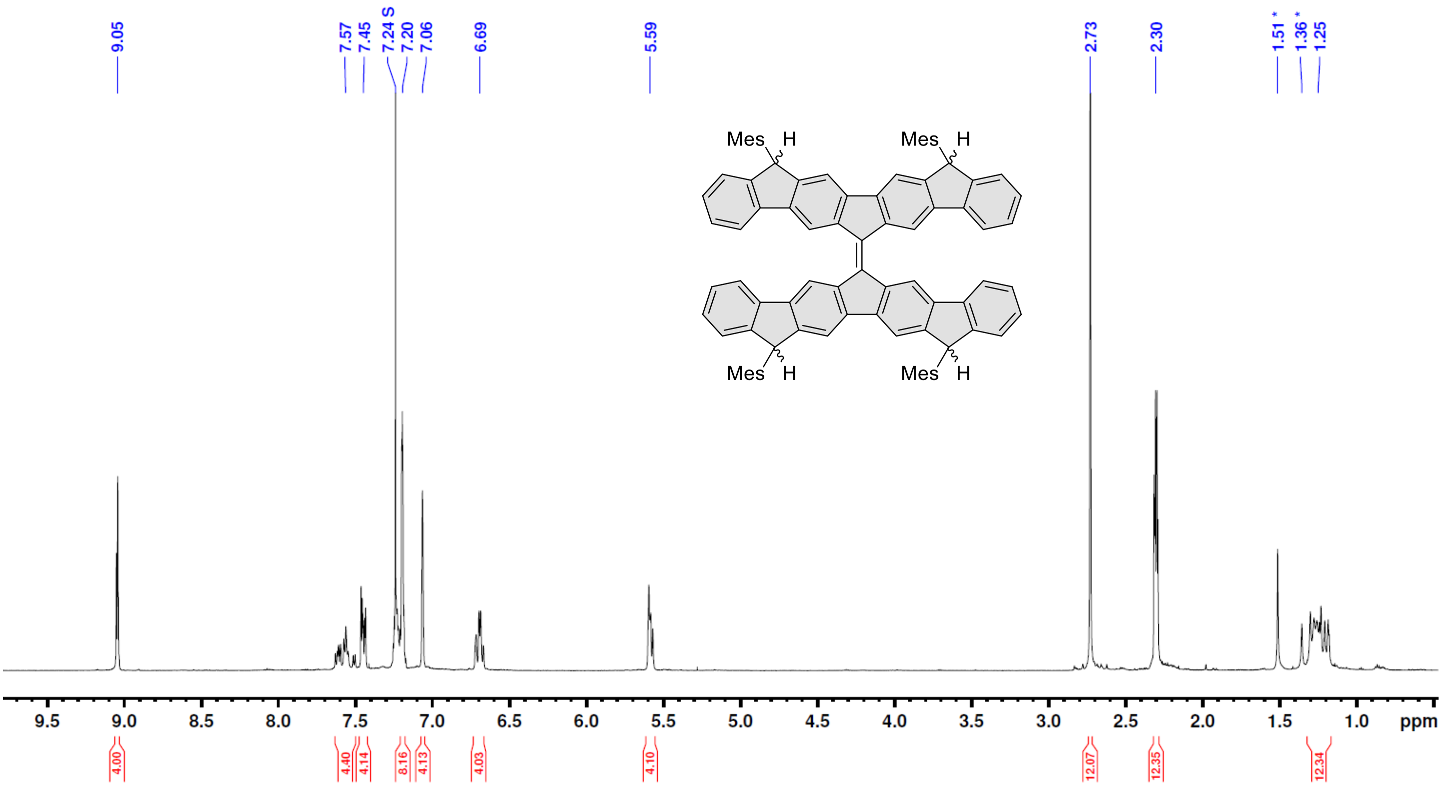

Supplementary Figure 41. ^{1}H NMR spectrum of compound **7a** (600 MHz, chloroform-*d*, 300 K).

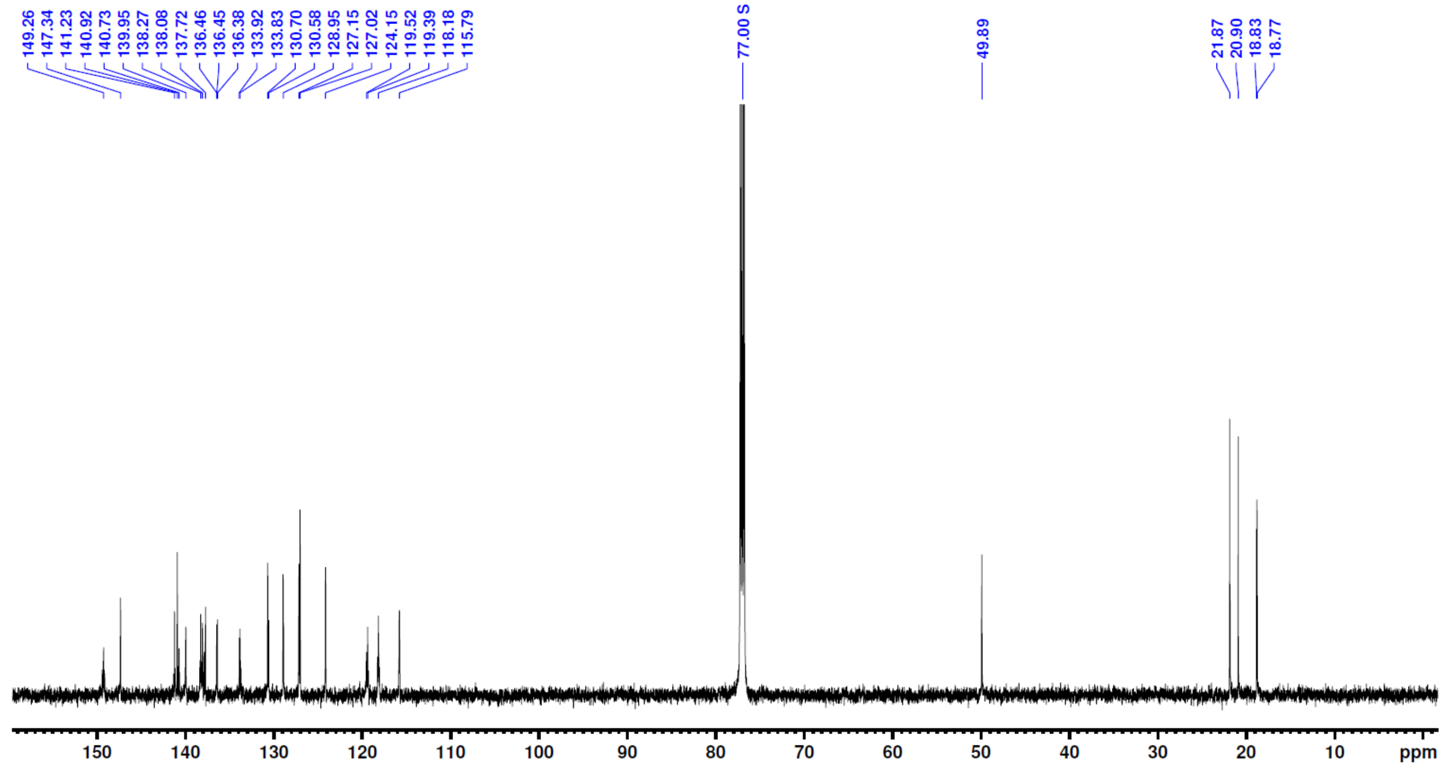

Supplementary Figure 42. ^{13}C NMR spectrum of compound **7a** (151 MHz, chloroform-*d*, 300 K).

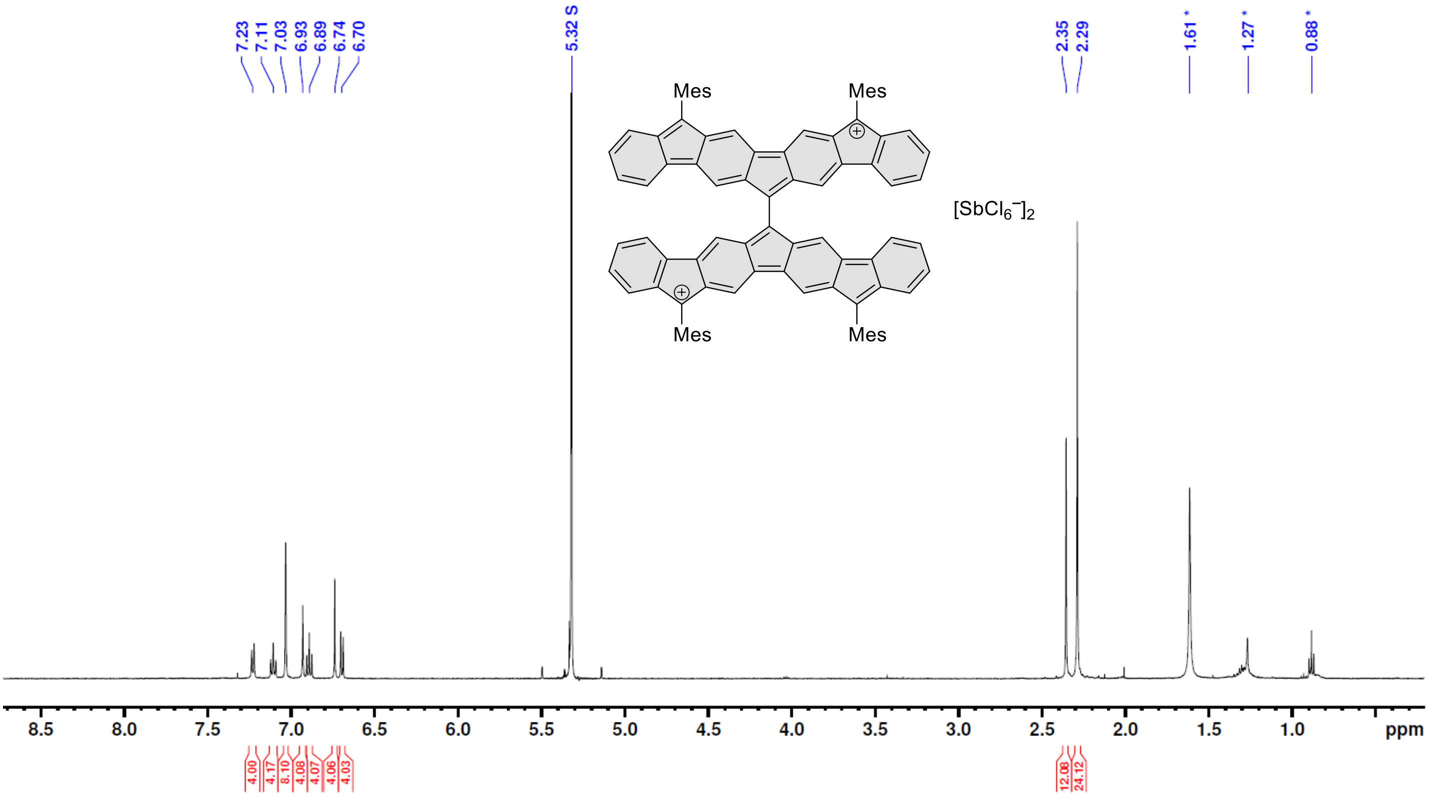

Supplementary Figure 43. ^{1}H NMR spectrum of compound [**4a**]$^{2+}$[$SbCl_6^-$]$_2$ (500 MHz, dichloromethane-d_2, 300 K).

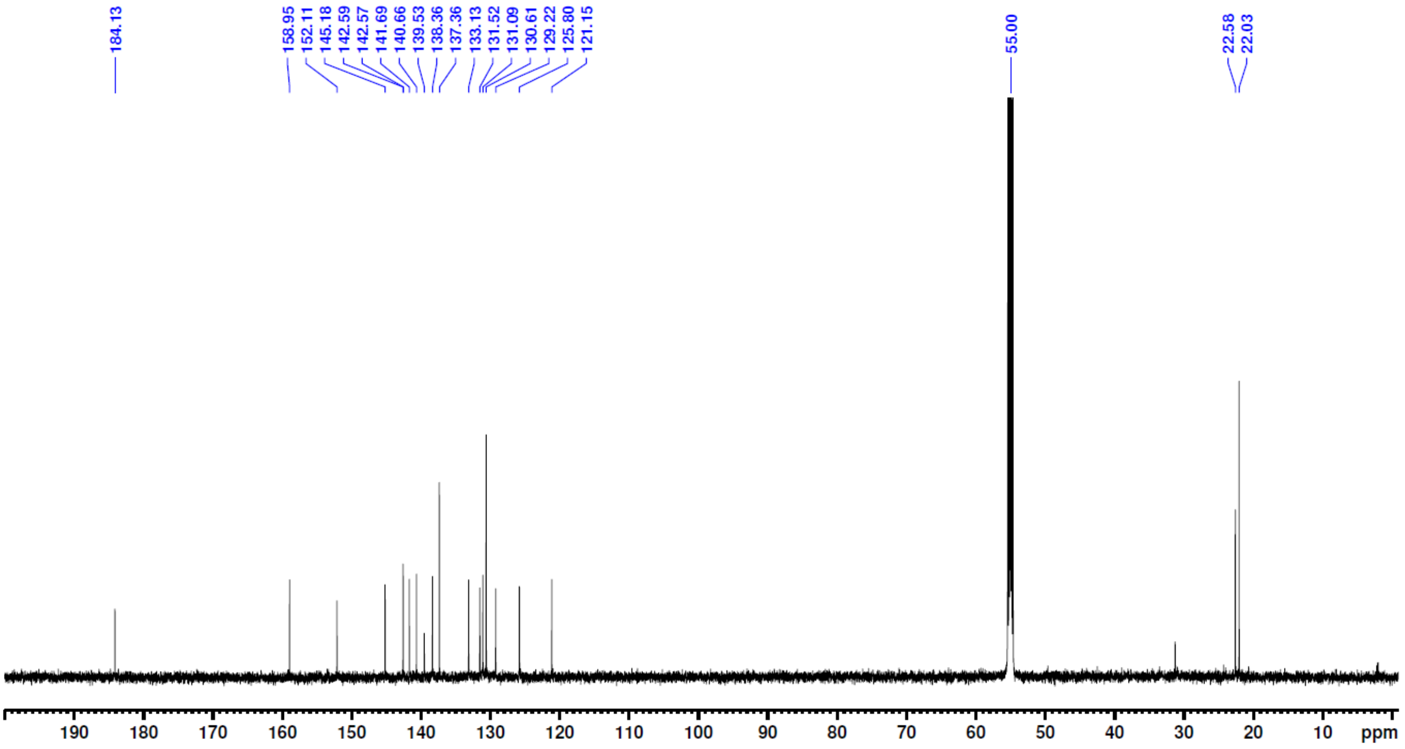

Supplementary Figure 44. ^{13}C NMR spectrum of compound **[4a]$^{2+}$[$SbCl_6^-$]$_2$** (151 MHz, dichloromethane-d_2, 300 K).

Mass Spectra

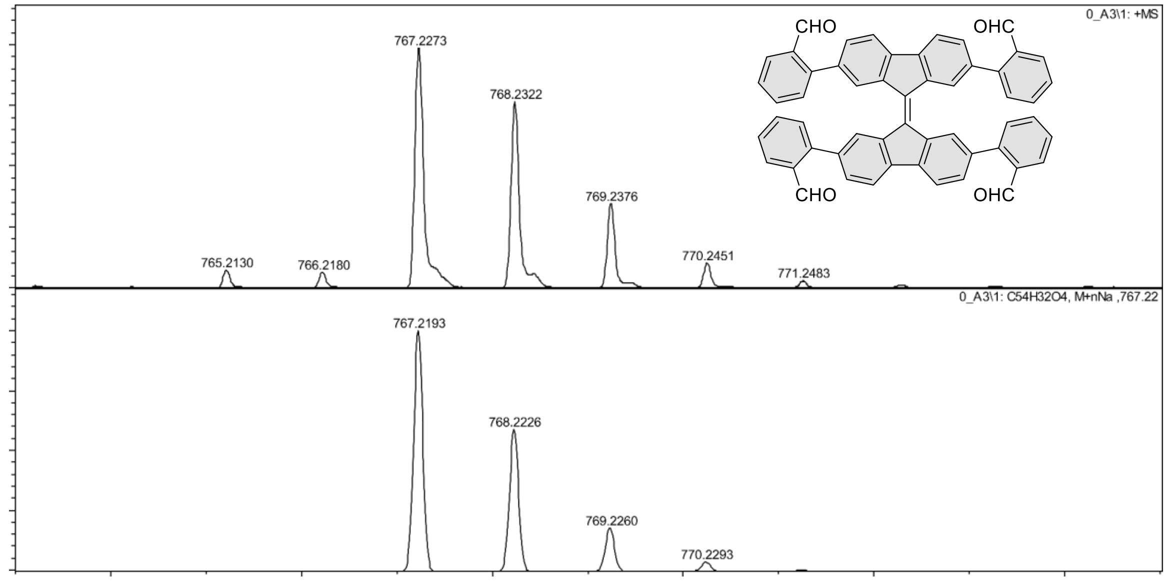

Supplementary Figure 45. High resolution mass spectrum of **6** (MALDI–TOF, top: experimental, bottom: simulated).

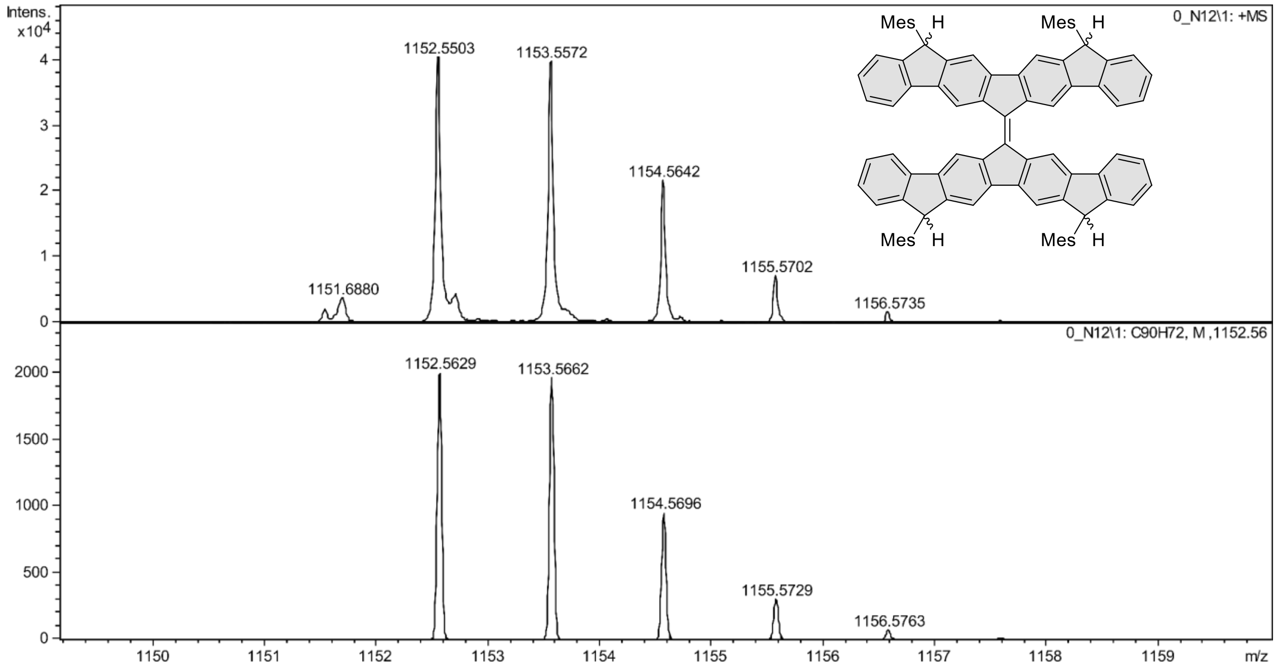

Supplementary Figure 46. High resolution mass spectrum of **7a** (MALDI–TOF, top: experimental, bottom: simulated).

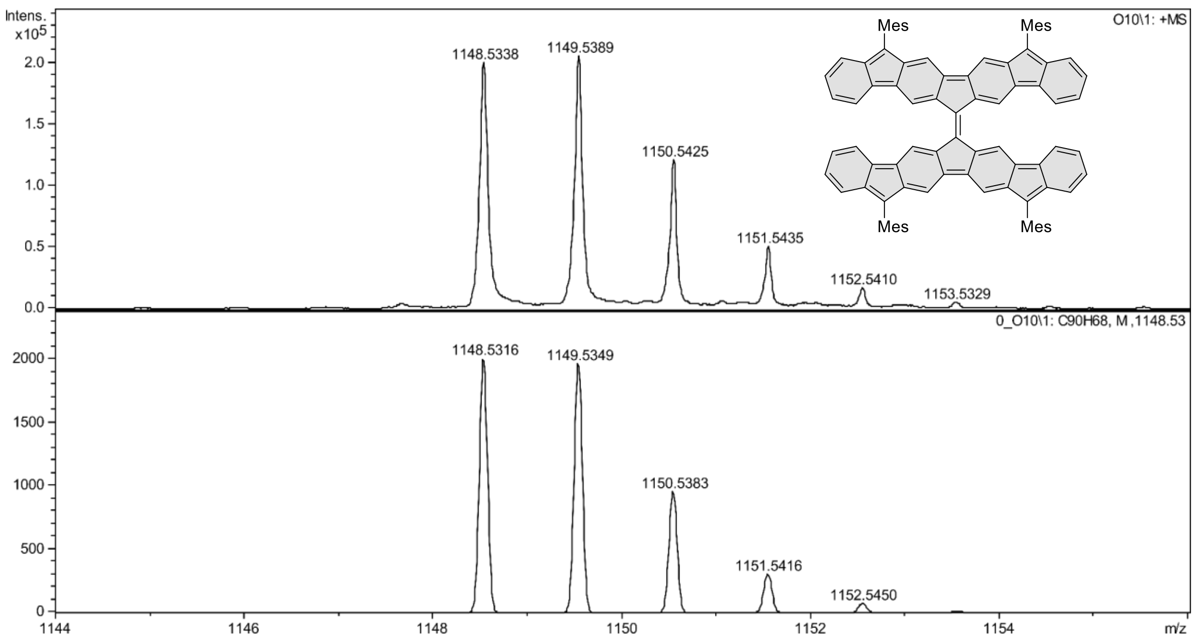

Supplementary Figure 47. High resolution mass spectrum of **4a** (MALDI–TOF, top: experimental, bottom: simulated).

References

1. Zhao, Y. *et al.* 9,9′-Bifluorenylidene-Core Perylene Diimide Acceptors for As-Cast Non-Fullerene Organic Solar Cells: The Isomeric Effect on Optoelectronic Properties. *Chemistry – A European Journal* **24**, 4149–4156 (2018).
2. Bain, G. A. & Berry, J. F. Diamagnetic Corrections and Pascal's Constants. *J. Chem. Educ.* **85**, 532 (2008).
3. Frisch, M. J. *et al.* Gaussian 16, Revision B.01. (2016).
4. Becke, A. D. Density-functional exchange-energy approximation with correct asymptotic behavior. *Phys. Rev., A* **38**, 3098–3100 (1988).
5. Becke, A. D. Density-functional thermochemistry. III. The role of exact exchange. *J. Chem. Phys.* **98**, 5648–5652 (1993).
6. Lee, C., Yang, W. & Parr, R. G. Development of the Colle-Salvetti correlation-energy formula into a functional of the electron density. *Phys. Rev. B* **37**, 785–789 (1988).
7. Yanai, T., Tew, D. P. & Handy, N. C. A new hybrid exchange–correlation functional using the Coulomb-attenuating method (CAM-B3LYP). *Chemical Physics Letters* **393**, 51–57 (2004).
8. Grimme, S., Ehrlich, S. & Goerigk, L. Effect of the damping function in dispersion corrected density functional theory. *Journal of Computational Chemistry* **32**, 1456–1465 (2011).
9. Nobusue, S. *et al.* Tetracyclopenta[def,jkl,pqr,vwx]tetraphenylene: A Potential Tetraradicaloid Hydrocarbon. *Angew. Chem. Int. Ed.* **54**, 2090–2094 (2015).
10. Dunning, T. H. Gaussian basis sets for use in correlated molecular calculations. I. The atoms boron through neon and hydrogen. *J. Chem. Phys.* **90**, 1007–1023 (1989).
11. Zimmerman, P. M., Bell, F., Goldey, M., Bell, A. T. & Head-Gordon, M. Restricted active space spin-flip configuration interaction: Theory and examples for multiple spin flips with odd numbers of electrons. *The Journal of Chemical Physics* **137**, 164110 (2012).
12. Bell, F., Zimmerman, P. M., Casanova, D., Goldey, M. & Head-Gordon, M. Restricted active space spin-flip (RAS-SF) with arbitrary number of spin-flips. *Phys. Chem. Chem. Phys.* **15**, 358–366 (2013).

13. Casanova, D. Short-range density functional correlation within the restricted active space CI method. *The Journal of Chemical Physics* **148**, 124118 (2018).
14. Chen, Z., Wannere, C. S., Corminboeuf, C., Puchta, R. & Schleyer, P. von R. Nucleus-Independent Chemical Shifts (NICS) as an Aromaticity Criterion. *Chem. Rev.* **105**, 3842–3888 (2005).
15. Kruszewski, J. & Krygowski, T. M. Definition of aromaticity basing on the harmonic oscillator model. *Tetrahedron Letters* **13**, 3839–3842 (1972).
16. Krygowski, T. M., Szatylowicz, H., Stasyuk, O. A., Dominikowska, J. & Palusiak, M. Aromaticity from the Viewpoint of Molecular Geometry: Application to Planar Systems. *Chem. Rev.* **114**, 6383–6422 (2014).
17. Nakano, M. Electronic Structure of Open-Shell Singlet Molecules: Diradical Character Viewpoint. *Top Curr Chem (Z)* **375**, 47 (2017).
18. Head-Gordon, M. Characterizing unpaired electrons from the one-particle density matrix. *Chemical Physics Letters* **372**, 508–511 (2003).
19. Jacquemin, D., Wathelet, V., Perpète, E. A. & Adamo, C. Extensive TD-DFT Benchmark: Singlet-Excited States of Organic Molecules. *Journal of Chemical Theory and Computation* **5**, 2420–2435 (2009).
